# Theoretical Insights Into the Depolymerization Mechanism of Lignin to Methyl *p*-hydroxycinnamate by [Bmim][FeCl_4_] Ionic Liquid

**DOI:** 10.3389/fchem.2019.00446

**Published:** 2019-06-18

**Authors:** Tian Zhang, Yaqin Zhang, Yanlei Wang, Feng Huo, Zhangmin Li, Qiang Zeng, Hongyan He, Xuehui Li

**Affiliations:** ^1^State Key Laboratory of Pulp and Paper Engineering, School of Chemistry and Chemical Engineering, South China University of Technology, Guangzhou, China; ^2^Beijing Key Laboratory of Ionic Liquids Clean Process, CAS Key Laboratory of Green Process and Engineering, State Key Laboratory of Multiphase Complex Systems, Institute of Process Engineering, Chinese Academy of Sciences, Beijing, China

**Keywords:** lignin, metallic ionic liquid, biomass, reaction mechanism, DFT

## Abstract

Depolymerization of lignin into valuable aromatic compounds is an important starting point for its valorization strategies, which requires the cleavage of C-O and C-C bonds between lignin monomer units. The catalytic cleavage of these bonds is still difficult and challenging. Our previous experimental investigation (Green Chem., 2018, 20: 3743) has shown that methyl p-hydroxycinnamate (**MPC**) can be produced from molecular tailoring of H unit in lignin by the cleavage of the γ-O ester bond. In this study, the mechanism of [Bmim][FeCl_4_]-catalyzed depolymerization of lignin was investigated by using the density functional theory (DFT) method. The results reveal that [FeCl_4_]^−^ anion of the catalyst plays a decisive role in the whole catalytic process, where two possible activation modes including three different potential reaction pathways can realize the depolymerization of lignin model compound. The calculated overall barriers of the catalytic conversion along these potential routes show that the third potential pathway, i.e., methanol firstly activated by [Bmim][FeCl_4_], has the most probability with the lowest energy barrier, while the second pathway is excluded because the energy barrier is too high. Also, the results illustrate that the solvent effect is beneficial to the reduction of the relative energy for the reaction to form the transition states. Hence, the obtained molecular level information can identify the favorable conversion process catalyzed by metallic ionic liquids to a certain extent, and it is desirable to enhance the utilization of biomass as a ubiquitous feedstock.

## Introduction

**Scheme 1:**
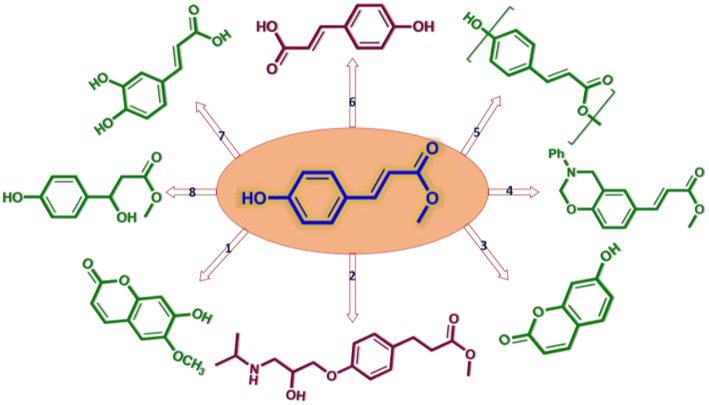
Schematic diagram of chemicals with MPC as platform compound (Li et al., 2018).

Methyl p-hydroxycinnamate (**MPC**) is a promising platform chemical for a variety of fine chemicals, which can be used as valuable medicinal intermediates (compounds 1–3 in Scheme 1; Lin et al., 2013; Yang et al., 2015) and polymeric precursor monomers (compounds 4, 5 in Scheme 1; Tilman et al., 2009; Ji et al., 2012). It has been reported that **MPC** possesses a wide variety of biological properties such as anti-tumor, anti-inflammatory, anti-adipogenic, and neuroprotective activities (compounds 6–8 in Scheme 1; Lee et al., 2013; Vo et al., 2014). The current **MPC** production mostly relies on high-cost petrochemical feedstock, and corrosive acid or noble metal catalysts with complicated separation processes (Clark et al., 2000; Percec et al., 2006; Guzman et al., 2014). In this regard, finding a more environmentally friendly and lower-cost synthesis method for **MPC** production from renewable resources, such as lignocellulosic biomass, is very attractive with an urgent and challenging necessity and it can better meet the requirements of sustainable development as well.

Lignocellulosic biomass mainly comprises cellulose, hemicellulose, and lignin. Among these three constituents, lignin is the least used resource due to its complexity and recalcitrance to chemical process. However, lignin contains a large number of ring structures, including three monolignol precursors, i.e., p-coumaryl alcohol (H), coniferyl alcohol (G), and sinapyl alcohol (S), with multiple C-O-C (ester, β-O-4′, α-O-4′, 4-O-5′), and C-C (β-β′, β-5′, β-1′, 5-5′) linkages. This advantage makes lignin highly potential to be a renewable source for various aromatic compounds. That is to say, it is critical to make full use of lignin by converting it into value-added chemicals. Catalytic depolymerizations, including catalytic oxidation, alcoholysis, and hydrogenolysis, are the most efficient ways to do so. These techniques always focus on the cleavage of C-O-C and C-C bonds to obtain low molecular weight products. However, the products are composed of a wide range of chemical compounds. To avoid this disadvantage and make the product more suitable for direct use as either industrial feedstock or fuel, more recently Li et al. (2018) proposed a remarkable method, that is different from previous C-O or C-C cleavage approaches. In particular, the selective catalytic tailoring the H unit (*p*CA) in herbaceous lignin for **MPC** production can be realized over metal-based ionic liquids (MBILs), as **MPC** has a similar structure to that of p-coumaric acid ester, a typical H structural unit in herbaceous lignin. In this case, the isolated yield of **MPC** upgraded to 71.1 mg g^−1^ under optimized reaction conditions with one of the MBILs, [Bmim][FeCl_4_]. While the calculation results of the narrow HOMO-LUMO energy gap between ester lignin and [FeCl_4_]^−^ anion preliminarily proved its superiority, the detailed reaction mechanism was not further provided.

**Scheme 2:**
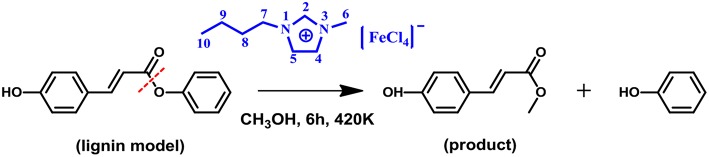
Conversion of lignin model compound to **MPC** according to Otera (1993) and Hoydonckx et al. (2004).

It is well known that, ionic liquids (ILs), as a new kind of catalyst, have received significant attention in the field of biomass conversion owing to their high potential for the replacement of conventional acidic catalysts (Yang et al., 2017; Zhang et al., 2017). Hence, such a catalytic conversion from herbaceous lignin to **MPC** by the MBILs and similar strategy using ILs as the catalyst can be regarded as ground-breaking technologies, deserving more attention. In particular, it is noted that theoretical studies on lignin catalytic conversion with ILs are relatively rare, while considerable attention has been mainly focused on experimental studies. Our previous work illustrated that the geometric and interactions between 16 ILs and lignin model compound GG, and then identified sulfonic ILs having the strongest interaction with GG (Zhang et al., 2017). Further, we investigated the reaction mechanism of the cleavage of β-O-4 bond in GG to guaiacol by -SO_3_H functionalized ILs (Zhang et al., 2019). Given the versatility of IL catalysts (He et al., 2015; Wang et al., 2015; Meng et al., 2017; Qian et al., 2018; Zhang et al., 2018) and our keen interest in the research of biomass (Cai et al., 2015; Suresh et al., 2017), an elegant approach for catalytic transform of lignin to structurally defined aromatic esters is put forward here. According to Otera (1993) and Hoydonckx et al. (2004), the lignin model compound, phenyl p-hydroxycinnamate (**PPC**), was used to produce **MPC** by cleavage of the γ-O ester linkage using MBIL [Bmim][FeCl_4_] as catalyst (Scheme 2). The reaction mechanism was also raised by DFT calculations in details. By understanding all elementary steps involved in the reaction network mentioned above, we constructed a potential energy surface for all reaction routes and proposed a dominant reaction pathway, which will provide a guide for other researchers who are interested in developing efficient ways of depolymerizing lignin.

## Computational Details

Scheme 2 shows the model system studied in this work and describes the conversion process of lignin model compound **PPC** to **MPC** catalyzed by [Bmim][FeCl_4_] MBIL catalyst. All calculations were carried out under the framework of density functional theory at the B3LYP-D3 level (Stephens et al., 1994; Grimme et al., 2010) in Gaussian 09 D.01 program package (Frisch et al., 2013), including the optimization of the structures of all the reactants, products, intermediates, and transition states. The basis set SDD (Andrae et al., 1990; Igel-Mann et al., 2006) was used for Fe atom, and the 6-31+G(d,p) basis set (Harihara and Pople, 1973; Frisch et al., 1984) was applied for all the other atoms, which is referred to as B3LYP-D3/BSI hereafter, vibrational frequency analysis based on the optimized structures was carried out to verify the natures of all the stationary points (local minimum or first-order saddle point). Transition states were validated by performing intrinsic reaction coordinate (IRC) (Fukui, 1981; Barone and Cossi, 1998) calculations to ensure that each of them actually connected the desired reactant and product. Structures at the two ends of IRC paths were optimized to minima, which represent the stable geometrics of reactants and products. The single-point energies of all structures were then improved at M06-D3 /6-311++G(d,p) level (Grimme et al., 2010; Qu et al., 2014) for all atoms except Fe atom (SDD), which is referred to as M06-D3/BSII hereafter. To mimic the solvent effect on the reactions, the solvation model based on density (SMD) (Marenich et al., 2009; Bernales et al., 2012) in methanol solvent was applied for all gas phase structures. The final Gibbs free energies (G_*g*_) in the gas phase are the summation of thermal corrections to gas phase Gibbs free energies (ΔG_*c*_) at the B3LYP-D3/BSI level and the single-point energies from the M06-D3/BSII level (E_*g*_), referred to Equation 1. The solvation Gibbs free energies (G_*s*_) are the sum of ΔG_*c*_ and the single-point energies from the M06-D3(SMD)/BSII level (E_*s*_), referred to Equation 2. In the following content, the G_*s*_ and G_*g*_ were used to draw the relative free energy profiles.

(1) $$\begin{array}{r} G_{g}=E_{g}+\Delta G_{c} \end{array}$$

(2) $$\begin{array}{r} G_{s}=E_{s}+\Delta G_{c} \end{array}$$

When multi-component changes are involved in a reaction, the above-mentioned thermal corrections based on ideal gas phase model will overestimate the contributions of entropy to free energies for reactions with solvent, because the suppressing effect of solvent on the rotational and transitional freedoms of substrates is neglected, which also have been proved by experimental studies (Liang et al., 2008; Huang et al., 2011). In the present study, we adopt the approximate approach proposed by Martin et al. (1998) to correct all solute free energies. A correction of 4.3 kcal/mol applies to per component change for a reaction at 298.15 K and 1 atm, i.e., a reaction from m to n components has an additional correction of (n-m) × 4.3 kcal/mol. Many previous works (Qu et al., 2014, 2015) have also proved the reliability of this protocol. So, we discuss the mechanism in terms of the corrected free energies in the following sections. The Cartesian coordinates and total energies (include in the gas phase and solvent corrected free energies) of all optimized structures are given in the Supporting Information.

## Results and Discussion

**Scheme 3:**
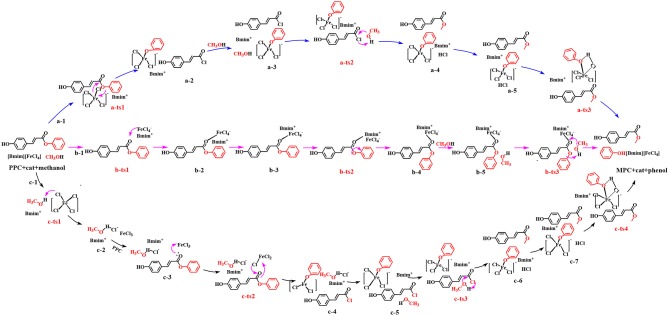
Possible pathways explored for this work.

In this work, the depolymerization from lignin to **MPC** by cleavage of γ-O ester linkage, as catalyzed by MBILs, was performed under mild conditions (420 K) and this process is very different from typical lignin depolymerization by cleavage of the β-O-4 ether linkage. Therefore, we considered a different mechanism from a general reactivity profile (Sabot et al., 2007; Weng et al., 2011). Inspired by the alcoholysis mechanism (Otera, 1993; Hoydonckx et al., 2004), this work has put forward three potential pathways to explore the possible process of lignin depolymerization catalyzed by MBILs, as a complement to a series of researches on lignin (Cai et al., 2015; Long et al., 2016; Suresh et al., 2017). Herein, to visualize the three transformation paths for an in-depth understanding, the pathways are respectively indicated as **a**, **b**, and **c**. In pathway **a**, [FeCl_4_]^−^ anion of catalyst attacks **PPC**, and **MPC** is produced through acyl chlorination. Organic compounds containing ester link or acyl chloride link are ubiquitous, and strongly nucleophilic acyl chloride group compared to hydroxyl groups (Kuroki et al., 1998), can easily be transformed into the complex functionalized with the ester group (Kaynak et al., 2018). The conversion rate will be higher if acylation is used to replace the general alcoholic esterification. Pathway **b** is regarded as a transesterification process (Otera, 1993). This process is widely applied in laboratories and industries and serves as one of the most typical organic reactions. Comparing to esterification, this process is a convenient and practical way to obtain ester by using this reaction when the parent carboxylic acids are unstable or difficult to separate (Hoydonckx et al., 2004). Furthermore, the reactivity of transesterification depends mainly on the electrophilic and nucleophilic properties of the substrates. Thus, pathway **b** currently being adopted here to get **MPC** is to activate the substrates directly by [FeCl_4_]^−^ anion of the catalyst. By contrast, the pathway **c** is considered as an improved acid catalytic conversion process. As known to us, ILs with more Brønsted acid sites exhibit superior catalytic ability in transesterification reactions. Interestingly, structural rearrangement may influence the reduction of the reaction energy barrier. So, several factors responsible for the lignin transformation have been considered in the third path, such as the production of -FeCl_3_ and free Cl^−^ anion, intermolecular rearrangement. The three possible paths are summarized in Scheme 3. In the following, we discuss the mechanism of the transformation in terms of the three pathways.

### Potential Reaction Pathways

#### Pathway a–Acyl Chlorination Process

**Figure 1:**
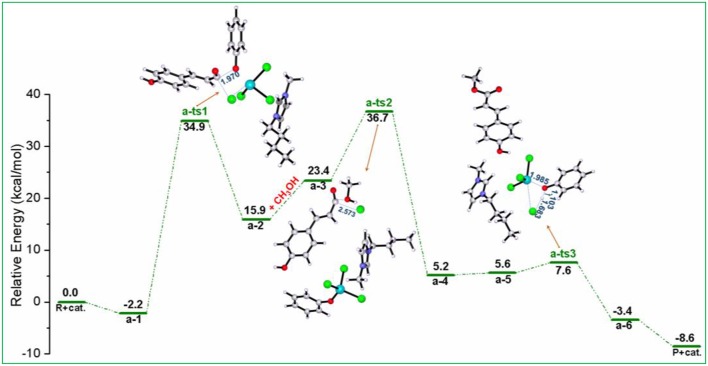
Calculated Gibbs free energy (G_*s*_) profile with optimized geometries of transition states, corresponding to pathway **a** shown in Scheme 3. Distances are given in angstroms.

**Figure 2:**
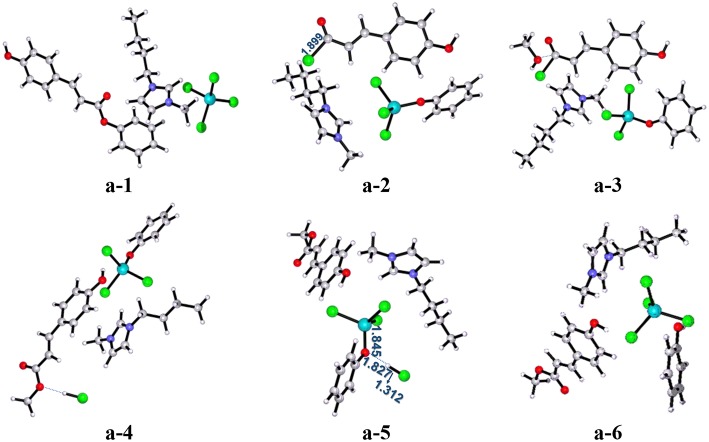
B3LYP-D3/BSI optimized geometries of key stationary points corresponding to pathway **a**. Distances are given in angstroms.

As shown in Scheme 3 (pathway **a**), acylation chlorination mechanism from **PPC** to **MPC** catalyzed by [Bmim][FeCl_4_] is formed via three elementary steps: acyl chlorination, alcoholysis, and catalyst recovery. A detailed catalysis process corresponding to pathway **a** is also shown in Scheme S1. Figure 1 displays the calculated energy profile (*G*_s_) with the optimized geometries of transition states corresponding to pathway **a**, and those of reactants, intermediates, and products are given in Figure 2. The reactants (**PPC** and methanol) with an ionic pair of [Bmim][FeCl_4_] is taken as the zero reference point of relative energy in the following discussion. A clearer picture of all optimized structures of stationary points corresponding to pathway **a** is shown in Figure S1 and IRC calculations starting from **a-1** to **a-2, a-3** to **a-4**, and **a-5** to **a-6** are given in Figures S2–S4.

In **a-1**, the reaction starts with the nucleophilic attack of chlorine anion of [FeCl_4_]^−^ at the C=O group in the lignin model compound **PPC**, forming intermediate **a-2** included acyl chloride compound and [FeCl_3_OPh]^−^, as described by transition state **a-ts1**. In this step, the [FeCl_4_]^−^ anion of [Bmim][FeCl_4_] acts as a nucleophile and C-O ester group of **PPC** serves as an electrophile to introduce chlorine anion, generating intermediate **a-2**. It is noted that the phenoxy group in **PPC** has a strong electron-donating ability, which leads to the activation of the C-O group with a bond distance of 1.970 Å and promotes the reaction. This nucleophilic attack process involves a free energy barrier of 37.1 kcal/mol (**a-ts1** relative to **a-1**). Once chlorine anion is introduced, the strong polarity of acyl chloride group in intermediate **a-2** makes it easy to be replaced by other groups. Therefore, when methanol enters into the reaction system to attack acyl chloride, methoxy substitution is easy to occur with the elongation of C-Cl bond distance (from 1.899 to 2.573 Å) and the conversion of intermediate **a-3** to **a-4** is realized by alcoholysis under the catalysis of [Bmim][FeCl_4_]. It results in the formation of the product **MPC** through **a-ts2** involving a relative free energy barrier of 13.3 kcal/mol (**a-ts2** relative to **a-3**). The followed is the regeneration of the catalyst. HCl first approaches the phenoxy group and then forms another intermediate **a-5**. With the increase of H-Cl (from 1.312 to 1.683 Å) and Fe-O (from 1.845 to 1.985 Å) bond lengths and the decrease of O-H (from 1.827 to 1.103 Å) bond length, the catalyst is finally regenerated through **a-ts3** with an energy barrier of 2.0 kcal/mol (**a-ts3** relative to **a-5**) and releases 3.4 kcal/mol of energy.

By contrast to the energy barriers of the alcoholysis and catalyst regeneration steps only at 13.3 and 2.0 kcal/mol, the acylation process of that as high as 37.1 kcal/mol is determined as the rate-limiting step by the cleavage of the γ-O ester linkage in **PPC**. In this pathway, the -COOR functional group in **PPC** was first modified to -COCl by acyl chlorination reaction and then followed by the addition of methanol through an alcoholysis reaction to obtain the **MPC** product. This process is supported by Nese and Aysen's work (Kaynak et al., 2018) that -COCl is a very reactive functional group and can easily be transformed to -COOR functionality with a lower energy barrier. By a quantitative analysis of the mechanism of acylation chlorination, it is found that the high energy barrier indicates the formation of -COCl may be related to the steric hindrance of substrates and the coordination effect of Fe atom (Cunico and Pandey, 2005; Maslivetc et al., 2018).

#### Pathway b–Synergistic Effect of Cation and Anion

In pathway **a**, the initiation of the reaction begins with the attack of Cl atom of [FeCl_4_]^−^ at C=O group of **PPC**. Alternatively, Fe atom of [FeCl_4_]^−^ can also interact with the O atom of the C=O group because of the coordination effect of the iron atom. Consequently, the second possible pathway denoted as pathway **b** is proposed and shown in Scheme 3. A detailed catalysis process corresponding to pathway **b** is also shown in Scheme S2. A clearer picture of all optimized structures of stationary points corresponding to pathway **b** is shown in Figure S5 and IRC calculations starting from **b-1** to **b-2, b-3** to **b-4**, and **b-5** to **P**+cat. are given in Figures S6–S8.

**Figure 3:**
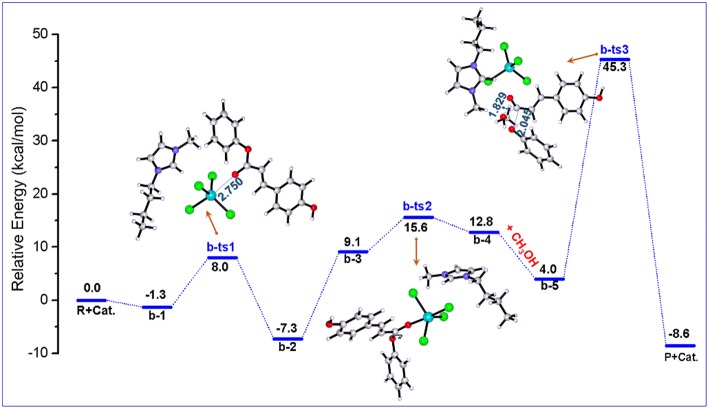
Calculated Gibbs free energy (*G*_s_) profile with optimized geometries of transition states, corresponding to pathway **b** shown in Scheme 3. Distances are given in angstroms.

**Figure 4:**
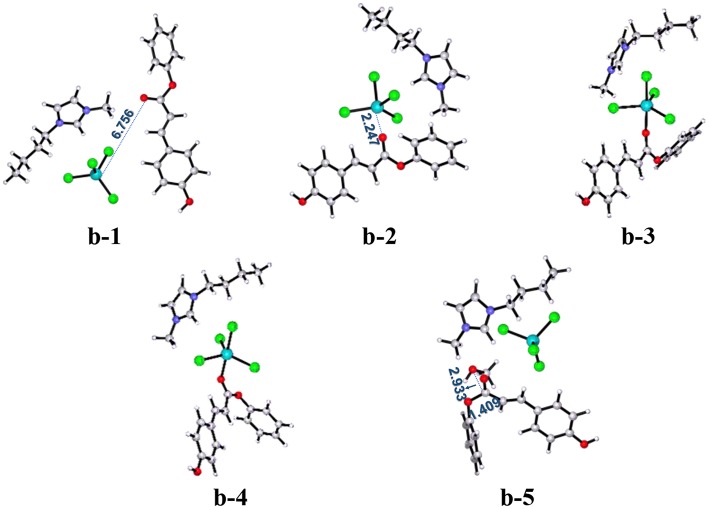
B3LYP-D3/BSI optimized geometries of key stationary points corresponding to pathway **b**. Distances are given in angstroms.

Figure 3 illustrates the calculated free energy (G_*s*_) profile with the optimized geometries of transition states corresponding to pathway **b**, and those of reactants, intermediates, and products are given in Figure 4. The reactants (**PPC** and methanol) with an ionic pair of [Bmim][FeCl_4_] is taken as the zero-reference point of relative energy in this pathway. When isolated reactant **PPC** and [Bmim][FeCl_4_] catalyst is close to each other, **b-2** complex is first formed through transition state **b-ts1** with a relative energy barrier of 9.3 kcal/mol. In this step, the transition state **b-ts1** is characterized by the coordination interaction between Fe atom of [FeCl_4_]^−^ and O atom of C=O group of **PPC** with shortened O-Fe bond distance (2.750 Å), suggesting the activation of **PPC** molecule. From **b-2**, the intermediate **b-3** is formed via adjusting the position of cation to the upper region of anion to reduce the steric hindrance between **PPC** molecule and the cation of [Bmim][FeCl_4_] catalyst and make next reaction step more easily occur. Based on **b-3**, the position of [Bmim][FeCl_4_] and the angle of ester C-O bond in **PPC** start to change via **b-ts2** to form intermediate **b-4** with a relative energy barrier of 6.5 kcal/mol. Although this step seems simple enough, the strong steric hindrance might make this step with a high energy barrier. After the position of the ester C-O bond is adjusted, another intermediate **b-5** containing methanol is formed. From **b-5**, the transition state **b-ts3** can be generated by the deprotonation of methanol, and finally the product **MPC** is produced by transesterification with a relative energy barrier of 41.3 kcal/mol (overall energy barrier is 52.6 kcal/mol, **b-ts3** relative to **b-2**). At the same time, the catalyst is regenerated, making the completion of the whole catalytic cycle.

According to the above calculation results, it can be found that the coordination between Fe atom of [FeCl_4_]^−^ and O atom of C=O group leads to the activation of **PPC**, and the cation also promotes the coordination process by adjusting its position. In other words, the synergistic effects of the cation and anion make it easier for Fe atom of [Bmim][FeCl_4_] to attack the C=O group on **PPC**. Also, according to the literature (Lu et al., 2016), structural rearrangement is also beneficial to the reduction of the energy barrier. Therefore, by rotating the C-O bond of the phenoxy group, the intermediate **b-3** readjusts the position of the large group, which is more conducive to the attack of methanol, thus forming the intermediate **b-4**. This intermediate is geometrically ready to carry out the transesterification through transition state **b-ts3** with the addition of methanol. After that, the gradually moving away of phenoxy group from **PPC** results in the break of the ester C-O bond. The evolution is magnified by the elongated C-O bond of phenoxy group and the shortened C-O bond of methanol, 2.045 and 1.829 Å (in **b-ts3)**, respectively. The forward minimum from **b-ts3** can produce the final product **MPC**, realizing the complete of this catalytic cycle. As shown in Figure 3, although the first several steps of the reaction proceed smoothly due to the synergistic effect of cations and anions of ionic liquid catalyst, the energy barrier of the last step is as high as 52.6 kcal/mol, which makes the whole pathway infeasible and needs to be excluded.

#### Pathway c–Acid Catalysis Process

**Figure 5:**
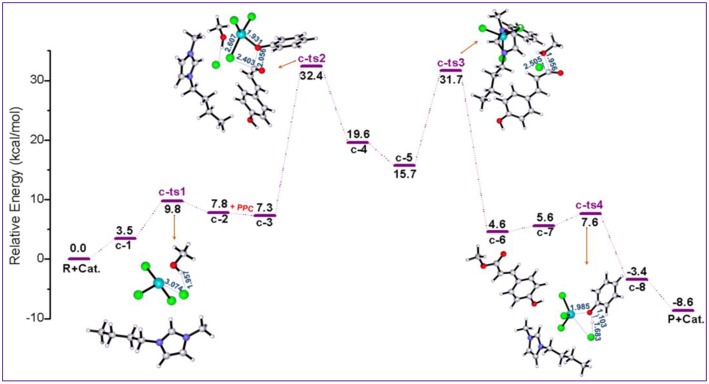
Calculated Gibbs free energy (*G*_s_) profile with optimized geometries of transition states, corresponding to pathway **c** shown in Scheme 3. Distances are given in angstroms.

**Figure 6:**
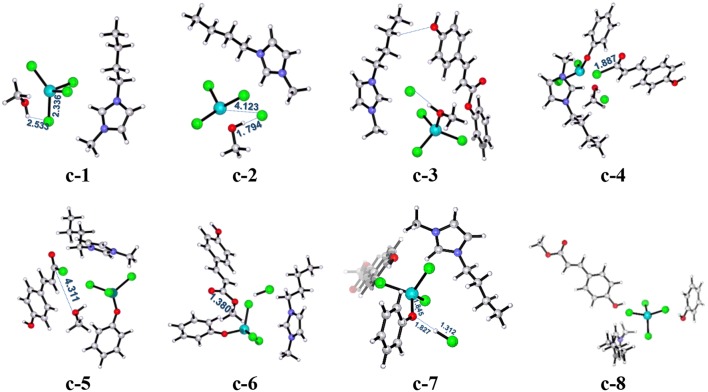
B3LYP-D3/BSI optimized geometries of key stationary points corresponding to pathway **c**. Distances are given in angstroms.

All the above two pathways start with the activation of **PPC** by the catalyst. By contrast, we further proposed another reaction route in Scheme 3 denoted as pathway **c**, where the catalyst first interacts with methanol molecule and contains four stages: dechlorination, Fries-like rearrangement, alcoholysis, and catalyst recovery. A detailed catalysis process corresponding to pathway **c** is also illustrated in Scheme S3. The calculated free energy profiles (*G*_s_) with schematic geometries along the reaction coordinate are shown in Figures 5, 6. A clearer picture of all optimized structures of stationary points corresponding to pathway **c** is shown in Figure S9 and IRC calculations starting from **c-1** to **c-2**, **c-3** to **c-4**, **c-5** to **c-6**, and **c-7** to **c-8** are given in Figures S10–S13.

The ionic liquid catalyst first interacts with methanol molecule through forming hydrogen bond (O-H^…^Cl, 2.533 Å) as illustrated in Figure 6 denoted as intermediate **c-1**. Subsequently, intermediate **c-1** converts into intermediate **c-2** through transition state **c-ts1** with a relative energy barrier of 6.3 kcal/mol, suggesting that this interaction is feasible. In this step, the imaginary vibration of **c-ts1** corresponds to the shrinking of H-Cl bond (from 2.533 to 1.794 Å) and stretching of Fe-Cl bond (from 2.336 to 4.123 Å), which indicates that the transfer of Cl^−^ anion from [FeCl_4_]^−^ anion to H atom of -OH group in methanol and the dechlorinating stage is completed, thus forming -FeCl_3_ with a concerted mechanism. Then, **PPC** participates in the reaction process, and complex **c-3** is formed via hydrogen bond between H atom on the alkyl chain in the cation and O atom on the phenol group in **PPC**. Following the formation of **c-3**, the attack of -FeCl_3_ to O atom on C-O bond in **PPC** results in the C-O cleavage process via transition state **c-ts2**. The relative free energy barrier for this step is calculated to be 25.1 kcal/mol (overall energy barrier is 32.4 kcal/mol), implying that the cleavage of C-O bond in such a way is more feasible than pathways **a** and **b** under 420 K with certain pressure. The process may be explained by the Fries-like rearrangement (Ucar et al., 1998; Guenadil and Aichaoui, 2003), which leads to the formation of complex **c-4** containing an acyl chloride group. Moreover, this step is also the rate-determining step of the reaction. After the dissociation of Fe-Cl bond in the complex **c-2**, free Cl^−^ anion is formed, which further attacks complex **c-4** with the help of methanol, resulting in the formation of the complex **c-5** with an acyl chloride group. From **c-5**, the acyl chloride complex reacts with methanol to produce the final product **MPC** by alcoholysis via **c-ts3** with a relative energy barrier of 16.0 kcal/mol. The final step is similar to the process corresponding to pathway **a**, which completes the regeneration of the catalyst with a relative energy barrier of 2.0 kcal/mol and releases 3.4 kcal/mol of energy, also indicating the end of the reaction.

The above mechanism calculation corresponding to pathway **c** shows that the conversion of **c-1** to **c-2** by the production of -FeCl_3_ and free Cl^−^ anion is a very critical step for the whole reaction. The production of Lewis acid, -FeCl_3_, can promote the occurrence of Fries-like rearrangement reaction. The specific steps of this Fries-like rearrangement reaction are revealed as follows: complex **c-3** forms typical intermediate **c-4** as expected through an intermolecular rearrangement reaction, which emphasizes the dissociation of the phenoxy group. However, the completion of transesterification cannot be determined by the above steps. The subsequent acyl chloride complex **c-5** completes the transfer of alkoxy group through the transition state **c-ts3** and obtains the target product **MPC**. In this process, when only -FeCl_3_ or [Bmim][Cl] catalyst is used, it would fail to obtain complex **c-5** containing phenoxy ion and acyl chloride group, although the depolymerization of lignin is also an acid catalytic process, which means the complex **c-5** is a crucial intermediate to produce **MPC**. Therefore, [Bmim][FeCl_4_] is a useful medium for this improved acid catalytic process. It can act as both a Lewis acid catalyst for providing -FeCl_3_ and a chlorination reagent for providing free Cl^−^ anion.

### Further Discussion

Based on the above studies, we concluded that the activation of C-O ester bond is the key to produce the target **MPC** product and the reaction might proceed by three possible pathways with different probability, depending on their energy barriers. Among them, pathways **a** and **c** need to be converted to the product through -COCl, while pathway **b** can be achieved by the direct cleavage of the ester C-O bond. Besides, our proposed mechanism can rationally explain to a certain extent that the cleavage of -COCl (pathways **c**) is more accessible than direct cleavage of ester C-O bond. The fact is that the acyl chloride complex **c-5** has better reactivity than other intermediates (Qu et al., 2015). The most obvious advantage of this pathway is that it is a nucleophilic catalytic process. The substitution of Cl for phenoxy moiety to activate carbonyl is beneficial to the consequent attack by methanol molecule.

The overall energy barriers of the three pathways are calculated to be 37.1, 52.6, and 32.4 kcal/mol, respectively (see Figure S14), indicating that the pathway **c** is the energetically most favorable process than the other two for lignin depolymerization. These fairly high energy barriers also mean that the cleavage of C-O ether bond in the lignin is not smooth. A similar result was reported previously by Zhang's group (Jing et al., 2019). The results also demonstrate that pathway **c** has much lower activation energy in the initial step, revealing that pathway **c** may be the most optimal process. It can be said that pathway **c** integrates all the advantages of the other two pathways to some extent, but the effect of each elementary step is different from the previous mechanism. For example, carbocation intermediate can be obtained by using the Lewis acid -FeCl_3_ produced by [FeCl_4_]^−^ anion to complete the Fries-like rearrangement. At the same time, the free Cl^−^ anion is more favorable to the acyl chloride reaction compared to [FeCl_4_]^−^. Therefore, [Bmim][FeCl_4_] is an efficient catalyst that combines the advantages of acid catalysis and acyl chlorination.

### Solvation Effect

**Figure 7:**
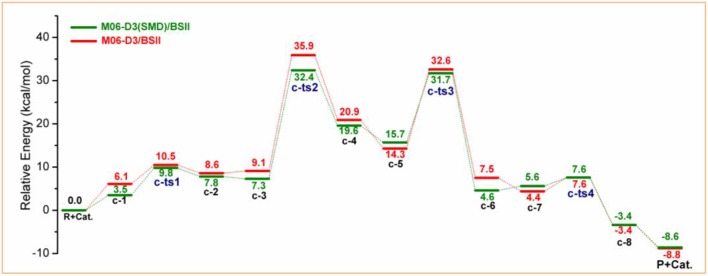
Calculated Gibbs free energy profiles corresponding to pathway **c** in the gas phase (red) and with the SMD model (green).

The above calculations have provided a mechanism understanding of the depolymerization of lignin model compound **PPC** catalyzed by [Bmim][FeCl_4_], and the catalytic properties of MBILs were explained to a certain extent. According to previous studies (Sánchez-Sanz and Trujillo, 2018; Zhang et al., 2019), the solvent effect is of great importance for biomass conversion due to their impacts on the whole reaction. Figure 7 shows the relative Gibbs free energies of reactants, transition states, intermediates, and products corresponding to the reaction pathway **c**, which is the most energetically favorable path, in the gas phase and with solvation model. The Gibbs free energy profiles corresponding to the reaction pathways **a** and **b** both in the gas phase and with solvation model are shown in Figures S15, S16. Moreover, the specific energy barriers corresponding to the transition states are summarized in Table 1.

**Table 1:** The energy barriers for pathway **c** in the gas phase and with the SMD model.

**Transition state**	**Energy barrier (kcal/mol)**		
	**Gas phase**	**Methanol (SMD)**	**ΔΔG**
c-ts1	10.5	9.8	0.7
c-ts2	35.9	32.4	3.5
c-ts3	32.6	31.7	0.9
c-ts4	7.6	7.6	0.0

It is noted that the solvent effect hardly influences the reaction paths but the relative energies. The energy barriers associated with **c-ts1**, **c-ts2**, **c-ts3**, and **c-ts4** are 9.8, 32.4, 31.7, and 7.6 kcal/mol with the SMD model, and 10.5, 35.9, 32.6, and 7.6 kcal/mol in the gas phase, respectively (see Table 1). There is a slight solvent effect for the first three steps, **c-ts1**, **c-ts2**, and **c-ts3**, where the energy barriers are reduced by 0.7, 3.5, and 0.9 kcal/mol, respectively, while the last step **c-ts4** has almost no difference, which may be ascribed to the different solvation behavior of methanol to the intermediates and transition states. On the one hand, methanol molecules can be either a hydrogen-bond donor or a hydrogen-bond acceptor (He et al., 2013), which will increase the probability of methanol to form hydrogen bond with [FeCl_4_]^−^, as well as increase the hydrogen-bonding interactions between anions and methanol, thus the energy barrier to form **c-2** is decreased. On the other hand, the solvation effect of implicit methanol was found to induce a polarization of the transition structure and enhance the electron-donating ability (Domingo et al., 1999; Kong and Evanseck, 2000; Domingo and Andres, 2003), and the protic solvent is beneficial for proton transfer in **c-ts2** and **c-ts3**, which can lower the energy barriers of **c-ts2** and **c-ts3**. Additionally, methanol molecules can solvate **c-3** to form **c-4** (Fries-like rearrangement step), leading to lower energy barrier of **c-ts2**. Note that the Fries-like rearrangement is the essential step in **PPC** catalysis (Pitchumani et al., 1996), and the reaction energy barrier can be reduced to a certain extent in the methanol solvent environment, which illustrates that methanol has a suitable solvent effect for catalyzing **PPC**.

In fact, methanol can serve as both reactant and solvent. It undergoes a nucleophilic attack to form an intermediate, then proton transfer and transesterification are carried out with breaking the bond of the intermediate. In this regard, the reaction can be promoted in this protic solvent. However, the amount of solvent should be controlled in practical application.

## Conclusion

In summary, by performing DFT calculations, we systematically investigated the mechanism of MBIL-catalyzed cleavage of γ-O ester linkage of the lignin model **PPC** to **MPC**. Three reaction pathways, including acyl chlorination process (pathway **a**), transesterification processes (pathway **b**), and Lewis acid catalyzed conversion (pathway **c**) were proposed, and the [FeCl_4_]^−^ anion of catalyst is proved to play an essential role in activating **PPC** or methanol molecule to promote the reaction. It is found that the overall energy barrier of pathway **c** is much lower than that of pathways **a** and **b**, indicating that Lewis acid catalysis is an energetically more favorable process with the most probability. This path takes the advantages of the enhanced polarity by acyl chlorination and Fries-like rearrangement by Lewis acid, leading to lower energy barriers in the initial and the rate-determining steps. Additionally, methanol serves as both solvent and reactant, which contributes to this reaction. Overall, these results suggest that MBIL could be an effective catalyst for lignin valorization, and we hope that this work will provide more profound insights for current studies and inspire further development of efficient catalysts for biomass transformation.

## Data Availability

All datasets generated for this study are included in the manuscript and/or the Supplementary Files.

## Author Contributions

HH and XL designed the research. TZ, YZ, YW, and FH carried out the whole simulation. TZ, YZ, ZL, and HH performed data analysis. HH, XL, TZ, YZ, and QZ wrote the manuscript.

### Conflict of Interest Statement

The authors declare that the research was conducted in the absence of any commercial or financial relationships that could be construed as a potential conflict of interest.
