# Supplementary material for: Theoretical Insights Into the Depolymerization Mechanism of Lignin to Methyl p-hydroxycinnamate by [Bmim][FeCl4] Ionic Liquid
Source: Front Chem. 2019 Jun 18;7:446. doi: 10.3389/fchem.2019.00446 (PMC6591258; doi:10.3389/fchem.2019.00446)
Supplement: Supplementary file 1 [file Data_Sheet_1.docx]

Supporting Information

**Theoretical insights into the depolymerization mechanism of lignin to methyl *p*-hydroxycinnamate by [Bmim][FeCl_4_] ionic liquid**

Tian Zhang,^a,b^ Yaqin Zhang,^b^ Yanlei Wang,^b^ Feng Huo,^b^ Zhangmin Li,^a^ Qiang Zeng,^a^

Hongyan He,^b*^ Xuehui Li^a*^

^a^*School of Chemistry and Chemical Engineering, State Key Laboratory of Pulp and Paper Engineering, South China University of Technology, Guangzhou 510640, China. E-mail: cexhli@scut.edu.cn*

^b^*Beijing Key Laboratory of Ionic Liquids Clean Process, CAS Key Laboratory of Green Process and Engineering, State Key Laboratory of Multiphase Complex Systems, Institute of Process Engineering, Chinese Academy of Sciences, Beijing 100190, China. Email: hyhe@ipe.ac.cn*

1. Key Optimized Geometries and IRC Calculations (pages s2-s6)
2. Schematic Diagram of the Three Potential Catalytic Mechanisms (pages s7-s8)
3. Calculated Gibbs Free Energy Profiles along Pathways **a**, **b**, and **c** (page s9)
4. Gibbs Free Energy Profiles in Gas Phase and with Solvation Model Corresponding to Pathways **a** and **b** (page s10)
5. Energies (Gas Phase and Solvent Correction) and Cartesian Coordinates of All the Structures (pages s11-s109)

# Key Optimized Geometries and IRC Calculations

| **** | **** | **** |
| --- | --- | --- |
| **a-1** | **a-ts1** (198.64*i* cm^-1^) | **a-2** |
| **** | **** | **** |
| **a-3** | **a-ts2** (150.62*i* cm^-1^) | **a-4** |
| **** | **** | **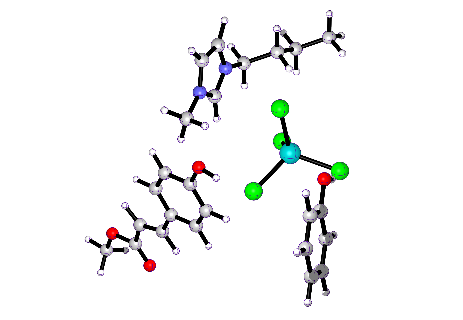** |
| **a-5** | **a-ts3** (276.09*i* cm^-1^) | **a-6** |

**Fig. S1** Key optimized geometries of stationary points labeled in Fig. 1. Bond lengths are in angstroms. Values in brackets are imaginary frequencies for transition states at the B3LYP-D3/BSI level.

**
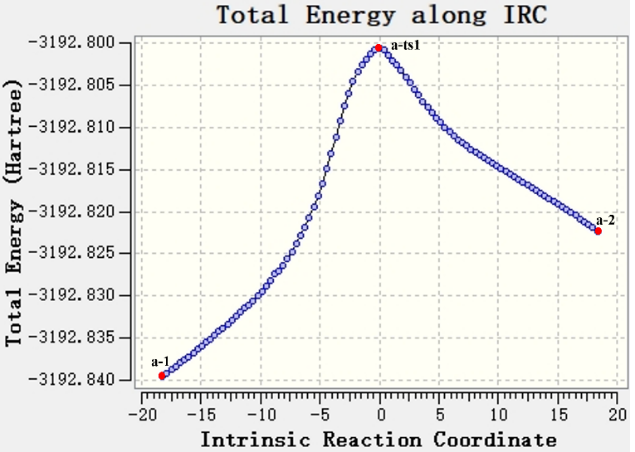
**

**Fig. S2** IRC calculation (B3LYP-D3/BSI level) starting from **a-1**, leading to **a-2**.

**
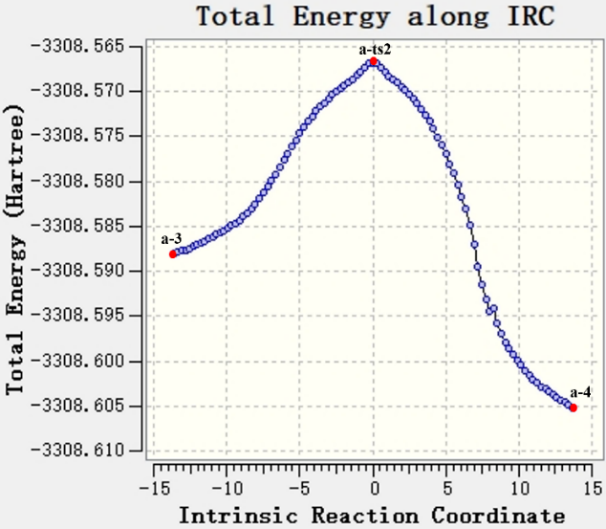
**

**Fig. S3** IRC calculation (B3LYP-D3/BSI level) starting from **a-3**, leading to **a-4**.

**
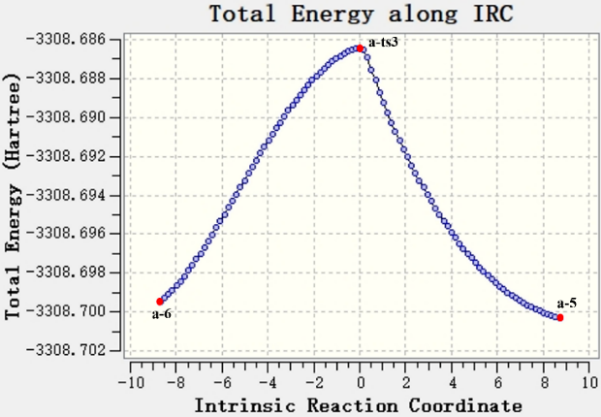
**

**Fig. S4**  IRC calculation (B3LYP-D3/BSI level) starting from **a-5**, leading to **a-6**.

| **** | **** | **** |
| --- | --- | --- |
| **b-1** | **b-ts1** (22.51*i* cm^-1^) | **b-2** |
| **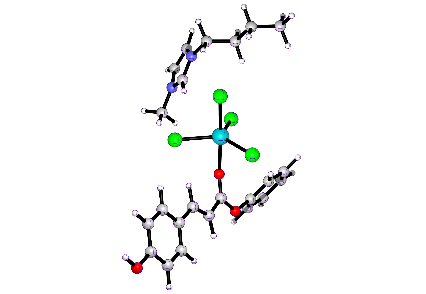** | **** | **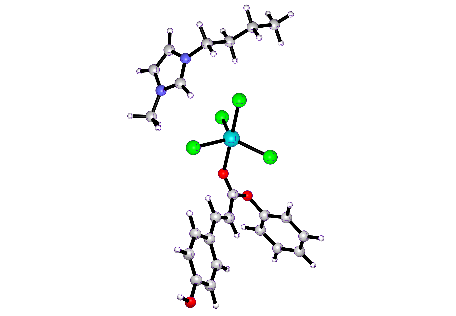** |
| **b-3** | **b-ts2** (47.30*i* cm^-1^) | **b-4** |
| **** | **** | **** |
| **b-5** | **b-ts3** (402.08*i* cm^-1^) | **P+cat.** |

**Fig. S5** Key optimized geometries of stationary points labeled in Fig. 3. Bond lengths are in angstroms. Values in brackets are imaginary frequencies for transition states at the B3LYP-D3/BSI level.

**Fig. S6** IRC calculation (B3LYP-D3/BSI level) starting from **b-1**, leading to **b-2**.


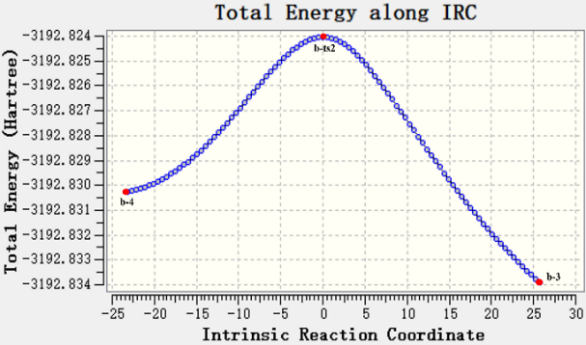


**Fig. S7** IRC calculation (B3LYP-D3/BSI level) starting from **b-3**, leading to **b-4**.

**Fig. S8**  IRC calculation (B3LYP-D3/BSI level) starting from **b-5**, leading to P+cat.

| **** | **** | **** |
| --- | --- | --- |
| **c-1** | **c-ts1** (81.51*i* cm^-1^) | **c-2** |
| **** | **** | **** |
| **c-3** | **c-ts2** (194.35*i* cm^-1^) | **c-4** |
| **** | **** | **** |
| **c-5** | **c-ts3** (210.48*i* cm^-1^) | **c-6** |
| **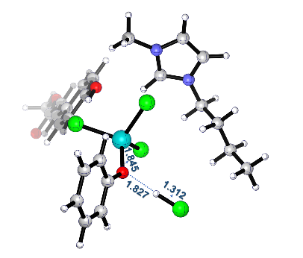** | **** | **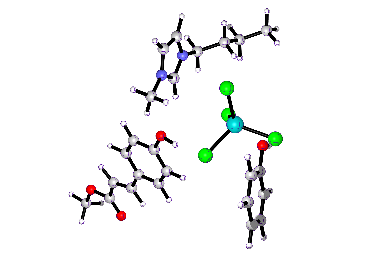** |
| **c-7** | **c-ts4** (276.09*i* cm^-1^) | **c-8** |

**Fig. S9** Key optimized geometries of stationary points labeled in Fig. 5. Bond lengths are in angstroms. Values in brackets are imaginary frequencies for transition states at the B3LYP-D3/BSI level.

**
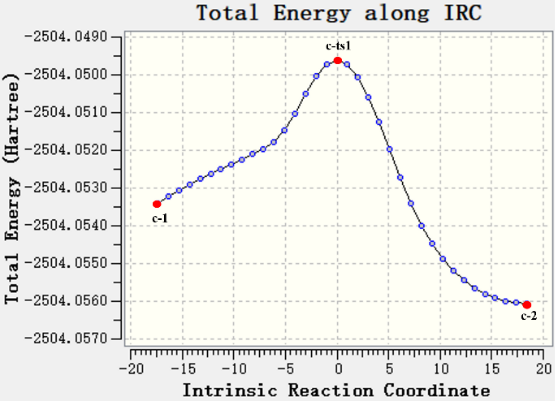
**

**Fig. S10** IRC calculation (B3LYP-D3/BSI level) starting from **c-1**, leading to **c-2**.

**
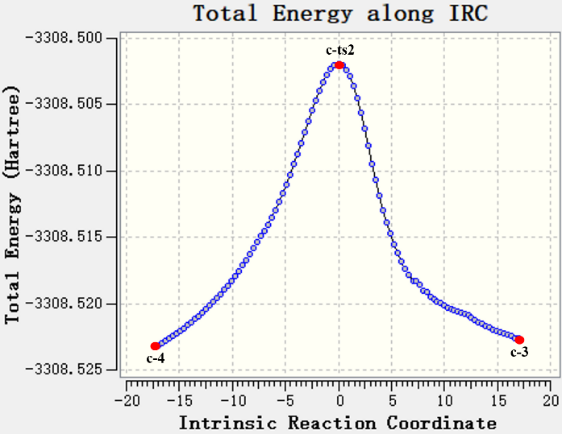
**

**Fig. S11** IRC calculation (B3LYP-D3/BSI level) starting from **c-3**, leading to **c-4**.

**
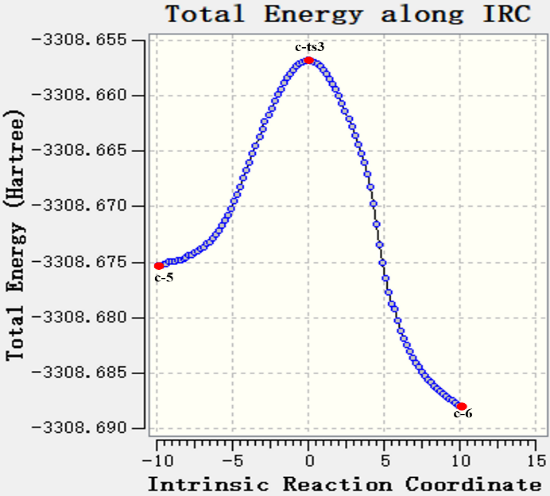
**

**Fig. S12** IRC calculation (B3LYP-D3/BSI level) starting from **c-5**, leading to **c-6**.


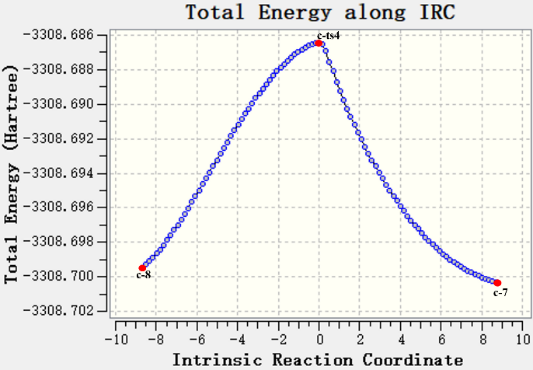


**Fig. S13** IRC calculation (B3LYP-D3/BSI level) starting from **c-7**, leading to **c-8**.

# Schematic Diagram of the Three Potential Catalytic Mechanisms

**Scheme S1** Acylation chlorination mechanism from **PPC** to **MPC** indicated by pathway **a**.

**Scheme S2** Transesterification mechanism from **PPC** to **MPC** indicated by pathway **b**.

**Scheme S3** Transesterification mechanism from **PPC** to **MPC** indicated by pathway **c**.


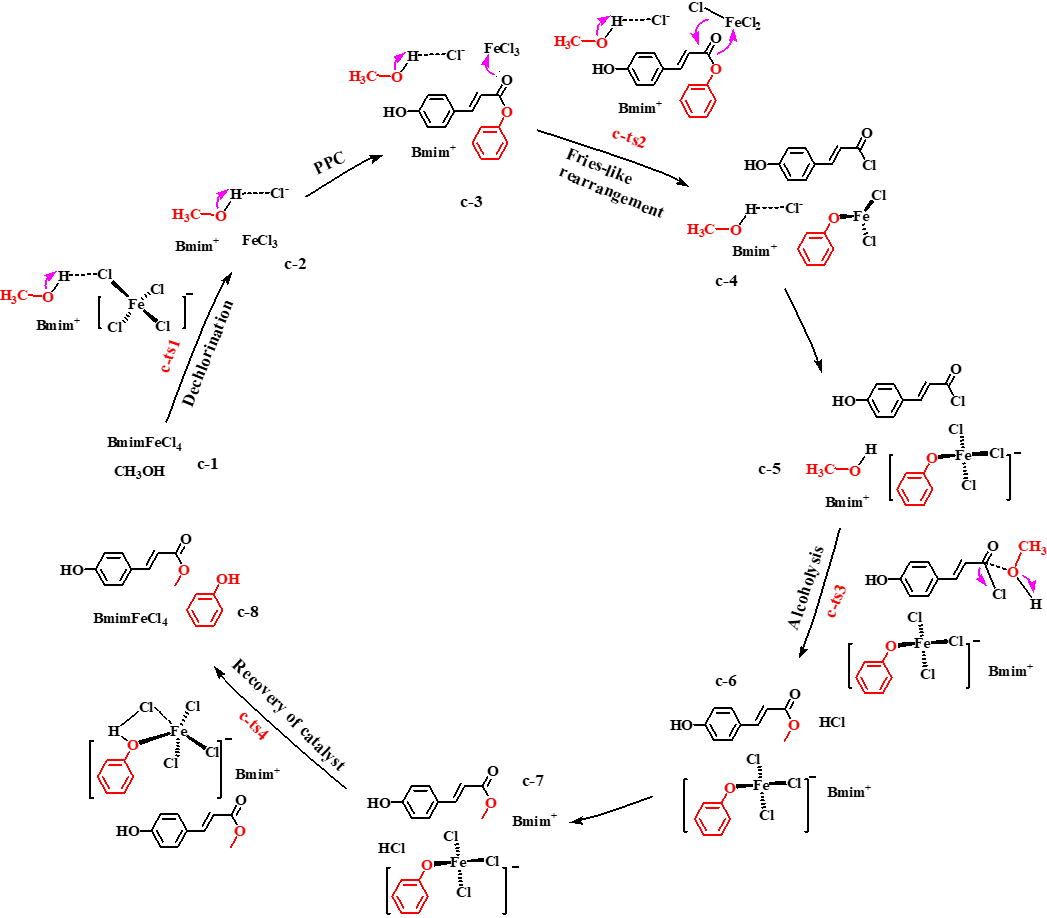


# Calculated Gibbs Free Energy Profiles along Pathways a, b, and c

**Fig. S14** Calculated Gibbs free energy profile (*G*_s_) along pathways **a**, **b**, and **c**.

# Gibbs Free Energy Profiles in Gas Phase and with Solvation Model Corresponding to Pathways a and b

|  |
| --- |
| **Fig. S15** Calculated Gibbs free energy profile corresponding to pathway **a** in gas phase  and with solvation model. |
|  |
| **Fig. S16** Calculated Gibbs free energy profile corresponding to pathway **b** in gas phase  and with solvation model. |

# Energies and Cartesian Coordinates of All the Structures

All the following coordinates and energies were obtained at the level of M06-D3/BSII and M06-D3(SMD)/BSII, and energies are given in Hartree.

**CH_3_OH**

SCF energy in gas phase: -115.687993257 a.u.

C 0.66165400 -0.01943500 0.00000000

H 1.08221900 0.98943100 -0.00000100

H 1.03828200 -0.54290700 -0.89182200

H 1.03828200 -0.54290500 0.89182300

O -0.74937800 0.12204300 0.00000000

H -1.13368200 -0.76335500 0.00000000

**PPC**

SCF energy in gas phase: -804.149235467 a.u.

C 4.49531500 -1.30999700 0.31714200

C 3.14472600 -1.01131700 0.24933600

C 2.69339800 0.28700600 -0.07085800

C 3.66603300 1.27155700 -0.31989800

C 5.02551000 0.98567400 -0.25547000

C 5.44638600 -0.30955400 0.06412800

H 4.84086700 -2.30821300 0.56344400

H 2.42453700 -1.79841100 0.44832700

H 3.34663900 2.27978100 -0.56877300

H 5.75858700 1.76464800 -0.45247900

O 6.75855900 -0.66240800 0.14543900

H 7.31064900 0.10698000 -0.04839800

C 1.28501300 0.65109800 -0.15408000

H 1.07530800 1.68900700 -0.40884500

C 0.20985700 -0.13756900 0.04474500

H 0.28131800 -1.18852500 0.30190800

C -1.14340600 0.42216000 -0.08940800

O -1.42536200 1.56858700 -0.36884000

O -2.06964300 -0.56730800 0.14971000

C -3.43940100 -0.32373500 0.08677300

C -4.04244500 0.82533800 0.60179700

C -4.20477700 -1.35592200 -0.45660000

C -5.43341600 0.93042400 0.55570000

H -3.43780300 1.62111400 1.01416200

C -5.59309100 -1.23715200 -0.49204100

H -3.70115300 -2.23699200 -0.84013900

C -6.21244400 -0.09217100 0.01221400

H -5.90854800 1.82331400 0.95133400

H -6.18875400 -2.04083000 -0.91465300

H -7.29362600 0.00211600 -0.01722700

**[Bmim][FeCl_4_]**

SCF energy in gas phase: -2388.11562500 a.u.

C -2.04069900 2.99913900 0.78215400

C -2.88852400 1.96807500 0.50061800

C -1.06231200 1.70196700 -0.71607000

N -0.91325100 2.82262900 0.00349500

H -2.13876900 3.83122700 1.46055400

H -3.86775600 1.73191000 0.88392200

H -0.31660600 1.27225100 -1.36959300

N -2.26226100 1.17526500 -0.44259600

C 0.32335800 3.60524700 0.11320300

H 0.82456900 3.62850500 -0.85251600

H 0.06388900 4.61673100 0.42794400

H 0.97840800 3.12275500 0.84181200

C -2.76875000 -0.11920400 -0.94092800

H -3.77545800 0.05149100 -1.33571800

H -2.11679900 -0.41749500 -1.76320400

C -2.75824900 -1.18208600 0.16115100

H -1.75556400 -1.21637300 0.59821100

H -3.45258800 -0.89079800 0.96119500

C -3.13765700 -2.56277100 -0.38641000

H -4.13613600 -2.52167400 -0.84498000

H -2.43084000 -2.83154300 -1.18091600

C -3.11031100 -3.63913400 0.70457300

H -3.81612900 -3.40622600 1.51099900

H -3.37669200 -4.62020400 0.29821600

H -2.11044500 -3.71771900 1.14502100

Fe 1.73345200 -0.49819200 0.03539800

Cl 0.47710800 0.30263200 1.72836500

Cl 2.45970500 1.27297500 -1.13848200

Cl 3.36402000 -1.75714100 0.77082500

Cl 0.36061600 -1.60037900 -1.35702200

**CH_3_OH**

SCF energy in solvent model (SMD): -115.697345750 a.u.

C 0.66165400 -0.01943500 0.00000000

H 1.08221900 0.98943100 -0.00000100

H 1.03828200 -0.54290700 -0.89182200

H 1.03828200 -0.54290500 0.89182300

O -0.74937800 0.12204300 0.00000000

H -1.13368200 -0.76335500 0.00000000

**PPC**

SCF energy in solvent model (SMD): -804.173768608 a.u.

C 4.49531500 -1.30999700 0.31714200

C 3.14472600 -1.01131700 0.24933600

C 2.69339800 0.28700600 -0.07085800

C 3.66603300 1.27155700 -0.31989800

C 5.02551000 0.98567400 -0.25547000

C 5.44638600 -0.30955400 0.06412800

H 4.84086700 -2.30821300 0.56344400

H 2.42453700 -1.79841100 0.44832700

H 3.34663900 2.27978100 -0.56877300

H 5.75858700 1.76464800 -0.45247900

O 6.75855900 -0.66240800 0.14543900

H 7.31064900 0.10698000 -0.04839800

C 1.28501300 0.65109800 -0.15408000

H 1.07530800 1.68900700 -0.40884500

C 0.20985700 -0.13756900 0.04474500

H 0.28131800 -1.18852500 0.30190800

C -1.14340600 0.42216000 -0.08940800

O -1.42536200 1.56858700 -0.36884000

O -2.06964300 -0.56730800 0.14971000

C -3.43940100 -0.32373500 0.08677300

C -4.04244500 0.82533800 0.60179700

C -4.20477700 -1.35592200 -0.45660000

C -5.43341600 0.93042400 0.55570000

H -3.43780300 1.62111400 1.01416200

C -5.59309100 -1.23715200 -0.49204100

H -3.70115300 -2.23699200 -0.84013900

C -6.21244400 -0.09217100 0.01221400

H -5.90854800 1.82331400 0.95133400

H -6.18875400 -2.04083000 -0.91465300

H -7.29362600 0.00211600 -0.01722700

**[Bmim][FeCl_4_]**

SCF energy in solvent model (SMD): -2388.15589947 a.u.

C -2.04069900 2.99913900 0.78215400

C -2.88852400 1.96807500 0.50061800

C -1.06231200 1.70196700 -0.71607000

N -0.91325100 2.82262900 0.00349500

H -2.13876900 3.83122700 1.46055400

H -3.86775600 1.73191000 0.88392200

H -0.31660600 1.27225100 -1.36959300

N -2.26226100 1.17526500 -0.44259600

C 0.32335800 3.60524700 0.11320300

H 0.82456900 3.62850500 -0.85251600

H 0.06388900 4.61673100 0.42794400

H 0.97840800 3.12275500 0.84181200

C -2.76875000 -0.11920400 -0.94092800

H -3.77545800 0.05149100 -1.33571800

H -2.11679900 -0.41749500 -1.76320400

C -2.75824900 -1.18208600 0.16115100

H -1.75556400 -1.21637300 0.59821100

H -3.45258800 -0.89079800 0.96119500

C -3.13765700 -2.56277100 -0.38641000

H -4.13613600 -2.52167400 -0.84498000

H -2.43084000 -2.83154300 -1.18091600

C -3.11031100 -3.63913400 0.70457300

H -3.81612900 -3.40622600 1.51099900

H -3.37669200 -4.62020400 0.29821600

H -2.11044500 -3.71771900 1.14502100

Fe 1.73345200 -0.49819200 0.03539800

Cl 0.47710800 0.30263200 1.72836500

Cl 2.45970500 1.27297500 -1.13848200

Cl 3.36402000 -1.75714100 0.77082500

Cl 0.36061600 -1.60037900 -1.35702200

**a-1**

SCF energy in gas phase: -3192.28723969 a.u.

Fe 5.15651700 0.37682400 -0.85204400

Cl 5.34561500 1.98521500 0.73351300

Cl 3.25186200 0.80796900 -1.97079200

Cl 6.90141400 0.34978400 -2.18316600

Cl 4.88538400 -1.57687000 0.22194200

C -8.91748500 -0.48632200 -1.04693200

C -7.57616900 -0.78281600 -0.87584300

C -6.72875400 0.05629800 -0.11914600

C -7.29513500 1.20830700 0.45714500

C -8.64053800 1.51803900 0.29333200

C -9.45949800 0.66974400 -0.46145800

H -9.56859500 -1.13014500 -1.62842700

H -7.17611100 -1.68022800 -1.33689200

H -6.66544700 1.87147400 1.04420700

H -9.05550000 2.41446500 0.74846000

O -10.78130000 0.90675600 -0.66497300

H -11.03763500 1.72529600 -0.21946900

C -5.31404400 -0.21130300 0.08916800

H -4.77576800 0.51950400 0.69141500

C -4.58734400 -1.25205900 -0.37127600

H -5.00394900 -2.04664700 -0.98005400

C -3.16000600 -1.35100400 -0.05440700

O -2.51176000 -0.55317400 0.60785600

O -2.62995900 -2.48196800 -0.60423700

C -1.26192600 -2.74028200 -0.43525000

C -0.32403100 -2.06504100 -1.21377900

C -0.88361500 -3.73448000 0.46243700

C 1.02860900 -2.38228100 -1.07388200

H -0.65286700 -1.31207400 -1.92274800

C 0.47161800 -4.05097500 0.58725600

H -1.64387000 -4.25179400 1.03885500

C 1.42781100 -3.37372700 -0.17272000

H 1.77096300 -1.85183500 -1.66201300

H 0.77683000 -4.83144300 1.27851500

H 2.48214400 -3.60936400 -0.07121000

C 1.25382700 -0.73967300 2.95190100

C 0.30752000 -0.29253000 2.07811300

C 2.18951500 0.79937900 1.67190200

N 2.41952100 -0.04315400 2.68587700

H 1.20543400 -1.49511100 3.71905800

H -0.71847400 -0.58274700 1.90522000

H 2.93316400 1.44968700 1.22968800

N 0.91346800 0.66861200 1.29023900

C 3.71394800 -0.23853900 3.34711700

H 4.08787700 -1.23788500 3.12064200

H 4.41955600 0.49278200 2.95383100

H 3.59140200 -0.10598300 4.42410100

C 0.25896600 1.44111900 0.21559800

H 0.97231300 1.50535400 -0.61008500

H -0.59737500 0.84500300 -0.10413700

C -0.18547300 2.82926700 0.68622600

H -0.86149900 2.72173200 1.54430100

H 0.68845300 3.39389400 1.03643000

C -0.88634100 3.60727300 -0.43543600

H -0.20917800 3.69180900 -1.29496200

H -1.75606200 3.03395600 -0.78253400

C -1.33314100 5.00477700 0.00468400

H -0.47867900 5.61192800 0.32304700

H -1.83089700 5.53522800 -0.81279100

H -2.03499700 4.95192600 0.84474000

**a-ts1**

SCF energy in gas phase: -3192.21968282 a.u.

Fe -0.77276200 0.82639600 -0.48501900

Cl -2.81943900 0.86668600 -1.54991500

Cl -0.65308200 -1.08986100 0.76425800

Cl 0.19059900 -0.23386500 -2.56265900

Cl -1.45504400 2.30120700 1.18655800

C 4.90140200 -3.31148500 1.33050500

C 4.16240100 -2.38839200 0.60921900

C 4.77848800 -1.52851100 -0.32599600

C 6.16924700 -1.63949100 -0.50254800

C 6.92154200 -2.56259600 0.21603100

C 6.28872700 -3.40417100 1.13763600

H 4.42899700 -3.97272900 2.04904900

H 3.09083600 -2.33309600 0.77288900

H 6.66765500 -0.98911700 -1.21638800

H 7.99634800 -2.63041900 0.06267000

O 6.96055900 -4.32994800 1.87501200

H 7.90042500 -4.29689000 1.65272100

C 4.04644000 -0.54067500 -1.10803700

H 4.64487800 0.05341000 -1.79621400

C 2.71807900 -0.29795900 -1.06263700

H 2.01706900 -0.81955900 -0.42702500

C 2.17845700 0.73695700 -1.92033200

O 2.57476700 1.50357100 -2.71390300

O 0.99945600 1.64831500 -0.63240900

C 1.57196100 2.76885900 -0.10015400

C 1.57035700 3.96308400 -0.83242200

C 2.20900500 2.70706900 1.14638700

C 2.19909300 5.09275600 -0.30968300

H 1.07412100 3.98742100 -1.79664300

C 2.83309900 3.84308900 1.65867100

H 2.19209000 1.77379000 1.69950900

C 2.83220700 5.03837700 0.93428600

H 2.19349000 6.01903700 -0.87761900

H 3.32019200 3.79534700 2.62894500

H 3.31922900 5.92139100 1.33793800

C -4.90513200 -1.14154200 3.33310500

C -4.92419500 -2.08793200 2.35281500

C -3.96438100 -0.27767600 1.52604400

N -4.30218100 -0.01791600 2.79578500

H -5.25287700 -1.17141900 4.35300200

H -5.29018000 -3.10188600 2.35789600

H -3.47466300 0.40744200 0.84485000

N -4.33305200 -1.53061600 1.23453800

C -4.05555300 1.25013000 3.49115000

H -3.62895400 1.03818600 4.47295700

H -3.34126700 1.83036900 2.90468600

H -4.99312600 1.79954800 3.60388300

C -4.12690400 -2.19744500 -0.06967500

H -3.19447700 -1.80587500 -0.47938900

H -3.98239600 -3.25972000 0.14628200

C -5.29421400 -1.97299900 -1.03322100

H -6.22501700 -2.34640800 -0.58443800

H -5.41558800 -0.89520100 -1.19108600

C -5.04433200 -2.66084000 -2.38213800

H -4.11506300 -2.26832400 -2.81191900

H -4.88641400 -3.73634000 -2.22218900

C -6.19592000 -2.45194600 -3.37013300

H -6.34777700 -1.38735600 -3.57752700

H -5.99081600 -2.94962100 -4.32272300

H -7.13734200 -2.85485900 -2.97884000

**a-2**

SCF energy in gas phase: -3192.25238086 a.u.

Fe 1.41973700 -1.34313100 -0.34919900

Cl 0.28391900 -0.64822700 1.48395800

Cl 0.38619500 -0.48777400 -2.18789700

Cl -4.80218900 2.15012900 -0.49370200

Cl 1.16969300 -3.61220900 -0.41056900

C 2.10006400 2.82204200 -0.79750700

C 0.72627000 2.90697200 -0.66967700

C 0.11608100 4.01565200 -0.03781000

C 0.95107500 5.03942800 0.45369500

C 2.33240100 4.96399900 0.33349100

C 2.91158400 3.84982000 -0.28969100

H 2.56242200 1.96184400 -1.26860400

H 0.12657600 2.09327200 -1.06179000

H 0.50498800 5.90210100 0.94142300

H 2.96182100 5.76097700 0.72279700

O 4.25443900 3.70717200 -0.43474800

H 4.70960700 4.45071900 -0.01727300

C -1.31411000 4.13742700 0.13784800

H -1.65744100 5.04978100 0.62368100

C -2.27467400 3.24460100 -0.22310000

H -2.05638400 2.30082900 -0.70399200

C -3.65784000 3.56159100 0.05893200

O -4.16433600 4.51710900 0.55939900

O 3.15010700 -0.79309000 -0.29304200

C 4.33046200 -1.00919900 0.30718100

C 5.26212500 0.04422800 0.37845700

C 4.65804300 -2.26724800 0.84857700

C 6.49803700 -0.16625600 0.98399700

H 4.99611900 1.01106300 -0.03698100

C 5.89896900 -2.45929200 1.45027900

H 3.93380600 -3.07321500 0.78271800

C 6.82548500 -1.41472300 1.52194200

H 7.21250700 0.65102900 1.03664700

H 6.14450600 -3.43299900 1.86546700

H 7.79165500 -1.57181600 1.99218300

C -3.51192600 -2.03986200 -2.55941500

C -4.00507200 -1.38844300 -1.46873800

C -2.17997500 -2.46841000 -0.85017700

N -2.37641900 -2.71406700 -2.15091700

H -3.85656000 -2.06902400 -3.58034600

H -4.85310900 -0.73272900 -1.35741000

H -1.34395800 -2.83635100 -0.27050600

N -3.15991000 -1.66937000 -0.41122500

C -1.46716800 -3.46950800 -3.01933900

H -1.01610300 -2.78620900 -3.74047400

H -0.67555400 -3.89873800 -2.40574500

H -2.02615700 -4.25720000 -3.52852200

C -3.32115200 -1.19127900 0.97772400

H -2.31613600 -1.09684100 1.39647800

H -3.75280100 -0.18945100 0.91021300

C -4.20166400 -2.12318500 1.81552200

H -5.18604300 -2.22663000 1.33997200

H -3.75247700 -3.12470100 1.83516900

C -4.36899100 -1.60195200 3.24945400

H -3.37943100 -1.49103300 3.71050500

H -4.80961600 -0.59696500 3.22018500

C -5.23957000 -2.52019000 4.11266000

H -4.80489700 -3.52301500 4.18869400

H -5.34164100 -2.12453400 5.12757100

H -6.24647100 -2.62413800 3.69298300

**a-3**

SCF energy in gas phase: -3307.94463891 a.u.

Fe -2.20370900 -1.86104400 0.16725600

Cl -1.77659300 -4.09829700 0.32443900

Cl -0.57624100 -0.94302300 -1.14808500

Cl 4.72040100 0.94634900 -0.19968500

Cl -1.98397200 -0.92453600 2.21279600

C -1.90663100 3.18431300 -0.31044200

C -0.56547000 2.88888700 -0.14552000

C 0.29395000 3.76848400 0.55535500

C -0.25879100 4.95114100 1.08847400

C -1.60304300 5.25595000 0.93069200

C -2.43308700 4.37040700 0.22954400

H -2.56905000 2.50794700 -0.83910100

H -0.19000900 1.95546600 -0.55251300

H 0.38309000 5.63921000 1.63199300

H -2.01152300 6.17313000 1.34872700

O -3.75680900 4.60524800 0.04348100

H -4.00275500 5.43923100 0.46589100

C 1.70359300 3.52179400 0.75556500

H 2.22970600 4.26456300 1.35331200

C 2.45261600 2.48887400 0.28326200

H 2.05746900 1.69916300 -0.34073800

C 3.85521700 2.46040900 0.61995500

O 4.53530200 3.15902900 1.30180600

O -3.84117700 -1.58791600 -0.56657300

C -4.74139100 -0.64933300 -0.88409800

C -5.70518800 -0.93591700 -1.86921500

C -4.74695000 0.61000500 -0.25317600

C -6.64704600 0.02738200 -2.21986100

H -5.68977100 -1.91324100 -2.34070200

C -5.69196800 1.56600100 -0.61975300

H -4.01246900 0.81302000 0.52010100

C -6.64508100 1.28241200 -1.60284100

H -7.38622300 -0.20145300 -2.98287900

H -5.67229100 2.54078400 -0.14127300

H -7.37871400 2.03155300 -1.88545200

C 2.55521000 -2.18740900 2.81797000

C 3.21031400 -1.88417700 1.66128900

C 1.37226300 -2.99291600 1.13463200

N 1.41287500 -2.88427900 2.46770400

H 2.79263300 -1.95964700 3.84450200

H 4.11700600 -1.32847200 1.48214100

H 0.56718700 -3.45959900 0.57908000

N 2.45468500 -2.39613100 0.62220300

C 0.37377600 -3.36361000 3.38761200

H 0.83656900 -3.98351200 4.15802500

H -0.13980300 -2.50836100 3.82869700

H -0.35132900 -3.94408900 2.81776500

C 2.78230800 -2.32711100 -0.81607000

H 1.83130500 -2.26311900 -1.34999500

H 3.31193600 -1.38386100 -0.96850500

C 3.62232700 -3.52046300 -1.28069800

H 4.54901800 -3.56778000 -0.69365400

H 3.07334900 -4.44985900 -1.08089900

C 3.95754600 -3.42786600 -2.77561600

H 3.02528600 -3.36981900 -3.35178300

H 4.49696500 -2.49146500 -2.96885300

C 4.79317500 -4.61366900 -3.26736500

H 4.26331300 -5.56105500 -3.11915800

H 5.01666200 -4.52131400 -4.33438300

H 5.74685000 -4.67780000 -2.73154400

C 5.94743800 4.71285500 -1.39314600

H 5.57277500 5.73563800 -1.49176900

H 6.89491500 4.64591000 -1.94913600

H 6.14950700 4.52222000 -0.32996600

O 4.94671700 3.85295200 -1.91574800

H 5.26798400 2.94506600 -1.83982900

**a-ts2**

SCF energy in gas phase: -3307.92754509 a.u.

Fe -2.51989600 -1.57254000 0.09466600

Cl -2.21423300 -3.81350500 0.35894100

Cl -0.83761800 -0.78479800 -1.22990700

Cl 5.06850300 0.35263100 0.20244000

Cl -2.30510700 -0.55126700 2.10372300

C -1.32729700 2.90855500 0.00089300

C 0.04574500 2.78332300 -0.08145900

C 0.90369800 3.65297500 0.63455600

C 0.31610000 4.64691100 1.44600500

C -1.06079800 4.77848200 1.53956400

C -1.88908000 3.90776500 0.81530200

H -1.98127500 2.23399600 -0.53914000

H 0.44936900 1.98451100 -0.69451600

H 0.95532600 5.32310000 2.00745500

H -1.49740500 5.54882300 2.17069000

O -3.24008700 3.98028300 0.86412100

H -3.51318900 4.66379200 1.49088800

C 2.34287200 3.57874100 0.57810200

H 2.87286800 4.28709700 1.21290200

C 3.10060000 2.73539900 -0.18039900

H 2.71246000 1.98333200 -0.84925300

C 4.50872400 2.84990000 -0.06750400

O 5.40004900 3.45716500 0.36920500

O -4.12584500 -1.23440900 -0.68713000

C -4.89774100 -0.23017000 -1.12361700

C -5.79304200 -0.47011300 -2.18314100

C -4.83955300 1.05124300 -0.54226900

C -6.60283400 0.55903600 -2.65547300

H -5.82907300 -1.46475500 -2.61576000

C -5.65252300 2.07173200 -1.03014800

H -4.16559100 1.22486000 0.29045900

C -6.53691000 1.83505200 -2.08649500

H -7.28899600 0.36498700 -3.47560200

H -5.58505400 3.05847800 -0.58114500

H -7.16827000 2.63474100 -2.46250800

C 2.07886000 -1.99951500 3.03808100

C 2.81369700 -1.73946100 1.91891900

C 0.97648600 -2.80865200 1.30256800

N 0.93836000 -2.67120300 2.63339000

H 2.25959300 -1.75788900 4.07288800

H 3.74396300 -1.20865600 1.76770700

H 0.19467800 -3.26351200 0.70749000

N 2.10581800 -2.25411100 0.84761300

C -0.16764400 -3.09796500 3.49689700

H 0.22642300 -3.71762700 4.30506800

H -0.67711100 -2.21842000 3.89297800

H -0.87785800 -3.66585500 2.89640200

C 2.53277800 -2.22782400 -0.56617000

H 1.63106600 -2.07576700 -1.16464300

H 3.17788700 -1.35211200 -0.67180400

C 3.27807600 -3.50372900 -0.96923900

H 4.14841100 -3.63624700 -0.31395000

H 2.62630900 -4.37418900 -0.81627200

C 3.73583800 -3.44933300 -2.43281800

H 2.86266300 -3.29588800 -3.08028300

H 4.38352400 -2.57449900 -2.57327600

C 4.48018600 -4.71595200 -2.86633200

H 3.84534800 -5.60338500 -2.76623900

H 4.79578200 -4.64970900 -3.91210500

H 5.37667500 -4.87728200 -2.25731700

C 6.28972300 2.81183400 -2.39622500

H 6.07225500 3.73663200 -2.93409600

H 6.82877000 2.12772600 -3.05909000

H 6.91357200 3.03602200 -1.52268200

O 5.04032400 2.23729700 -2.00619900

H 5.20735600 1.34677000 -1.60948300

**a-4**

SCF energy in gas phase: -3307.98266174 a.u.

Fe -3.06190500 -0.93533600 -0.14490000

Cl -1.26145200 -1.75386600 -1.26020600

Cl -2.94820700 1.33079900 -0.30072400

Cl 6.63645200 1.56225500 -1.76797300

Cl -2.89719500 -1.49396200 2.06735000

C 2.01540600 0.32582800 2.59818800

C 3.20951300 -0.18917900 2.12419800

C 3.26404200 -1.46076200 1.51104600

C 2.07010900 -2.20787700 1.44753400

C 0.86154000 -1.69745000 1.90313000

C 0.81994900 -0.40785500 2.45980000

H 1.97332200 1.29666000 3.08188900

H 4.11908100 0.39247800 2.24095700

H 2.08811600 -3.19915100 1.00201800

H -0.05232000 -2.27436100 1.81026000

O -0.31775200 0.18939400 2.86676800

H -1.10095700 -0.37598200 2.68775300

C 4.47045600 -1.99626400 0.90448900

H 4.45135000 -3.05164100 0.63425300

C 5.59570300 -1.31406600 0.58933400

H 5.70314200 -0.25163800 0.77972100

C 6.71091900 -1.99051300 -0.07370000

O 6.83629000 -3.17631300 -0.29593000

O -4.61138600 -1.58274500 -0.82735900

C -5.94258300 -1.45238700 -0.83657400

C -6.71146800 -2.40022000 -1.53942100

C -6.58578600 -0.39390800 -0.16506200

C -8.09808100 -2.28742900 -1.56480400

H -6.19750600 -3.20801900 -2.05024800

C -7.97382100 -0.29558900 -0.20064000

H -5.98413200 0.33513300 0.36852900

C -8.73739600 -1.23754300 -0.89741100

H -8.68488800 -3.02311900 -2.10794200

H -8.46376800 0.52267200 0.31991000

H -9.81984500 -1.15445400 -0.91973600

C 2.81721000 1.87871300 -1.07562700

C 2.23502200 2.83365200 -0.29703900

C 0.72426400 1.26925100 -0.68475300

N 1.85522700 0.91283200 -1.30847600

H 3.82274400 1.79299300 -1.46005300

H 2.63497700 3.74634000 0.11418200

H -0.20171500 0.70870600 -0.69219900

N 0.93216700 2.43538900 -0.06092000

C 2.05161400 -0.31786800 -2.08351200

H 2.30086700 -0.06019000 -3.11478300

H 2.86314500 -0.89361300 -1.63541000

H 1.12915100 -0.89744700 -2.05544300

C -0.06551400 3.17618500 0.74015100

H -0.79858800 2.44776600 1.08947000

H 0.46469600 3.56834200 1.61288600

C -0.73653500 4.29690700 -0.05712400

H 0.02651800 4.99102600 -0.43501100

H -1.23686600 3.85724600 -0.92734800

C -1.76108600 5.05711100 0.79584200

H -2.51402800 4.34825500 1.16056900

H -1.26397600 5.47134100 1.68375600

C -2.44961000 6.18366500 0.01941200

H -2.98682500 5.79075700 -0.85026500

H -3.17473700 6.70796000 0.64900800

H -1.72450100 6.92262800 -0.34043500

C 8.82753200 -1.63955400 -1.12008200

H 9.33935500 -2.32584700 -0.44270500

H 9.46943900 -0.79424300 -1.36747700

H 8.53406800 -2.18134400 -2.02178800

O 7.67236900 -1.07439200 -0.46811000

H 7.07472600 0.45844500 -1.20193100

**a-5**

SCF energy in gas phase: -3307.97983645 a.u.

Fe -2.21824600 -0.69903400 -0.26175000

Cl -3.08111400 0.96450000 -1.53341600

Cl -0.79995400 0.18370900 1.24409500

Cl -4.61900900 -0.18318000 3.19395500

Cl -1.01587300 -2.04952300 -1.63116000

C 3.67524500 0.57066400 -1.40383500

C 4.92187400 0.24238800 -0.89266400

C 5.13902900 -0.97952100 -0.22372600

C 4.04416800 -1.85407300 -0.09280700

C 2.78778500 -1.53845700 -0.59902200

C 2.60098000 -0.31918500 -1.25892300

H 3.51614400 1.50970600 -1.92514800

H 5.74154800 0.94319800 -1.01701800

H 4.18381300 -2.80157600 0.42054600

H 1.95416000 -2.22425100 -0.48161000

O 1.39370200 0.05529900 -1.77834300

H 0.73711900 -0.66616900 -1.68193100

C 6.42962500 -1.37366900 0.33053700

H 6.46097500 -2.34491600 0.82264400

C 7.58759900 -0.68512300 0.30891300

H 7.69065200 0.29296400 -0.14921900

C 8.79711200 -1.25571200 0.92740700

O 8.88144600 -2.33462400 1.48598100

O -3.56922700 -1.61024200 0.60432400

C -4.45914800 -2.55898300 0.21445900

C -5.01295800 -2.55166300 -1.07639400

C -4.84092100 -3.54642600 1.13702400

C -5.93333800 -3.53400800 -1.43582100

H -4.72378700 -1.77273300 -1.77516800

C -5.76380500 -4.52009700 0.76312800

H -4.40628200 -3.53281700 2.13121000

C -6.31303300 -4.52102100 -0.52226000

H -6.35868700 -3.52513100 -2.43541500

H -6.05499500 -5.28250900 1.48006200

H -7.03176700 -5.28281700 -0.80862300

C -1.05602300 4.31523100 -2.58948700

C -1.15794300 4.76341700 -1.30697500

C -0.22925400 2.76006800 -1.25612800

N -0.46509800 3.06734600 -2.53762600

H -1.35021000 4.76923200 -3.52190000

H -1.55017300 5.68623600 -0.91219900

H 0.21267300 1.83531500 -0.91083100

N -0.63249300 3.77949100 -0.48918300

C -0.27756800 2.15558400 -3.67222200

H 0.06361900 2.73054000 -4.53442200

H 0.46756500 1.40892000 -3.39884900

H -1.22696200 1.66153500 -3.89046500

C -0.59193900 3.81304400 0.98827700

H -0.02517500 2.93556400 1.30370900

H -0.03537300 4.70980000 1.27965000

C -1.99142700 3.79349700 1.60867200

H -2.56135900 4.66777700 1.26635400

H -2.52041600 2.90276400 1.25437000

C -1.92405000 3.78865000 3.14207600

H -1.36471900 2.90414200 3.47059800

H -1.35855100 4.66550200 3.48794000

C -3.31367300 3.77970000 3.78655200

H -3.87339900 2.88504100 3.49685300

H -3.23702600 3.78616600 4.87802400

H -3.89682700 4.65857500 3.48790100

C 11.07901500 -0.87185400 1.36908800

H 10.96747800 -1.04265700 2.44329300

H 11.81065200 -0.08534600 1.18172900

H 11.39473800 -1.80762300 0.89985500

O 9.85004100 -0.40554100 0.79773100

H -4.12854600 -0.77484700 2.12941200

**a-ts3**

SCF energy in gas phase: -3307.97258908 a.u.

Fe 2.36279600 0.63996600 -0.79149100

Cl 2.99855700 -1.16711100 -1.91785900

Cl 0.94903600 0.21568100 0.87999700

Cl 4.75228900 -0.09590700 1.25507700

Cl 1.06062900 1.75862900 -2.27078800

C -3.43631100 -1.11993300 -1.27282300

C -4.67007900 -0.75550500 -0.75486100

C -4.92512300 0.56573100 -0.33434800

C -3.88139200 1.50154100 -0.45859300

C -2.63852200 1.15103400 -0.97506100

C -2.41323800 -0.16726900 -1.38523100

H -3.24970700 -2.13748400 -1.60238300

H -5.44994400 -1.50678700 -0.67845400

H -4.05057200 2.52641800 -0.13952300

H -1.84389200 1.88664000 -1.05507100

O -1.21636800 -0.58126200 -1.89935400

H -0.60667600 0.17654100 -1.99749900

C -6.20422300 1.00138600 0.21553900

H -6.26765800 2.05018200 0.50273500

C -7.31682200 0.26728200 0.41183600

H -7.38532400 -0.78768400 0.16787500

C -8.52226400 0.89290500 0.98352600

O -8.64044700 2.05772700 1.31880100

O 3.81880000 1.91879100 -0.36034000

C 3.72585200 3.24419400 0.05614000

C 3.44738600 4.22605600 -0.89490800

C 3.93998800 3.56553400 1.39868000

C 3.37670700 5.55780000 -0.48678200

H 3.28942800 3.94087000 -1.92898900

C 3.87374500 4.90384000 1.78639100

H 4.15740500 2.77699100 2.11170300

C 3.59031200 5.90125900 0.85047300

H 3.15887200 6.32879800 -1.21991200

H 4.04231400 5.16420300 2.82715100

H 3.53681100 6.94027300 1.16102600

C 1.28848600 -4.98046700 -1.48236700

C 1.59697400 -4.98519300 -0.15541800

C 0.58232300 -3.07773600 -0.60963500

N 0.64879600 -3.78403000 -1.74581300

H 1.46911400 -5.71293100 -2.25245200

H 2.09182000 -5.72683300 0.44958200

H 0.15157100 -2.09083600 -0.51447400

N 1.14562500 -3.78958800 0.37308000

C 0.23745000 -3.30247800 -3.06935900

H -0.28736200 -4.10447500 -3.59150400

H -0.42028200 -2.44338000 -2.93931600

H 1.12307500 -2.99764100 -3.63064600

C 1.29667400 -3.35564300 1.78132700

H 0.80690300 -2.38362500 1.85892200

H 0.74677300 -4.07256700 2.40042400

C 2.76197500 -3.25187500 2.21097500

H 3.25412200 -4.22672500 2.08989800

H 3.28240200 -2.53558400 1.56604500

C 2.87707800 -2.79941900 3.67388700

H 2.41587900 -1.80941600 3.77449400

H 2.30588200 -3.48242200 4.31935300

C 4.33260600 -2.72965500 4.14594000

H 4.89919700 -2.01833000 3.53821700

H 4.38833700 -2.40580600 5.18999000

H 4.82090300 -3.70880100 4.07454700

C -10.74833400 0.49735500 1.64528400

H -10.58776200 0.88819600 2.65373800

H -11.44008200 -0.34520800 1.66830000

H -11.14588700 1.30175300 1.02047200

O -9.52694200 -0.01558000 1.09795700

H 4.36417200 1.25807200 0.33419200

**a-6**

SCF energy in gas phase: -3307.99787640 a.u.

Fe 2.24435700 0.19748500 -0.89590000

Cl 3.30239000 1.61970800 0.46327400

Cl 0.45701500 1.26245100 -1.72966200

Cl 3.58564600 -0.52913900 -2.50361900

Cl 1.45995400 -1.52285000 0.33553900

C -3.57484500 0.05790200 1.52029000

C -4.85354400 -0.30857000 1.12802900

C -5.06135800 -1.27915400 0.12672900

C -3.92404700 -1.86398100 -0.46037600

C -2.63531600 -1.50586400 -0.07829500

C -2.45920100 -0.53959500 0.91706300

H -3.42017100 0.80035500 2.29714000

H -5.70520000 0.16340500 1.60789000

H -4.05610800 -2.61407700 -1.23533900

H -1.77076100 -1.96570200 -0.54819000

O -1.22096500 -0.13870900 1.33705800

H -0.51874600 -0.65969200 0.89559400

C -6.38459400 -1.69949400 -0.32238800

H -6.40486300 -2.45968400 -1.10220700

C -7.58404700 -1.26282800 0.10818000

H -7.70209000 -0.50892600 0.87944800

C -8.82177000 -1.81285500 -0.47314100

O -8.89414000 -2.66133400 -1.34361700

O 5.69495500 -1.76024100 -0.14305000

C 5.70298600 -3.11481700 0.01057200

C 6.46137700 -3.63838200 1.06583200

C 4.99712900 -3.97985700 -0.83592400

C 6.50923900 -5.01580400 1.26835500

H 7.00286400 -2.95168000 1.70877000

C 5.05399900 -5.35746700 -0.62091300

H 4.40771300 -3.57241000 -1.65318800

C 5.80764900 -5.88566500 0.42857800

H 7.10133600 -5.41260700 2.08893300

H 4.50186300 -6.01971700 -1.28242700

H 5.84848500 -6.95854700 0.59036100

C 1.49996800 3.54060900 3.24440600

C 1.23398100 4.47834300 2.29214600

C 0.24923700 2.59616600 1.68886900

N 0.86857400 2.37567400 2.85459300

H 2.08106900 3.59975200 4.15042800

H 1.53295700 5.51097100 2.21915900

H -0.31870600 1.85478700 1.14220600

N 0.44876000 3.86946000 1.33034400

C 0.98635400 1.06812800 3.51197500

H 0.94687900 1.21156800 4.59265000

H 0.15745300 0.43982300 3.18732900

H 1.93284100 0.60652000 3.22162600

C -0.02747900 4.48657900 0.07507800

H -0.64411400 3.73602100 -0.42305600

H -0.66535500 5.33506700 0.34382800

C 1.12559800 4.91611100 -0.83598900

H 1.73670800 5.67480000 -0.32927700

H 1.76963600 4.04964100 -1.01743700

C 0.60855800 5.47596700 -2.16821800

H 0.01275500 4.70433200 -2.67138000

H -0.06855000 6.32010900 -1.97709500

C 1.74491300 5.92577300 -3.09168600

H 2.41397100 5.09139600 -3.32613900

H 1.35218200 6.31445100 -4.03593900

H 2.34552700 6.71694400 -2.62889500

C -11.17428900 -1.71137200 -0.40227800

H -11.26372000 -1.51697200 -1.47450800

H -11.93640800 -1.16112200 0.15025800

H -11.28113600 -2.78643200 -0.23413800

O -9.91527400 -1.24128100 0.09656700

H 5.15236600 -1.51249900 -0.90862300

**MPC+cat+phenol**

SCF energy in gas phase: -3308.00468983 a.u.

C -2.92444200 3.87091700 0.27652700

C -2.40061500 2.59134000 0.32163300

C -2.62350200 1.67710100 -0.72656600

C -3.37325600 2.11029000 -1.83110100

C -3.90942600 3.39058000 -1.88387100

C -3.68769900 4.27412500 -0.82450500

H -2.74210000 4.57930100 1.07513400

H -1.78560000 2.30772500 1.16618200

H -3.54910800 1.42579000 -2.65394400

H -4.49342000 3.70597800 -2.74335600

O -4.18053500 5.54675000 -0.80964700

H -4.67111100 5.72114100 -1.61979900

C -2.10169200 0.32283800 -0.71300000

H -2.11762600 -0.21118900 -1.65845300

C -1.62783700 -0.34685400 0.35495100

H -1.62411900 0.09400000 1.33955400

C -1.12247200 -1.70345700 0.17340700

O -0.79652000 -2.18393600 -0.90060900

O -3.12907100 -4.26193100 -0.06054600

C -3.91255800 -3.13897400 -0.17923000

C -4.38241700 -2.44999500 0.94119400

C -4.24731000 -2.69771800 -1.45870800

C -5.15572100 -1.30230000 0.77863600

H -4.14107300 -2.82016000 1.93139500

C -5.03056500 -1.55655000 -1.61182100

H -3.86272800 -3.24161000 -2.31251400

C -5.48000800 -0.84725800 -0.49740000

H -5.50303300 -0.76262200 1.65255900

H -5.27948600 -1.21137600 -2.60961100

H -6.06914200 0.05322500 -0.62258900

C 3.84279200 -3.52425800 -0.96567400

C 4.37953800 -2.44406900 -1.59250800

C 2.21277200 -2.08409700 -1.34884000

N 2.48884200 -3.28717700 -0.83369200

H 4.29944600 -4.42790800 -0.60186100

H 5.39194400 -2.22902800 -1.88374000

H 1.23788100 -1.62068300 -1.35117800

N 3.34404600 -1.55966000 -1.82993700

C 1.54910400 -4.13967500 -0.09611000

H 1.77129200 -5.18118600 -0.32614200

H 1.66396900 -3.94563300 0.97035800

H 0.53524500 -3.88471000 -0.39391900

C 3.48025100 -0.23214000 -2.46159200

H 3.82762400 -0.38817600 -3.48645500

H 2.48153800 0.20047900 -2.49462100

C 4.42648100 0.67858800 -1.68222300

H 4.09885500 0.72878300 -0.64148700

H 5.43712800 0.25288400 -1.68136700

C 4.45947400 2.08874400 -2.27987800

H 4.72606100 2.03282600 -3.34337700

H 3.45210700 2.51294700 -2.22486400

C 5.44133500 3.00477700 -1.54471800

H 6.46364600 2.61661400 -1.60205300

H 5.44005200 4.00836900 -1.97776600

H 5.17125500 3.09017100 -0.48988100

Fe 1.79680500 1.03904100 1.49454500

Cl 3.68497200 2.09578900 1.94049100

Cl 1.14637800 1.51609500 -0.59015600

Cl 2.16489400 -1.17835500 1.62399900

Cl 0.18160500 1.54736000 2.94262300

C -1.21171300 -2.02156300 2.59880800

H -0.45523200 -1.27071600 2.82453700

H -1.07599500 -2.88314200 3.25014000

H -2.21426900 -1.61201000 2.73238800

O -1.03715600 -2.53033800 1.25760900

H -2.44139400 -4.08913700 0.59676700

**a-1**

SCF energy in solvent model (SMD): -3192.34396261 a.u.

Fe 5.15651700 0.37682400 -0.85204400

Cl 5.34561500 1.98521500 0.73351300

Cl 3.25186200 0.80796900 -1.97079200

Cl 6.90141400 0.34978400 -2.18316600

Cl 4.88538400 -1.57687000 0.22194200

C -8.91748500 -0.48632200 -1.04693200

C -7.57616900 -0.78281600 -0.87584300

C -6.72875400 0.05629800 -0.11914600

C -7.29513500 1.20830700 0.45714500

C -8.64053800 1.51803900 0.29333200

C -9.45949800 0.66974400 -0.46145800

H -9.56859500 -1.13014500 -1.62842700

H -7.17611100 -1.68022800 -1.33689200

H -6.66544700 1.87147400 1.04420700

H -9.05550000 2.41446500 0.74846000

O -10.78130000 0.90675600 -0.66497300

H -11.03763500 1.72529600 -0.21946900

C -5.31404400 -0.21130300 0.08916800

H -4.77576800 0.51950400 0.69141500

C -4.58734400 -1.25205900 -0.37127600

H -5.00394900 -2.04664700 -0.98005400

C -3.16000600 -1.35100400 -0.05440700

O -2.51176000 -0.55317400 0.60785600

O -2.62995900 -2.48196800 -0.60423700

C -1.26192600 -2.74028200 -0.43525000

C -0.32403100 -2.06504100 -1.21377900

C -0.88361500 -3.73448000 0.46243700

C 1.02860900 -2.38228100 -1.07388200

H -0.65286700 -1.31207400 -1.92274800

C 0.47161800 -4.05097500 0.58725600

H -1.64387000 -4.25179400 1.03885500

C 1.42781100 -3.37372700 -0.17272000

H 1.77096300 -1.85183500 -1.66201300

H 0.77683000 -4.83144300 1.27851500

H 2.48214400 -3.60936400 -0.07121000

C 1.25382700 -0.73967300 2.95190100

C 0.30752000 -0.29253000 2.07811300

C 2.18951500 0.79937900 1.67190200

N 2.41952100 -0.04315400 2.68587700

H 1.20543400 -1.49511100 3.71905800

H -0.71847400 -0.58274700 1.90522000

H 2.93316400 1.44968700 1.22968800

N 0.91346800 0.66861200 1.29023900

C 3.71394800 -0.23853900 3.34711700

H 4.08787700 -1.23788500 3.12064200

H 4.41955600 0.49278200 2.95383100

H 3.59140200 -0.10598300 4.42410100

C 0.25896600 1.44111900 0.21559800

H 0.97231300 1.50535400 -0.61008500

H -0.59737500 0.84500300 -0.10413700

C -0.18547300 2.82926700 0.68622600

H -0.86149900 2.72173200 1.54430100

H 0.68845300 3.39389400 1.03643000

C -0.88634100 3.60727300 -0.43543600

H -0.20917800 3.69180900 -1.29496200

H -1.75606200 3.03395600 -0.78253400

C -1.33314100 5.00477700 0.00468400

H -0.47867900 5.61192800 0.32304700

H -1.83089700 5.53522800 -0.81279100

H -2.03499700 4.95192600 0.84474000

**a-ts1**

SCF energy in solvent model (SMD): -3192.28603538 a.u.

Fe -0.77276200 0.82639600 -0.48501900

Cl -2.81943900 0.86668600 -1.54991500

Cl -0.65308200 -1.08986100 0.76425800

Cl 0.19059900 -0.23386500 -2.56265900

Cl -1.45504400 2.30120700 1.18655800

C 4.90140200 -3.31148500 1.33050500

C 4.16240100 -2.38839200 0.60921900

C 4.77848800 -1.52851100 -0.32599600

C 6.16924700 -1.63949100 -0.50254800

C 6.92154200 -2.56259600 0.21603100

C 6.28872700 -3.40417100 1.13763600

H 4.42899700 -3.97272900 2.04904900

H 3.09083600 -2.33309600 0.77288900

H 6.66765500 -0.98911700 -1.21638800

H 7.99634800 -2.63041900 0.06267000

O 6.96055900 -4.32994800 1.87501200

H 7.90042500 -4.29689000 1.65272100

C 4.04644000 -0.54067500 -1.10803700

H 4.64487800 0.05341000 -1.79621400

C 2.71807900 -0.29795900 -1.06263700

H 2.01706900 -0.81955900 -0.42702500

C 2.17845700 0.73695700 -1.92033200

O 2.57476700 1.50357100 -2.71390300

O 0.99945600 1.64831500 -0.63240900

C 1.57196100 2.76885900 -0.10015400

C 1.57035700 3.96308400 -0.83242200

C 2.20900500 2.70706900 1.14638700

C 2.19909300 5.09275600 -0.30968300

H 1.07412100 3.98742100 -1.79664300

C 2.83309900 3.84308900 1.65867100

H 2.19209000 1.77379000 1.69950900

C 2.83220700 5.03837700 0.93428600

H 2.19349000 6.01903700 -0.87761900

H 3.32019200 3.79534700 2.62894500

H 3.31922900 5.92139100 1.33793800

C -4.90513200 -1.14154200 3.33310500

C -4.92419500 -2.08793200 2.35281500

C -3.96438100 -0.27767600 1.52604400

N -4.30218100 -0.01791600 2.79578500

H -5.25287700 -1.17141900 4.35300200

H -5.29018000 -3.10188600 2.35789600

H -3.47466300 0.40744200 0.84485000

N -4.33305200 -1.53061600 1.23453800

C -4.05555300 1.25013000 3.49115000

H -3.62895400 1.03818600 4.47295700

H -3.34126700 1.83036900 2.90468600

H -4.99312600 1.79954800 3.60388300

C -4.12690400 -2.19744500 -0.06967500

H -3.19447700 -1.80587500 -0.47938900

H -3.98239600 -3.25972000 0.14628200

C -5.29421400 -1.97299900 -1.03322100

H -6.22501700 -2.34640800 -0.58443800

H -5.41558800 -0.89520100 -1.19108600

C -5.04433200 -2.66084000 -2.38213800

H -4.11506300 -2.26832400 -2.81191900

H -4.88641400 -3.73634000 -2.22218900

C -6.19592000 -2.45194600 -3.37013300

H -6.34777700 -1.38735600 -3.57752700

H -5.99081600 -2.94962100 -4.32272300

H -7.13734200 -2.85485900 -2.97884000

**a-2**

SCF energy in solvent model (SMD): -3192.31054154 a.u.

Fe 1.41973700 -1.34313100 -0.34919900

Cl 0.28391900 -0.64822700 1.48395800

Cl 0.38619500 -0.48777400 -2.18789700

Cl -4.80218900 2.15012900 -0.49370200

Cl 1.16969300 -3.61220900 -0.41056900

C 2.10006400 2.82204200 -0.79750700

C 0.72627000 2.90697200 -0.66967700

C 0.11608100 4.01565200 -0.03781000

C 0.95107500 5.03942800 0.45369500

C 2.33240100 4.96399900 0.33349100

C 2.91158400 3.84982000 -0.28969100

H 2.56242200 1.96184400 -1.26860400

H 0.12657600 2.09327200 -1.06179000

H 0.50498800 5.90210100 0.94142300

H 2.96182100 5.76097700 0.72279700

O 4.25443900 3.70717200 -0.43474800

H 4.70960700 4.45071900 -0.01727300

C -1.31411000 4.13742700 0.13784800

H -1.65744100 5.04978100 0.62368100

C -2.27467400 3.24460100 -0.22310000

H -2.05638400 2.30082900 -0.70399200

C -3.65784000 3.56159100 0.05893200

O -4.16433600 4.51710900 0.55939900

O 3.15010700 -0.79309000 -0.29304200

C 4.33046200 -1.00919900 0.30718100

C 5.26212500 0.04422800 0.37845700

C 4.65804300 -2.26724800 0.84857700

C 6.49803700 -0.16625600 0.98399700

H 4.99611900 1.01106300 -0.03698100

C 5.89896900 -2.45929200 1.45027900

H 3.93380600 -3.07321500 0.78271800

C 6.82548500 -1.41472300 1.52194200

H 7.21250700 0.65102900 1.03664700

H 6.14450600 -3.43299900 1.86546700

H 7.79165500 -1.57181600 1.99218300

C -3.51192600 -2.03986200 -2.55941500

C -4.00507200 -1.38844300 -1.46873800

C -2.17997500 -2.46841000 -0.85017700

N -2.37641900 -2.71406700 -2.15091700

H -3.85656000 -2.06902400 -3.58034600

H -4.85310900 -0.73272900 -1.35741000

H -1.34395800 -2.83635100 -0.27050600

N -3.15991000 -1.66937000 -0.41122500

C -1.46716800 -3.46950800 -3.01933900

H -1.01610300 -2.78620900 -3.74047400

H -0.67555400 -3.89873800 -2.40574500

H -2.02615700 -4.25720000 -3.52852200

C -3.32115200 -1.19127900 0.97772400

H -2.31613600 -1.09684100 1.39647800

H -3.75280100 -0.18945100 0.91021300

C -4.20166400 -2.12318500 1.81552200

H -5.18604300 -2.22663000 1.33997200

H -3.75247700 -3.12470100 1.83516900

C -4.36899100 -1.60195200 3.24945400

H -3.37943100 -1.49103300 3.71050500

H -4.80961600 -0.59696500 3.22018500

C -5.23957000 -2.52019000 4.11266000

H -4.80489700 -3.52301500 4.18869400

H -5.34164100 -2.12453400 5.12757100

H -6.24647100 -2.62413800 3.69298300

**a-3**

SCF energy in solvent model (SMD): -3308.00967976 a.u.

Fe -2.20370900 -1.86104400 0.16725600

Cl -1.77659300 -4.09829700 0.32443900

Cl -0.57624100 -0.94302300 -1.14808500

Cl 4.72040100 0.94634900 -0.19968500

Cl -1.98397200 -0.92453600 2.21279600

C -1.90663100 3.18431300 -0.31044200

C -0.56547000 2.88888700 -0.14552000

C 0.29395000 3.76848400 0.55535500

C -0.25879100 4.95114100 1.08847400

C -1.60304300 5.25595000 0.93069200

C -2.43308700 4.37040700 0.22954400

H -2.56905000 2.50794700 -0.83910100

H -0.19000900 1.95546600 -0.55251300

H 0.38309000 5.63921000 1.63199300

H -2.01152300 6.17313000 1.34872700

O -3.75680900 4.60524800 0.04348100

H -4.00275500 5.43923100 0.46589100

C 1.70359300 3.52179400 0.75556500

H 2.22970600 4.26456300 1.35331200

C 2.45261600 2.48887400 0.28326200

H 2.05746900 1.69916300 -0.34073800

C 3.85521700 2.46040900 0.61995500

O 4.53530200 3.15902900 1.30180600

O -3.84117700 -1.58791600 -0.56657300

C -4.74139100 -0.64933300 -0.88409800

C -5.70518800 -0.93591700 -1.86921500

C -4.74695000 0.61000500 -0.25317600

C -6.64704600 0.02738200 -2.21986100

H -5.68977100 -1.91324100 -2.34070200

C -5.69196800 1.56600100 -0.61975300

H -4.01246900 0.81302000 0.52010100

C -6.64508100 1.28241200 -1.60284100

H -7.38622300 -0.20145300 -2.98287900

H -5.67229100 2.54078400 -0.14127300

H -7.37871400 2.03155300 -1.88545200

C 2.55521000 -2.18740900 2.81797000

C 3.21031400 -1.88417700 1.66128900

C 1.37226300 -2.99291600 1.13463200

N 1.41287500 -2.88427900 2.46770400

H 2.79263300 -1.95964700 3.84450200

H 4.11700600 -1.32847200 1.48214100

H 0.56718700 -3.45959900 0.57908000

N 2.45468500 -2.39613100 0.62220300

C 0.37377600 -3.36361000 3.38761200

H 0.83656900 -3.98351200 4.15802500

H -0.13980300 -2.50836100 3.82869700

H -0.35132900 -3.94408900 2.81776500

C 2.78230800 -2.32711100 -0.81607000

H 1.83130500 -2.26311900 -1.34999500

H 3.31193600 -1.38386100 -0.96850500

C 3.62232700 -3.52046300 -1.28069800

H 4.54901800 -3.56778000 -0.69365400

H 3.07334900 -4.44985900 -1.08089900

C 3.95754600 -3.42786600 -2.77561600

H 3.02528600 -3.36981900 -3.35178300

H 4.49696500 -2.49146500 -2.96885300

C 4.79317500 -4.61366900 -3.26736500

H 4.26331300 -5.56105500 -3.11915800

H 5.01666200 -4.52131400 -4.33438300

H 5.74685000 -4.67780000 -2.73154400

C 5.94743800 4.71285500 -1.39314600

H 5.57277500 5.73563800 -1.49176900

H 6.89491500 4.64591000 -1.94913600

H 6.14950700 4.52222000 -0.32996600

O 4.94671700 3.85295200 -1.91574800

H 5.26798400 2.94506600 -1.83982900

**a-ts2**

SCF energy in solvent model (SMD): -3307.99056396 a.u.

Fe -2.51989600 -1.57254000 0.09466600

Cl -2.21423300 -3.81350500 0.35894100

Cl -0.83761800 -0.78479800 -1.22990700

Cl 5.06850300 0.35263100 0.20244000

Cl -2.30510700 -0.55126700 2.10372300

C -1.32729700 2.90855500 0.00089300

C 0.04574500 2.78332300 -0.08145900

C 0.90369800 3.65297500 0.63455600

C 0.31610000 4.64691100 1.44600500

C -1.06079800 4.77848200 1.53956400

C -1.88908000 3.90776500 0.81530200

H -1.98127500 2.23399600 -0.53914000

H 0.44936900 1.98451100 -0.69451600

H 0.95532600 5.32310000 2.00745500

H -1.49740500 5.54882300 2.17069000

O -3.24008700 3.98028300 0.86412100

H -3.51318900 4.66379200 1.49088800

C 2.34287200 3.57874100 0.57810200

H 2.87286800 4.28709700 1.21290200

C 3.10060000 2.73539900 -0.18039900

H 2.71246000 1.98333200 -0.84925300

C 4.50872400 2.84990000 -0.06750400

O 5.40004900 3.45716500 0.36920500

O -4.12584500 -1.23440900 -0.68713000

C -4.89774100 -0.23017000 -1.12361700

C -5.79304200 -0.47011300 -2.18314100

C -4.83955300 1.05124300 -0.54226900

C -6.60283400 0.55903600 -2.65547300

H -5.82907300 -1.46475500 -2.61576000

C -5.65252300 2.07173200 -1.03014800

H -4.16559100 1.22486000 0.29045900

C -6.53691000 1.83505200 -2.08649500

H -7.28899600 0.36498700 -3.47560200

H -5.58505400 3.05847800 -0.58114500

H -7.16827000 2.63474100 -2.46250800

C 2.07886000 -1.99951500 3.03808100

C 2.81369700 -1.73946100 1.91891900

C 0.97648600 -2.80865200 1.30256800

N 0.93836000 -2.67120300 2.63339000

H 2.25959300 -1.75788900 4.07288800

H 3.74396300 -1.20865600 1.76770700

H 0.19467800 -3.26351200 0.70749000

N 2.10581800 -2.25411100 0.84761300

C -0.16764400 -3.09796500 3.49689700

H 0.22642300 -3.71762700 4.30506800

H -0.67711100 -2.21842000 3.89297800

H -0.87785800 -3.66585500 2.89640200

C 2.53277800 -2.22782400 -0.56617000

H 1.63106600 -2.07576700 -1.16464300

H 3.17788700 -1.35211200 -0.67180400

C 3.27807600 -3.50372900 -0.96923900

H 4.14841100 -3.63624700 -0.31395000

H 2.62630900 -4.37418900 -0.81627200

C 3.73583800 -3.44933300 -2.43281800

H 2.86266300 -3.29588800 -3.08028300

H 4.38352400 -2.57449900 -2.57327600

C 4.48018600 -4.71595200 -2.86633200

H 3.84534800 -5.60338500 -2.76623900

H 4.79578200 -4.64970900 -3.91210500

H 5.37667500 -4.87728200 -2.25731700

C 6.28972300 2.81183400 -2.39622500

H 6.07225500 3.73663200 -2.93409600

H 6.82877000 2.12772600 -3.05909000

H 6.91357200 3.03602200 -1.52268200

O 5.04032400 2.23729700 -2.00619900

H 5.20735600 1.34677000 -1.60948300

**a-4**

SCF energy in solvent model (SMD): -3308.03725051 a.u.

Fe -3.06190500 -0.93533600 -0.14490000

Cl -1.26145200 -1.75386600 -1.26020600

Cl -2.94820700 1.33079900 -0.30072400

Cl 6.63645200 1.56225500 -1.76797300

Cl -2.89719500 -1.49396200 2.06735000

C 2.01540600 0.32582800 2.59818800

C 3.20951300 -0.18917900 2.12419800

C 3.26404200 -1.46076200 1.51104600

C 2.07010900 -2.20787700 1.44753400

C 0.86154000 -1.69745000 1.90313000

C 0.81994900 -0.40785500 2.45980000

H 1.97332200 1.29666000 3.08188900

H 4.11908100 0.39247800 2.24095700

H 2.08811600 -3.19915100 1.00201800

H -0.05232000 -2.27436100 1.81026000

O -0.31775200 0.18939400 2.86676800

H -1.10095700 -0.37598200 2.68775300

C 4.47045600 -1.99626400 0.90448900

H 4.45135000 -3.05164100 0.63425300

C 5.59570300 -1.31406600 0.58933400

H 5.70314200 -0.25163800 0.77972100

C 6.71091900 -1.99051300 -0.07370000

O 6.83629000 -3.17631300 -0.29593000

O -4.61138600 -1.58274500 -0.82735900

C -5.94258300 -1.45238700 -0.83657400

C -6.71146800 -2.40022000 -1.53942100

C -6.58578600 -0.39390800 -0.16506200

C -8.09808100 -2.28742900 -1.56480400

H -6.19750600 -3.20801900 -2.05024800

C -7.97382100 -0.29558900 -0.20064000

H -5.98413200 0.33513300 0.36852900

C -8.73739600 -1.23754300 -0.89741100

H -8.68488800 -3.02311900 -2.10794200

H -8.46376800 0.52267200 0.31991000

H -9.81984500 -1.15445400 -0.91973600

C 2.81721000 1.87871300 -1.07562700

C 2.23502200 2.83365200 -0.29703900

C 0.72426400 1.26925100 -0.68475300

N 1.85522700 0.91283200 -1.30847600

H 3.82274400 1.79299300 -1.46005300

H 2.63497700 3.74634000 0.11418200

H -0.20171500 0.70870600 -0.69219900

N 0.93216700 2.43538900 -0.06092000

C 2.05161400 -0.31786800 -2.08351200

H 2.30086700 -0.06019000 -3.11478300

H 2.86314500 -0.89361300 -1.63541000

H 1.12915100 -0.89744700 -2.05544300

C -0.06551400 3.17618500 0.74015100

H -0.79858800 2.44776600 1.08947000

H 0.46469600 3.56834200 1.61288600

C -0.73653500 4.29690700 -0.05712400

H 0.02651800 4.99102600 -0.43501100

H -1.23686600 3.85724600 -0.92734800

C -1.76108600 5.05711100 0.79584200

H -2.51402800 4.34825500 1.16056900

H -1.26397600 5.47134100 1.68375600

C -2.44961000 6.18366500 0.01941200

H -2.98682500 5.79075700 -0.85026500

H -3.17473700 6.70796000 0.64900800

H -1.72450100 6.92262800 -0.34043500

C 8.82753200 -1.63955400 -1.12008200

H 9.33935500 -2.32584700 -0.44270500

H 9.46943900 -0.79424300 -1.36747700

H 8.53406800 -2.18134400 -2.02178800

O 7.67236900 -1.07439200 -0.46811000

H 7.07472600 0.45844500 -1.20193100

**a-5**

SCF energy in solvent model (SMD): -3308.03842833 a.u.

Fe -2.21824600 -0.69903400 -0.26175000

Cl -3.08111400 0.96450000 -1.53341600

Cl -0.79995400 0.18370900 1.24409500

Cl -4.61900900 -0.18318000 3.19395500

Cl -1.01587300 -2.04952300 -1.63116000

C 3.67524500 0.57066400 -1.40383500

C 4.92187400 0.24238800 -0.89266400

C 5.13902900 -0.97952100 -0.22372600

C 4.04416800 -1.85407300 -0.09280700

C 2.78778500 -1.53845700 -0.59902200

C 2.60098000 -0.31918500 -1.25892300

H 3.51614400 1.50970600 -1.92514800

H 5.74154800 0.94319800 -1.01701800

H 4.18381300 -2.80157600 0.42054600

H 1.95416000 -2.22425100 -0.48161000

O 1.39370200 0.05529900 -1.77834300

H 0.73711900 -0.66616900 -1.68193100

C 6.42962500 -1.37366900 0.33053700

H 6.46097500 -2.34491600 0.82264400

C 7.58759900 -0.68512300 0.30891300

H 7.69065200 0.29296400 -0.14921900

C 8.79711200 -1.25571200 0.92740700

O 8.88144600 -2.33462400 1.48598100

O -3.56922700 -1.61024200 0.60432400

C -4.45914800 -2.55898300 0.21445900

C -5.01295800 -2.55166300 -1.07639400

C -4.84092100 -3.54642600 1.13702400

C -5.93333800 -3.53400800 -1.43582100

H -4.72378700 -1.77273300 -1.77516800

C -5.76380500 -4.52009700 0.76312800

H -4.40628200 -3.53281700 2.13121000

C -6.31303300 -4.52102100 -0.52226000

H -6.35868700 -3.52513100 -2.43541500

H -6.05499500 -5.28250900 1.48006200

H -7.03176700 -5.28281700 -0.80862300

C -1.05602300 4.31523100 -2.58948700

C -1.15794300 4.76341700 -1.30697500

C -0.22925400 2.76006800 -1.25612800

N -0.46509800 3.06734600 -2.53762600

H -1.35021000 4.76923200 -3.52190000

H -1.55017300 5.68623600 -0.91219900

H 0.21267300 1.83531500 -0.91083100

N -0.63249300 3.77949100 -0.48918300

C -0.27756800 2.15558400 -3.67222200

H 0.06361900 2.73054000 -4.53442200

H 0.46756500 1.40892000 -3.39884900

H -1.22696200 1.66153500 -3.89046500

C -0.59193900 3.81304400 0.98827700

H -0.02517500 2.93556400 1.30370900

H -0.03537300 4.70980000 1.27965000

C -1.99142700 3.79349700 1.60867200

H -2.56135900 4.66777700 1.26635400

H -2.52041600 2.90276400 1.25437000

C -1.92405000 3.78865000 3.14207600

H -1.36471900 2.90414200 3.47059800

H -1.35855100 4.66550200 3.48794000

C -3.31367300 3.77970000 3.78655200

H -3.87339900 2.88504100 3.49685300

H -3.23702600 3.78616600 4.87802400

H -3.89682700 4.65857500 3.48790100

C 11.07901500 -0.87185400 1.36908800

H 10.96747800 -1.04265700 2.44329300

H 11.81065200 -0.08534600 1.18172900

H 11.39473800 -1.80762300 0.89985500

O 9.85004100 -0.40554100 0.79773100

H -4.12854600 -0.77484700 2.12941200

**a-ts3**

SCF energy in solvent model (SMD): -3308.03315416 a.u.

Fe 2.36279600 0.63996600 -0.79149100

Cl 2.99855700 -1.16711100 -1.91785900

Cl 0.94903600 0.21568100 0.87999700

Cl 4.75228900 -0.09590700 1.25507700

Cl 1.06062900 1.75862900 -2.27078800

C -3.43631100 -1.11993300 -1.27282300

C -4.67007900 -0.75550500 -0.75486100

C -4.92512300 0.56573100 -0.33434800

C -3.88139200 1.50154100 -0.45859300

C -2.63852200 1.15103400 -0.97506100

C -2.41323800 -0.16726900 -1.38523100

H -3.24970700 -2.13748400 -1.60238300

H -5.44994400 -1.50678700 -0.67845400

H -4.05057200 2.52641800 -0.13952300

H -1.84389200 1.88664000 -1.05507100

O -1.21636800 -0.58126200 -1.89935400

H -0.60667600 0.17654100 -1.99749900

C -6.20422300 1.00138600 0.21553900

H -6.26765800 2.05018200 0.50273500

C -7.31682200 0.26728200 0.41183600

H -7.38532400 -0.78768400 0.16787500

C -8.52226400 0.89290500 0.98352600

O -8.64044700 2.05772700 1.31880100

O 3.81880000 1.91879100 -0.36034000

C 3.72585200 3.24419400 0.05614000

C 3.44738600 4.22605600 -0.89490800

C 3.93998800 3.56553400 1.39868000

C 3.37670700 5.55780000 -0.48678200

H 3.28942800 3.94087000 -1.92898900

C 3.87374500 4.90384000 1.78639100

H 4.15740500 2.77699100 2.11170300

C 3.59031200 5.90125900 0.85047300

H 3.15887200 6.32879800 -1.21991200

H 4.04231400 5.16420300 2.82715100

H 3.53681100 6.94027300 1.16102600

C 1.28848600 -4.98046700 -1.48236700

C 1.59697400 -4.98519300 -0.15541800

C 0.58232300 -3.07773600 -0.60963500

N 0.64879600 -3.78403000 -1.74581300

H 1.46911400 -5.71293100 -2.25245200

H 2.09182000 -5.72683300 0.44958200

H 0.15157100 -2.09083600 -0.51447400

N 1.14562500 -3.78958800 0.37308000

C 0.23745000 -3.30247800 -3.06935900

H -0.28736200 -4.10447500 -3.59150400

H -0.42028200 -2.44338000 -2.93931600

H 1.12307500 -2.99764100 -3.63064600

C 1.29667400 -3.35564300 1.78132700

H 0.80690300 -2.38362500 1.85892200

H 0.74677300 -4.07256700 2.40042400

C 2.76197500 -3.25187500 2.21097500

H 3.25412200 -4.22672500 2.08989800

H 3.28240200 -2.53558400 1.56604500

C 2.87707800 -2.79941900 3.67388700

H 2.41587900 -1.80941600 3.77449400

H 2.30588200 -3.48242200 4.31935300

C 4.33260600 -2.72965500 4.14594000

H 4.89919700 -2.01833000 3.53821700

H 4.38833700 -2.40580600 5.18999000

H 4.82090300 -3.70880100 4.07454700

C -10.74833400 0.49735500 1.64528400

H -10.58776200 0.88819600 2.65373800

H -11.44008200 -0.34520800 1.66830000

H -11.14588700 1.30175300 1.02047200

O -9.52694200 -0.01558000 1.09795700

H 4.36417200 1.25807200 0.33419200

**a-6**

SCF energy in solvent model (SMD): -3308.05825691 a.u.

Fe 2.24435700 0.19748500 -0.89590000

Cl 3.30239000 1.61970800 0.46327400

Cl 0.45701500 1.26245100 -1.72966200

Cl 3.58564600 -0.52913900 -2.50361900

Cl 1.45995400 -1.52285000 0.33553900

C -3.57484500 0.05790200 1.52029000

C -4.85354400 -0.30857000 1.12802900

C -5.06135800 -1.27915400 0.12672900

C -3.92404700 -1.86398100 -0.46037600

C -2.63531600 -1.50586400 -0.07829500

C -2.45920100 -0.53959500 0.91706300

H -3.42017100 0.80035500 2.29714000

H -5.70520000 0.16340500 1.60789000

H -4.05610800 -2.61407700 -1.23533900

H -1.77076100 -1.96570200 -0.54819000

O -1.22096500 -0.13870900 1.33705800

H -0.51874600 -0.65969200 0.89559400

C -6.38459400 -1.69949400 -0.32238800

H -6.40486300 -2.45968400 -1.10220700

C -7.58404700 -1.26282800 0.10818000

H -7.70209000 -0.50892600 0.87944800

C -8.82177000 -1.81285500 -0.47314100

O -8.89414000 -2.66133400 -1.34361700

O 5.69495500 -1.76024100 -0.14305000

C 5.70298600 -3.11481700 0.01057200

C 6.46137700 -3.63838200 1.06583200

C 4.99712900 -3.97985700 -0.83592400

C 6.50923900 -5.01580400 1.26835500

H 7.00286400 -2.95168000 1.70877000

C 5.05399900 -5.35746700 -0.62091300

H 4.40771300 -3.57241000 -1.65318800

C 5.80764900 -5.88566500 0.42857800

H 7.10133600 -5.41260700 2.08893300

H 4.50186300 -6.01971700 -1.28242700

H 5.84848500 -6.95854700 0.59036100

C 1.49996800 3.54060900 3.24440600

C 1.23398100 4.47834300 2.29214600

C 0.24923700 2.59616600 1.68886900

N 0.86857400 2.37567400 2.85459300

H 2.08106900 3.59975200 4.15042800

H 1.53295700 5.51097100 2.21915900

H -0.31870600 1.85478700 1.14220600

N 0.44876000 3.86946000 1.33034400

C 0.98635400 1.06812800 3.51197500

H 0.94687900 1.21156800 4.59265000

H 0.15745300 0.43982300 3.18732900

H 1.93284100 0.60652000 3.22162600

C -0.02747900 4.48657900 0.07507800

H -0.64411400 3.73602100 -0.42305600

H -0.66535500 5.33506700 0.34382800

C 1.12559800 4.91611100 -0.83598900

H 1.73670800 5.67480000 -0.32927700

H 1.76963600 4.04964100 -1.01743700

C 0.60855800 5.47596700 -2.16821800

H 0.01275500 4.70433200 -2.67138000

H -0.06855000 6.32010900 -1.97709500

C 1.74491300 5.92577300 -3.09168600

H 2.41397100 5.09139600 -3.32613900

H 1.35218200 6.31445100 -4.03593900

H 2.34552700 6.71694400 -2.62889500

C -11.17428900 -1.71137200 -0.40227800

H -11.26372000 -1.51697200 -1.47450800

H -11.93640800 -1.16112200 0.15025800

H -11.28113600 -2.78643200 -0.23413800

O -9.91527400 -1.24128100 0.09656700

H 5.15236600 -1.51249900 -0.90862300

**MPC+cat+phenol**

SCF energy in solvent model (SMD): -3308.06488087 a.u.

C -2.92444200 3.87091700 0.27652700

C -2.40061500 2.59134000 0.32163300

C -2.62350200 1.67710100 -0.72656600

C -3.37325600 2.11029000 -1.83110100

C -3.90942600 3.39058000 -1.88387100

C -3.68769900 4.27412500 -0.82450500

H -2.74210000 4.57930100 1.07513400

H -1.78560000 2.30772500 1.16618200

H -3.54910800 1.42579000 -2.65394400

H -4.49342000 3.70597800 -2.74335600

O -4.18053500 5.54675000 -0.80964700

H -4.67111100 5.72114100 -1.61979900

C -2.10169200 0.32283800 -0.71300000

H -2.11762600 -0.21118900 -1.65845300

C -1.62783700 -0.34685400 0.35495100

H -1.62411900 0.09400000 1.33955400

C -1.12247200 -1.70345700 0.17340700

O -0.79652000 -2.18393600 -0.90060900

O -3.12907100 -4.26193100 -0.06054600

C -3.91255800 -3.13897400 -0.17923000

C -4.38241700 -2.44999500 0.94119400

C -4.24731000 -2.69771800 -1.45870800

C -5.15572100 -1.30230000 0.77863600

H -4.14107300 -2.82016000 1.93139500

C -5.03056500 -1.55655000 -1.61182100

H -3.86272800 -3.24161000 -2.31251400

C -5.48000800 -0.84725800 -0.49740000

H -5.50303300 -0.76262200 1.65255900

H -5.27948600 -1.21137600 -2.60961100

H -6.06914200 0.05322500 -0.62258900

C 3.84279200 -3.52425800 -0.96567400

C 4.37953800 -2.44406900 -1.59250800

C 2.21277200 -2.08409700 -1.34884000

N 2.48884200 -3.28717700 -0.83369200

H 4.29944600 -4.42790800 -0.60186100

H 5.39194400 -2.22902800 -1.88374000

H 1.23788100 -1.62068300 -1.35117800

N 3.34404600 -1.55966000 -1.82993700

C 1.54910400 -4.13967500 -0.09611000

H 1.77129200 -5.18118600 -0.32614200

H 1.66396900 -3.94563300 0.97035800

H 0.53524500 -3.88471000 -0.39391900

C 3.48025100 -0.23214000 -2.46159200

H 3.82762400 -0.38817600 -3.48645500

H 2.48153800 0.20047900 -2.49462100

C 4.42648100 0.67858800 -1.68222300

H 4.09885500 0.72878300 -0.64148700

H 5.43712800 0.25288400 -1.68136700

C 4.45947400 2.08874400 -2.27987800

H 4.72606100 2.03282600 -3.34337700

H 3.45210700 2.51294700 -2.22486400

C 5.44133500 3.00477700 -1.54471800

H 6.46364600 2.61661400 -1.60205300

H 5.44005200 4.00836900 -1.97776600

H 5.17125500 3.09017100 -0.48988100

Fe 1.79680500 1.03904100 1.49454500

Cl 3.68497200 2.09578900 1.94049100

Cl 1.14637800 1.51609500 -0.59015600

Cl 2.16489400 -1.17835500 1.62399900

Cl 0.18160500 1.54736000 2.94262300

C -1.21171300 -2.02156300 2.59880800

H -0.45523200 -1.27071600 2.82453700

H -1.07599500 -2.88314200 3.25014000

H -2.21426900 -1.61201000 2.73238800

O -1.03715600 -2.53033800 1.25760900

H -2.44139400 -4.08913700 0.59676700

**b-1**

SCF energy in gas phase: -3192.27120587 a.u.

C 2.97630800 5.57012200 -0.38804500

C 3.38608500 4.24541700 -0.32853800

C 2.44407900 3.19463600 -0.20724800

C 1.07437200 3.53478400 -0.14883400

C 0.65267000 4.86012000 -0.20747900

C 1.60660000 5.87828700 -0.32761900

H 3.68811400 6.38134300 -0.48220300

H 4.44585800 4.01966700 -0.38016800

H 0.33413600 2.74913400 -0.04287700

H -0.40819400 5.08818200 -0.15999200

O 1.26713800 7.21894500 -0.39336000

H 0.30049200 7.35263900 -0.34503100

C 2.81055100 1.79038800 -0.14509100

H 1.97416400 1.09530500 -0.09126000

C 4.05543300 1.24983300 -0.14427400

H 4.95869600 1.84598600 -0.17836200

C 4.24064000 -0.18934000 -0.08989400

O 3.36326000 -1.07149600 -0.07707900

O 5.60786300 -0.49513200 -0.04935500

C 6.09405300 -1.82371200 -0.03224000

C 5.62487300 -2.80980100 -0.90513100

C 7.14057200 -2.07731400 0.85711600

C 6.22188200 -4.07524000 -0.87338500

H 4.81307800 -2.59463200 -1.58637300

C 7.73001500 -3.34507100 0.87596700

H 7.47972300 -1.28293800 1.51141600

C 7.27110200 -4.34787500 0.01275500

H 5.86770700 -4.84732400 -1.54911700

H 8.54563000 -3.54690400 1.56253700

H 7.72951000 -5.33119000 0.02769800

C -0.74587800 -4.22070700 -0.39571900

C -2.07468600 -4.51057000 -0.24973700

C -1.71088000 -2.49729900 0.63612700

N -0.53807400 -2.96074100 0.16342700

H 0.05478000 -4.78604300 -0.83876900

H -2.64183400 -5.37557300 -0.54393000

H -1.84915500 -1.53949400 1.11215700

N -2.66197900 -3.42313800 0.39862600

C 0.74065400 -2.21905600 0.17523500

H 1.52742800 -2.82829500 0.61849200

H 1.02353100 -1.94801300 -0.84146400

H 0.61449500 -1.30891100 0.75882400

C -4.10817600 -3.26914700 0.70038600

H -4.41747300 -4.14272200 1.28429200

H -4.21100400 -2.37961800 1.32683200

C -4.95394100 -3.11743400 -0.57270000

H -4.60179400 -2.24014500 -1.12872800

H -4.81342800 -3.99484500 -1.22086600

C -6.44918200 -2.95669600 -0.23756400

H -6.78925500 -3.81961300 0.35485300

H -6.57895200 -2.06684300 0.39189300

C -7.31722200 -2.82369000 -1.49847800

H -7.22601300 -3.70881400 -2.14075000

H -8.37421100 -2.70747600 -1.23598300

H -7.02065700 -1.94792200 -2.08737900

Fe -2.95020400 1.30865700 0.26961200

Cl -4.79852600 0.44407600 1.23834400

Cl -1.21132400 0.84258600 1.68202400

Cl -2.53907500 0.09763700 -1.60220500

Cl -3.06786000 3.50186600 -0.13671100

**b-ts1**

SCF energy in gas phase: -3192.26885871 a.u.

C -7.58898000 -0.02822300 0.56123100

C -6.31919900 0.48131700 0.32502800

C -5.23661700 -0.37104000 0.00087600

C -5.48552500 -1.75866800 -0.07574700

C -6.75646600 -2.27970000 0.16031500

C -7.80863200 -1.41222900 0.47908600

H -8.42307600 0.61685200 0.80974200

H -6.16191600 1.55237100 0.39188000

H -4.66713600 -2.42804100 -0.32229200

H -6.92661800 -3.35104000 0.09738900

O -9.09966200 -1.85368300 0.72703700

H -9.17411500 -2.82473100 0.65333500

C -3.88467100 0.10334800 -0.25570900

H -3.15765500 -0.66737100 -0.50310400

C -3.43793200 1.38290300 -0.22603400

H -4.07279200 2.23063500 0.00026000

C -2.04510300 1.68682400 -0.50237200

O -1.12524600 0.89478300 -0.76782000

O -1.84178500 3.07210800 -0.42814500

C -0.60847700 3.71674800 -0.66154800

C 0.35620700 3.26575100 -1.56989100

C -0.43809500 4.91372100 0.04447300

C 1.50811700 4.04105200 -1.76182800

H 0.23127500 2.32925500 -2.09233500

C 0.71491900 5.67622600 -0.16014400

H -1.21437900 5.23063400 0.73102100

C 1.69388400 5.24196700 -1.06526500

H 2.25759300 3.69685300 -2.46715500

H 0.84329100 6.60951100 0.37892000

H 2.58446700 5.83930100 -1.23374100

C 4.89913300 1.94360900 1.23401300

C 5.62660700 0.79771900 1.07099000

C 3.53240500 0.21275800 1.56757100

N 3.59553400 1.55922800 1.54653000

H 5.18415000 2.97753600 1.15337500

H 6.66240100 0.65153500 0.82184500

H 2.64424900 -0.36809200 1.76117900

N 4.75796900 -0.27262900 1.28670700

C 2.45424300 2.47363200 1.74273500

H 2.63470300 3.10002600 2.61920300

H 2.33075800 3.09196600 0.85207800

H 1.55269000 1.87833200 1.88775800

C 5.09663000 -1.70946600 1.12319400

H 5.97110500 -1.91519900 1.74991100

H 4.24648900 -2.27884600 1.50492000

C 5.35347500 -2.07872100 -0.34536100

H 4.46735200 -1.81265500 -0.93350800

H 6.19843200 -1.49301400 -0.73667200

C 5.64822900 -3.58261900 -0.50043800

H 6.51139300 -3.85852400 0.12444500

H 4.78744900 -4.15175900 -0.12577300

C 5.92262300 -3.97394800 -1.96093900

H 6.79074100 -3.43525200 -2.36171600

H 6.12396800 -5.04712400 -2.04876800

H 5.05929000 -3.74178200 -2.59505500

Fe 0.33447800 -1.36715600 -0.20626900

Cl 1.67867700 -3.11350000 0.47767500

Cl -0.05265900 -0.46440100 1.88016800

Cl 1.86408600 -0.18484600 -1.42319000

Cl -1.31964300 -2.42872000 -1.29822900

**b-2**

SCF energy in gas phase: -3192.26957744 a.u.

C -7.48876900 -0.08721900 0.57929000

C -6.22093000 0.41742500 0.32657200

C -5.14076200 -0.44220500 0.01097200

C -5.39045300 -1.83169300 -0.04038200

C -6.65947200 -2.34737900 0.21268000

C -7.70908900 -1.47299400 0.52294200

H -8.32128800 0.56198400 0.82197200

H -6.06283700 1.48931200 0.37440700

H -4.57370900 -2.50513200 -0.28103000

H -6.83096800 -3.41936300 0.16985300

O -8.99729300 -1.90825300 0.78693600

H -9.07461600 -2.88046000 0.73326900

C -3.79110000 0.02274800 -0.25968500

H -3.07034600 -0.75568700 -0.49813400

C -3.34403100 1.30498300 -0.25816100

H -3.98298100 2.15442800 -0.05137100

C -1.96141200 1.62282000 -0.53514400

O -1.01607900 0.83393300 -0.77127300

O -1.77581700 2.99565100 -0.51738200

C -0.54319900 3.66391500 -0.71877400

C 0.43630900 3.23739900 -1.61886800

C -0.40608000 4.85176700 0.00690800

C 1.58173900 4.02889300 -1.77962800

H 0.33294600 2.30702500 -2.15672900

C 0.74026000 5.63132200 -0.16775200

H -1.19687000 5.14715600 0.68626100

C 1.73908000 5.22134700 -1.06247300

H 2.34865700 3.70225600 -2.47398500

H 0.84863400 6.55760500 0.38721600

H 2.62544500 5.83119100 -1.20602200

C 5.08734400 1.59173800 1.37456400

C 5.71203300 0.39337400 1.17044100

C 3.55672900 -0.02033700 1.57556200

N 3.74403100 1.31417100 1.62918900

H 5.46848100 2.59737300 1.36057000

H 6.73833500 0.16347500 0.94591800

H 2.61859500 -0.53791500 1.70572800

N 4.74277800 -0.60105500 1.30439500

C 2.68471500 2.32090200 1.82633200

H 2.95280400 2.97012500 2.66258900

H 2.56611800 2.90635300 0.91217300

H 1.74759100 1.80542400 2.03815300

C 4.95440000 -2.05194900 1.06752000

H 5.84550500 -2.34820500 1.63120500

H 4.08604600 -2.56897300 1.48029800

C 5.09328300 -2.37661300 -0.42720300

H 4.20305800 -2.00624700 -0.94902800

H 5.96488500 -1.85075000 -0.84433300

C 5.23719100 -3.89237000 -0.65937500

H 6.10509500 -4.27448400 -0.10022100

H 4.34953200 -4.39659300 -0.25514000

C 5.38821500 -4.24061000 -2.14846500

H 6.27895500 -3.76666400 -2.58028800

H 5.48098500 -5.32274700 -2.29179500

H 4.51561700 -3.90008400 -2.71755500

Fe 0.21715500 -0.96163600 -0.21965200

Cl 1.48668400 -2.75246500 0.62458000

Cl -0.09496600 -0.07923700 1.93311500

Cl 1.95328600 0.01060100 -1.39244700

Cl -1.22970100 -2.25489300 -1.41828400

**b-3**

SCF energy in gas phase: -3192.26676403 a.u.

C -7.27877600 -1.03231300 -0.00955800

C -6.11507100 -0.27705500 0.02877700

C -4.84292800 -0.89551600 0.09305000

C -4.79152200 -2.30670600 0.11595500

C -5.95412200 -3.07333900 0.07719800

C -7.19895500 -2.43396200 0.01443900

H -8.25614700 -0.56809300 -0.06097100

H -6.18992900 0.80456400 0.00544600

H -3.82472400 -2.79805300 0.16415900

H -5.89308900 -4.15800700 0.09354000

O -8.39871400 -3.12544000 -0.02634400

H -8.26537200 -4.09300300 -0.01944000

C -3.59004000 -0.15975200 0.13396600

H -2.69397400 -0.77239900 0.18637700

C -3.42513900 1.18756600 0.12840100

H -4.25327700 1.88448900 0.09260300

C -2.11201100 1.79210400 0.15869500

O -0.99669500 1.21586900 0.18065600

O -2.20648100 3.16774100 0.18594300

C -1.07394900 4.02745900 0.09634700

C -0.14094800 3.90600600 -0.93382100

C -1.01115400 5.05476600 1.03745700

C 0.89275100 4.84635600 -1.00629500

H -0.20979000 3.09889900 -1.65233700

C 0.02513600 5.98997100 0.94798000

H -1.76653800 5.11422300 1.81224400

C 0.97887800 5.88579800 -0.07214100

H 1.63196500 4.75862200 -1.79502900

H 0.08424800 6.79615700 1.67198500

H 1.78367300 6.61055000 -0.13933100

C 4.54931900 -0.90394100 3.27439600

C 5.32930300 -1.42893100 2.28274000

C 3.19449200 -1.72794900 1.70272900

N 3.22092900 -1.10310800 2.89752500

H 4.81365200 -0.41450100 4.19494300

H 6.39861700 -1.48266000 2.18205100

H 2.31024700 -1.99134300 1.14247000

N 4.46710100 -1.94173400 1.31259400

C 2.04084600 -0.59392100 3.61819000

H 2.11707300 -0.86518000 4.67283400

H 1.98985200 0.49091300 3.50206900

H 1.14407400 -1.03133400 3.17901700

C 4.87935900 -2.51168200 0.00517700

H 5.64829900 -3.26605400 0.20322600

H 4.00004700 -3.00243700 -0.41614800

C 5.38247100 -1.42649900 -0.95857800

H 4.60068900 -0.66495900 -1.06458900

H 6.27017000 -0.93313500 -0.53572000

C 5.72116500 -2.01740600 -2.33984800

H 6.46688400 -2.81961000 -2.22901200

H 4.81606400 -2.47782400 -2.75728900

C 6.24992000 -0.95357300 -3.31456300

H 7.16653100 -0.48512900 -2.93395700

H 6.47860400 -1.39408200 -4.29116100

H 5.50616300 -0.16335800 -3.46914300

Fe 0.36928100 -0.29271300 -0.61989900

Cl 1.68461900 -2.04171500 -1.48949300

Cl -0.40139700 -1.67556500 1.13995200

Cl 2.13895900 0.99069300 0.12116700

Cl -0.64162600 0.19699400 -2.61271400

**b-ts2**

SCF energy in gas phase: -3192.25328401 a.u.

C -6.55536600 -2.45378100 0.19695800

C -5.56037300 -1.50595400 0.38542700

C -4.19635000 -1.82461400 0.17182000

C -3.87714900 -3.13826600 -0.24021800

C -4.86918600 -4.09589500 -0.43307300

C -6.20993300 -3.75205700 -0.21372200

H -7.60094000 -2.22025800 0.35655900

H -5.83968300 -0.50644200 0.69978600

H -2.83731700 -3.39947900 -0.40957900

H -4.60390400 -5.09990400 -0.75208000

O -7.25221700 -4.64619800 -0.38251300

H -6.94158100 -5.52440500 -0.67646900

C -3.11221400 -0.87822400 0.34889600

H -2.11787900 -1.27136300 0.15891100

C -3.20300100 0.42475200 0.73470000

H -4.14880500 0.90004300 0.96486700

C -2.03451700 1.25826000 0.86687900

O -0.84417700 0.94584200 0.65418700

O -2.29732900 2.54446400 1.36147200

C -2.69147900 3.59210700 0.49309400

C -2.53578200 3.51293000 -0.89382300

C -3.22917700 4.72429700 1.10935400

C -2.94500600 4.60145900 -1.67420100

H -2.08865600 2.64111400 -1.35957500

C -3.62654200 5.80287900 0.31475200

H -3.32366000 4.74431900 2.18852200

C -3.48985300 5.74458000 -1.07884400

H -2.82594900 4.55100500 -2.75154300

H -4.04348100 6.68758800 0.78523900

H -3.80012200 6.58359100 -1.69263600

C 5.18234000 0.08012600 3.05337600

C 5.98381000 -0.27483900 2.00475400

C 3.92736600 -1.03062400 1.57950600

N 3.90435500 -0.40345900 2.77287700

H 5.40045300 0.62536700 3.95445100

H 7.02968900 -0.09881800 1.82701000

H 3.08182600 -1.48245200 1.08418500

N 5.18448600 -0.97053400 1.09680200

C 2.69437800 -0.14364900 3.57359300

H 2.91303800 -0.33085300 4.62636500

H 2.37902700 0.89087300 3.42079600

H 1.89600000 -0.80158200 3.23017500

C 5.61256200 -1.45500100 -0.23939400

H 6.53635300 -2.02706500 -0.10193800

H 4.82948700 -2.12693100 -0.59621000

C 5.80191900 -0.30042800 -1.23447700

H 4.86726600 0.26992300 -1.28762900

H 6.58620900 0.38077000 -0.87282200

C 6.16997200 -0.82338000 -2.63558100

H 7.08365200 -1.43454800 -2.57659300

H 5.36634300 -1.48348800 -2.98787900

C 6.37451000 0.31553400 -3.64671400

H 7.18533900 0.98565000 -3.33380400

H 6.62816900 -0.07863700 -4.63688000

H 5.46206000 0.91429500 -3.74752100

Fe 0.71750400 -0.17013000 -0.43774900

Cl 2.28809500 -1.51621900 -1.54820500

Cl 0.40104700 -1.79122700 1.24784500

Cl 2.20510800 1.45955400 0.22056100

Cl -0.65105600 0.12547300 -2.24629200

**b-4**

SCF energy in gas phase: -3192.25735324 a.u.

C -6.58397600 -2.30352600 -0.26110800

C -5.54756600 -1.43212100 0.04196800

C -4.19460200 -1.83176800 -0.08477300

C -3.92930300 -3.14624300 -0.52943200

C -4.96301000 -4.02734600 -0.83803000

C -6.29187000 -3.60419600 -0.70318800

H -7.62169800 -2.00715600 -0.16812500

H -5.78624400 -0.42972000 0.38028200

H -2.89782300 -3.46726200 -0.63679400

H -4.73838400 -5.03291800 -1.18266500

O -7.37364900 -4.41990000 -0.98831600

H -7.09707000 -5.30305200 -1.30072200

C -3.06677400 -0.96592800 0.20973500

H -2.08326500 -1.41940600 0.09490600

C -3.09972000 0.33555100 0.60265200

H -4.02567200 0.88108100 0.73130200

C -1.85713100 1.04054500 0.84807000

O -0.72119400 0.51183900 0.82879500

O -1.90240400 2.38146700 1.17886000

C -3.02324400 3.20831200 0.89603900

C -3.34520700 3.50271600 -0.43112700

C -3.70964800 3.77525600 1.96945500

C -4.40549600 4.38073900 -0.68138800

H -2.76316400 3.06655600 -1.23576800

C -4.76479300 4.65568600 1.70295500

H -3.41025300 3.53525000 2.98293100

C -5.11606400 4.95582200 0.38065400

H -4.66747700 4.62175400 -1.70622100

H -5.30740600 5.10687500 2.52713900

H -5.93348700 5.64009000 0.17875100

C 5.20099900 -1.67285200 2.86277300

C 6.08633200 -1.30734900 1.88805900

C 4.11011400 -1.66866600 0.91508000

N 3.97453600 -1.89841000 2.23683000

H 5.32900900 -1.78819000 3.92443400

H 7.12841300 -1.04773500 1.94427100

H 3.33035500 -1.73039600 0.17154000

N 5.38908900 -1.31457700 0.67920000

C 2.70540300 -2.19972200 2.92168400

H 2.85891800 -3.02444900 3.62029300

H 2.36125200 -1.30684900 3.44813700

H 1.95754500 -2.47026700 2.17570900

C 5.92669700 -0.87381100 -0.63253400

H 6.88732700 -1.37873200 -0.77998700

H 5.22367200 -1.21857400 -1.39322800

C 6.06919600 0.65381800 -0.70752100

H 5.09418600 1.10530900 -0.48965900

H 6.77435800 1.00117900 0.06204900

C 6.55043100 1.10409300 -2.09936800

H 7.50598600 0.61322200 -2.33994300

H 5.82169200 0.76955900 -2.84945300

C 6.71587900 2.62910000 -2.19259900

H 7.45304200 2.99504600 -1.46650800

H 7.05170400 2.92800800 -3.19164600

H 5.76515000 3.13630800 -1.99235400

Fe 0.93386900 0.12138900 -0.52961400

Cl 2.68028000 -0.39806200 -2.02664200

Cl 0.55872800 -2.15535800 -0.03991200

Cl 2.32504300 1.09705400 1.04440200

Cl -0.32043500 1.25185800 -2.08219800

**b-5**

SCF energy in gas phase: -3307.98674592 a.u.

C -4.90812300 2.88094600 -0.15728500

C -4.13804000 1.74110400 -0.29433800

C -2.81831000 1.79974300 -0.78705800

C -2.30542500 3.05473300 -1.14612600

C -3.07019300 4.20661300 -1.01104000

C -4.37535900 4.12177800 -0.51903200

H -5.92688200 2.83521900 0.20703300

H -4.58501300 0.78514500 -0.05076200

H -1.28720200 3.12499700 -1.51244300

H -2.65157900 5.17177000 -1.28029600

O -5.18980500 5.20808100 -0.38182400

H -4.71553500 6.00905200 -0.63043500

C -2.01438700 0.60003000 -0.97273500

H -1.15758800 0.66668700 -1.63533300

C -2.31164600 -0.58075700 -0.40204400

H -3.13452300 -0.63666400 0.29326200

C -1.67523000 -1.84207200 -0.74903300

O -0.61522900 -2.03589900 -1.28459500

O -2.45064100 -2.96716200 -0.40427000

C -3.82903300 -2.78743700 -0.27972200

C -4.42354500 -2.88437200 0.97428200

C -4.58394500 -2.47035000 -1.40691900

C -5.78673900 -2.62045700 1.10533700

H -3.81779800 -3.13147300 1.83821400

C -5.94326500 -2.20529500 -1.26664600

H -4.09398700 -2.40097500 -2.37057900

C -6.54647700 -2.26950300 -0.01000600

H -6.25079500 -2.68640700 2.08263000

H -6.53071100 -1.94361900 -2.13897700

H -7.60452200 -2.06068600 0.09842600

C 3.98086400 -3.81313200 -0.23982300

C 4.82707500 -2.86396600 -0.72168000

C 2.75663200 -2.15072200 -1.02530100

N 2.69530000 -3.35475000 -0.44806300

H 4.17561900 -4.76197800 0.22803600

H 5.90107800 -2.82831500 -0.75738800

H 1.90565700 -1.53148000 -1.26830300

N 4.04443000 -1.83702100 -1.21384200

C 1.46717600 -3.99620900 0.04087000

H 1.64718500 -5.06589100 0.11662500

H 1.19886000 -3.57316300 1.00828300

H 0.65839000 -3.79276900 -0.65512000

C 4.56124700 -0.56088600 -1.74693200

H 5.20143700 -0.79693200 -2.60091000

H 3.70158400 0.00271800 -2.10478900

C 5.31894200 0.23497700 -0.68363200

H 4.68234400 0.35256500 0.19737400

H 6.20611200 -0.32679600 -0.36684900

C 5.73498200 1.61229300 -1.21054100

H 6.24312100 1.50083700 -2.17742100

H 4.83147200 2.20323300 -1.39039000

C 6.64778400 2.35453600 -0.23170300

H 7.57951000 1.80328700 -0.06548800

H 6.90759200 3.34578600 -0.61175400

H 6.15097000 2.48581100 0.73182900

Fe 1.69988400 1.37374800 0.83856500

Cl 3.32722800 2.81530200 1.26426900

Cl 1.50358300 1.19456700 -1.40737300

Cl 2.27487400 -0.63061100 1.66401000

Cl -0.22579000 2.02601700 1.69909500

C -1.09203400 -1.56614200 2.81629200

H -0.26195100 -0.86736800 2.91859500

H -1.33906100 -1.98472800 3.81143200

H -1.95589100 -1.01395900 2.44471900

O -0.67083200 -2.58409500 1.90454400

H -1.43688800 -3.08875300 1.60944300

**b-ts3**

SCF energy in gas phase: -3307.91945801 a.u.

C -5.28276600 2.80012900 -0.30683300

C -4.40906600 1.72079400 -0.30699100

C -3.04515700 1.89126100 -0.64305600

C -2.59459400 3.18124400 -0.99418000

C -3.46300400 4.27028000 -0.98664800

C -4.80742800 4.07814300 -0.64006600

H -6.33130000 2.68389000 -0.06160600

H -4.78910000 0.73167000 -0.07243900

H -1.54878700 3.32706600 -1.24409600

H -3.09833800 5.26081700 -1.24320000

O -5.73295400 5.10944300 -0.61520100

H -5.33198100 5.96984300 -0.84463000

C -2.08974700 0.79620800 -0.63367000

H -1.15198700 0.97077100 -1.15683000

C -2.25138700 -0.39012400 0.00297100

H -3.12020100 -0.61902800 0.60355800

C -1.22700200 -1.39264900 -0.09048800

O -0.27581400 -1.59463000 -0.80372400

O -2.26731100 -3.13560600 0.15775900

C -3.62475300 -3.09475700 -0.02930000

C -4.50627300 -3.03571100 1.06829300

C -4.14878600 -3.05496200 -1.33550900

C -5.88686600 -2.94749700 0.85901700

H -4.09799100 -3.07192200 2.07450600

C -5.52745900 -2.96107600 -1.53524300

H -3.45635800 -3.09146500 -2.16906600

C -6.40487900 -2.90512400 -0.44163400

H -6.55725300 -2.90828100 1.71255300

H -5.92115900 -2.92749600 -2.54672700

H -7.47543600 -2.83182900 -0.60219600

C 3.70989000 -4.04109200 -0.18184300

C 4.61229500 -3.17697000 -0.73637400

C 2.58744100 -2.24193400 -0.88121800

N 2.45314000 -3.44475400 -0.28467400

H 3.84384900 -5.00816000 0.26887000

H 5.67814800 -3.24894800 -0.85707900

H 1.78795900 -1.54410000 -1.06494300

N 3.89249500 -2.06440900 -1.17127200

C 1.19919700 -3.99423600 0.26947900

H 1.39124000 -5.01904600 0.58821600

H 0.87635100 -3.37844100 1.10973100

H 0.41146900 -3.97432800 -0.48244800

C 4.48687500 -0.83985000 -1.75715800

H 5.08168000 -1.14300900 -2.62549600

H 3.66017900 -0.21711600 -2.10271300

C 5.34258700 -0.07345500 -0.73898100

H 4.73624900 0.15062600 0.14626700

H 6.17875100 -0.70684400 -0.40791100

C 5.88566400 1.23555500 -1.33972600

H 6.38688900 1.02412400 -2.29690700

H 5.04007000 1.90073800 -1.55756700

C 6.86137000 1.94367100 -0.38798000

H 7.73165700 1.31050200 -0.17132800

H 7.22474600 2.88067200 -0.82377800

H 6.36527600 2.18461900 0.55778600

Fe 1.85977500 1.60084800 0.72704900

Cl 3.60057200 2.97537000 0.98743200

Cl 1.54478000 1.25167600 -1.50102000

Cl 2.31573300 -0.38861900 1.71105400

Cl -0.02896500 2.43691100 1.59596500

C -1.11236200 -1.11209900 2.75101400

H -0.57875600 -0.18250400 2.55651500

H -0.69002800 -1.60233600 3.63101400

H -2.17815600 -0.91109900 2.90003100

O -0.89664000 -1.99579200 1.60426300

H -1.62832100 -2.74986000 1.33390400

**MPC+cat+phenol**

SCF energy in gas phase: -3308.00468983 a.u.

C -2.92444200 3.87091700 0.27652700

C -2.40061500 2.59134000 0.32163300

C -2.62350200 1.67710100 -0.72656600

C -3.37325600 2.11029000 -1.83110100

C -3.90942600 3.39058000 -1.88387100

C -3.68769900 4.27412500 -0.82450500

H -2.74210000 4.57930100 1.07513400

H -1.78560000 2.30772500 1.16618200

H -3.54910800 1.42579000 -2.65394400

H -4.49342000 3.70597800 -2.74335600

O -4.18053500 5.54675000 -0.80964700

H -4.67111100 5.72114100 -1.61979900

C -2.10169200 0.32283800 -0.71300000

H -2.11762600 -0.21118900 -1.65845300

C -1.62783700 -0.34685400 0.35495100

H -1.62411900 0.09400000 1.33955400

C -1.12247200 -1.70345700 0.17340700

O -0.79652000 -2.18393600 -0.90060900

O -3.12907100 -4.26193100 -0.06054600

C -3.91255800 -3.13897400 -0.17923000

C -4.38241700 -2.44999500 0.94119400

C -4.24731000 -2.69771800 -1.45870800

C -5.15572100 -1.30230000 0.77863600

H -4.14107300 -2.82016000 1.93139500

C -5.03056500 -1.55655000 -1.61182100

H -3.86272800 -3.24161000 -2.31251400

C -5.48000800 -0.84725800 -0.49740000

H -5.50303300 -0.76262200 1.65255900

H -5.27948600 -1.21137600 -2.60961100

H -6.06914200 0.05322500 -0.62258900

C 3.84279200 -3.52425800 -0.96567400

C 4.37953800 -2.44406900 -1.59250800

C 2.21277200 -2.08409700 -1.34884000

N 2.48884200 -3.28717700 -0.83369200

H 4.29944600 -4.42790800 -0.60186100

H 5.39194400 -2.22902800 -1.88374000

H 1.23788100 -1.62068300 -1.35117800

N 3.34404600 -1.55966000 -1.82993700

C 1.54910400 -4.13967500 -0.09611000

H 1.77129200 -5.18118600 -0.32614200

H 1.66396900 -3.94563300 0.97035800

H 0.53524500 -3.88471000 -0.39391900

C 3.48025100 -0.23214000 -2.46159200

H 3.82762400 -0.38817600 -3.48645500

H 2.48153800 0.20047900 -2.49462100

C 4.42648100 0.67858800 -1.68222300

H 4.09885500 0.72878300 -0.64148700

H 5.43712800 0.25288400 -1.68136700

C 4.45947400 2.08874400 -2.27987800

H 4.72606100 2.03282600 -3.34337700

H 3.45210700 2.51294700 -2.22486400

C 5.44133500 3.00477700 -1.54471800

H 6.46364600 2.61661400 -1.60205300

H 5.44005200 4.00836900 -1.97776600

H 5.17125500 3.09017100 -0.48988100

Fe 1.79680500 1.03904100 1.49454500

Cl 3.68497200 2.09578900 1.94049100

Cl 1.14637800 1.51609500 -0.59015600

Cl 2.16489400 -1.17835500 1.62399900

Cl 0.18160500 1.54736000 2.94262300

C -1.21171300 -2.02156300 2.59880800

H -0.45523200 -1.27071600 2.82453700

H -1.07599500 -2.88314200 3.25014000

H -2.21426900 -1.61201000 2.73238800

O -1.03715600 -2.53033800 1.25760900

H -2.44139400 -4.08913700 0.59676700

**b-1**

SCF energy in solvent model (SMD): -3192.34227341 a.u.

C 2.51289200 5.58791100 -0.67701900

C 2.98408900 4.29175100 -0.53163000

C 2.10282700 3.21624000 -0.27687300

C 0.72714200 3.50271500 -0.17502700

C 0.24179300 4.79773900 -0.31983200

C 1.13545200 5.84432000 -0.57148600

H 3.18669700 6.41571000 -0.87207500

H 4.05128500 4.11209700 -0.61689900

H 0.02578300 2.70233200 0.03065900

H -0.82567300 4.98195300 -0.23391400

O 0.73159500 7.13912000 -0.72408000

H -0.22851800 7.20041100 -0.62896500

C 2.53880800 1.83766900 -0.12257500

H 1.74756700 1.11600900 0.07539400

C 3.80298600 1.35694500 -0.19048900

H 4.66851300 1.98754100 -0.36084100

C 4.06006100 -0.07256200 -0.03412400

O 3.23713800 -0.96124600 0.11102700

O 5.41623700 -0.32150300 -0.07627700

C 5.86198200 -1.64021000 0.00490000

C 5.57152200 -2.55595400 -1.00689700

C 6.66806800 -1.98635700 1.08689800

C 6.10015300 -3.84596000 -0.92474700

H 4.94188600 -2.25521600 -1.83695400

C 7.19486400 -3.27958700 1.15820300

H 6.87383700 -1.24520900 1.85221700

C 6.91148300 -4.21168300 0.15550200

H 5.88194600 -4.56493700 -1.70941100

H 7.82635700 -3.55564000 1.99764100

H 7.32294100 -5.21509100 0.21218900

C -0.83181300 -3.76477200 -1.16704700

C -2.15053600 -4.10825900 -1.09061100

C -1.72803400 -2.64318000 0.50975000

N -0.58703200 -2.85822400 -0.15486700

H -0.05434100 -4.08138100 -1.84349800

H -2.74337100 -4.78303600 -1.68642400

H -1.85895200 -1.94105400 1.32007800

N -2.69060800 -3.40296800 -0.03152500

C 0.67051500 -2.11844200 0.03127900

H 1.50794300 -2.76257200 -0.22890800

H 0.65910600 -1.23563700 -0.60773900

H 0.76109100 -1.80170400 1.06700800

C -4.11891800 -3.37883000 0.33732700

H -4.44410800 -4.41673800 0.46169000

H -4.18873600 -2.87500000 1.30248200

C -4.95420300 -2.64085800 -0.71249600

H -4.51033800 -1.65386000 -0.87588500

H -4.90534300 -3.18102300 -1.66787200

C -6.41344500 -2.49063700 -0.26775000

H -6.85207000 -3.48215200 -0.08515800

H -6.43632400 -1.95016200 0.68649500

C -7.25359800 -1.73314000 -1.30212700

H -7.26142300 -2.25457500 -2.26688200

H -8.29114800 -1.62853300 -0.96866600

H -6.85021000 -0.72803200 -1.46676000

Fe -2.48291100 0.94667200 0.54785200

Cl -4.16050200 -0.11464700 1.57956700

Cl -0.66124400 0.58782600 1.79810000

Cl -2.11128200 -0.05337000 -1.42505700

Cl -2.91341500 3.09020500 0.28547500

**b-ts1**

SCF energy in solvent model (SMD): -3192.33114186 a.u.

C -7.58898000 -0.02822300 0.56123100

C -6.31919900 0.48131700 0.32502800

C -5.23661700 -0.37104000 0.00087600

C -5.48552500 -1.75866800 -0.07574700

C -6.75646600 -2.27970000 0.16031500

C -7.80863200 -1.41222900 0.47908600

H -8.42307600 0.61685200 0.80974200

H -6.16191600 1.55237100 0.39188000

H -4.66713600 -2.42804100 -0.32229200

H -6.92661800 -3.35104000 0.09738900

O -9.09966200 -1.85368300 0.72703700

H -9.17411500 -2.82473100 0.65333500

C -3.88467100 0.10334800 -0.25570900

H -3.15765500 -0.66737100 -0.50310400

C -3.43793200 1.38290300 -0.22603400

H -4.07279200 2.23063500 0.00026000

C -2.04510300 1.68682400 -0.50237200

O -1.12524600 0.89478300 -0.76782000

O -1.84178500 3.07210800 -0.42814500

C -0.60847700 3.71674800 -0.66154800

C 0.35620700 3.26575100 -1.56989100

C -0.43809500 4.91372100 0.04447300

C 1.50811700 4.04105200 -1.76182800

H 0.23127500 2.32925500 -2.09233500

C 0.71491900 5.67622600 -0.16014400

H -1.21437900 5.23063400 0.73102100

C 1.69388400 5.24196700 -1.06526500

H 2.25759300 3.69685300 -2.46715500

H 0.84329100 6.60951100 0.37892000

H 2.58446700 5.83930100 -1.23374100

C 4.89913300 1.94360900 1.23401300

C 5.62660700 0.79771900 1.07099000

C 3.53240500 0.21275800 1.56757100

N 3.59553400 1.55922800 1.54653000

H 5.18415000 2.97753600 1.15337500

H 6.66240100 0.65153500 0.82184500

H 2.64424900 -0.36809200 1.76117900

N 4.75796900 -0.27262900 1.28670700

C 2.45424300 2.47363200 1.74273500

H 2.63470300 3.10002600 2.61920300

H 2.33075800 3.09196600 0.85207800

H 1.55269000 1.87833200 1.88775800

C 5.09663000 -1.70946600 1.12319400

H 5.97110500 -1.91519900 1.74991100

H 4.24648900 -2.27884600 1.50492000

C 5.35347500 -2.07872100 -0.34536100

H 4.46735200 -1.81265500 -0.93350800

H 6.19843200 -1.49301400 -0.73667200

C 5.64822900 -3.58261900 -0.50043800

H 6.51139300 -3.85852400 0.12444500

H 4.78744900 -4.15175900 -0.12577300

C 5.92262300 -3.97394800 -1.96093900

H 6.79074100 -3.43525200 -2.36171600

H 6.12396800 -5.04712400 -2.04876800

H 5.05929000 -3.74178200 -2.59505500

Fe 0.33447800 -1.36715600 -0.20626900

Cl 1.67867700 -3.11350000 0.47767500

Cl -0.05265900 -0.46440100 1.88016800

Cl 1.86408600 -0.18484600 -1.42319000

Cl -1.31964300 -2.42872000 -1.29822900

**b-2**

SCF energy in solvent model (SMD): -3192.35534544 a.u.

C 5.46188600 -1.35060800 -1.56109800

C 4.19745600 -1.84572800 -1.28412700

C 3.58461300 -1.63247000 -0.02960700

C 4.30279600 -0.90549600 0.93890000

C 5.57037000 -0.39904000 0.67126200

C 6.15089100 -0.61626900 -0.58321900

H 5.93106900 -1.50605900 -2.52682600

H 3.67450100 -2.40174000 -2.05555700

H 3.84085100 -0.70113700 1.89923100

H 6.09755700 0.18055000 1.42541600

O 7.38611400 -0.14168400 -0.91921300

H 7.74563400 0.39315100 -0.19909600

C 2.23853900 -2.08008400 0.28751000

H 1.90595000 -1.89455700 1.30657900

C 1.33676000 -2.62709600 -0.55898100

H 1.53547200 -2.78133700 -1.61301200

C -0.02729000 -2.88453500 -0.11636100

O -0.46985600 -2.83157000 1.02084900

O -0.82537200 -3.18890500 -1.20190400

C -2.20324100 -3.23226800 -1.03452300

C -2.95640400 -2.32861900 -1.78342900

C -2.81318500 -4.16316800 -0.19146700

C -4.35015300 -2.35797300 -1.68639100

H -2.43950000 -1.61129600 -2.41166600

C -4.20770700 -4.17536900 -0.09319400

H -2.20073500 -4.84979600 0.38074100

C -4.97961000 -3.27496900 -0.83723700

H -4.94325900 -1.66459600 -2.27680400

H -4.69106000 -4.89510500 0.56128600

H -6.06294700 -3.29618900 -0.76289200

C -2.94556700 -0.84994200 1.52111400

C -3.57704800 0.11381700 0.79077000

C -2.08724800 1.10932300 2.08282900

N -2.03277300 -0.20539300 2.33301500

H -3.02696500 -1.92444000 1.49760500

H -4.33114900 0.02664100 0.02787500

H -1.43959200 1.85928000 2.51011800

N -3.03393700 1.33017100 1.16064800

C -1.15587800 -0.86491400 3.30751000

H -1.77375400 -1.46675100 3.97760600

H -0.45024900 -1.49741800 2.77112000

H -0.62123000 -0.10251100 3.86981900

C -3.40647400 2.64792500 0.61140100

H -4.44534200 2.84264800 0.90008100

H -2.76556300 3.38166600 1.10285400

C -3.22277500 2.72656300 -0.90466800

H -2.20241700 2.42178100 -1.14887400

H -3.90098000 2.01899100 -1.40073100

C -3.48200500 4.14599400 -1.42296100

H -4.49737700 4.46681100 -1.14879600

H -2.78588800 4.83382300 -0.92633300

C -3.29924900 4.24481800 -2.94169200

H -3.99481300 3.58063300 -3.46891900

H -3.47590000 5.26535100 -3.29687300

H -2.28113200 3.95979100 -3.22865700

Fe 0.99134900 1.70364700 0.15153000

Cl -0.06637400 3.65866100 0.47764000

Cl 1.45003800 0.91967300 2.20831100

Cl -0.45995800 0.33453200 -0.85980600

Cl 2.82845600 1.91604500 -1.02924000

**b-3**

SCF energy in solvent model (SMD): -3192.32998542 a.u.

C -7.27877600 -1.03231300 -0.00955800

C -6.11507100 -0.27705500 0.02877700

C -4.84292800 -0.89551600 0.09305000

C -4.79152200 -2.30670600 0.11595500

C -5.95412200 -3.07333900 0.07719800

C -7.19895500 -2.43396200 0.01443900

H -8.25614700 -0.56809300 -0.06097100

H -6.18992900 0.80456400 0.00544600

H -3.82472400 -2.79805300 0.16415900

H -5.89308900 -4.15800700 0.09354000

O -8.39871400 -3.12544000 -0.02634400

H -8.26537200 -4.09300300 -0.01944000

C -3.59004000 -0.15975200 0.13396600

H -2.69397400 -0.77239900 0.18637700

C -3.42513900 1.18756600 0.12840100

H -4.25327700 1.88448900 0.09260300

C -2.11201100 1.79210400 0.15869500

O -0.99669500 1.21586900 0.18065600

O -2.20648100 3.16774100 0.18594300

C -1.07394900 4.02745900 0.09634700

C -0.14094800 3.90600600 -0.93382100

C -1.01115400 5.05476600 1.03745700

C 0.89275100 4.84635600 -1.00629500

H -0.20979000 3.09889900 -1.65233700

C 0.02513600 5.98997100 0.94798000

H -1.76653800 5.11422300 1.81224400

C 0.97887800 5.88579800 -0.07214100

H 1.63196500 4.75862200 -1.79502900

H 0.08424800 6.79615700 1.67198500

H 1.78367300 6.61055000 -0.13933100

C 4.54931900 -0.90394100 3.27439600

C 5.32930300 -1.42893100 2.28274000

C 3.19449200 -1.72794900 1.70272900

N 3.22092900 -1.10310800 2.89752500

H 4.81365200 -0.41450100 4.19494300

H 6.39861700 -1.48266000 2.18205100

H 2.31024700 -1.99134300 1.14247000

N 4.46710100 -1.94173400 1.31259400

C 2.04084600 -0.59392100 3.61819000

H 2.11707300 -0.86518000 4.67283400

H 1.98985200 0.49091300 3.50206900

H 1.14407400 -1.03133400 3.17901700

C 4.87935900 -2.51168200 0.00517700

H 5.64829900 -3.26605400 0.20322600

H 4.00004700 -3.00243700 -0.41614800

C 5.38247100 -1.42649900 -0.95857800

H 4.60068900 -0.66495900 -1.06458900

H 6.27017000 -0.93313500 -0.53572000

C 5.72116500 -2.01740600 -2.33984800

H 6.46688400 -2.81961000 -2.22901200

H 4.81606400 -2.47782400 -2.75728900

C 6.24992000 -0.95357300 -3.31456300

H 7.16653100 -0.48512900 -2.93395700

H 6.47860400 -1.39408200 -4.29116100

H 5.50616300 -0.16335800 -3.46914300

Fe 0.36928100 -0.29271300 -0.61989900

Cl 1.68461900 -2.04171500 -1.48949300

Cl -0.40139700 -1.67556500 1.13995200

Cl 2.13895900 0.99069300 0.12116700

Cl -0.64162600 0.19699400 -2.61271400

**b-ts2**

SCF energy in solvent model (SMD): -3192.31757900 a.u.

C -6.55536600 -2.45378100 0.19695800

C -5.56037300 -1.50595400 0.38542700

C -4.19635000 -1.82461400 0.17182000

C -3.87714900 -3.13826600 -0.24021800

C -4.86918600 -4.09589500 -0.43307300

C -6.20993300 -3.75205700 -0.21372200

H -7.60094000 -2.22025800 0.35655900

H -5.83968300 -0.50644200 0.69978600

H -2.83731700 -3.39947900 -0.40957900

H -4.60390400 -5.09990400 -0.75208000

O -7.25221700 -4.64619800 -0.38251300

H -6.94158100 -5.52440500 -0.67646900

C -3.11221400 -0.87822400 0.34889600

H -2.11787900 -1.27136300 0.15891100

C -3.20300100 0.42475200 0.73470000

H -4.14880500 0.90004300 0.96486700

C -2.03451700 1.25826000 0.86687900

O -0.84417700 0.94584200 0.65418700

O -2.29732900 2.54446400 1.36147200

C -2.69147900 3.59210700 0.49309400

C -2.53578200 3.51293000 -0.89382300

C -3.22917700 4.72429700 1.10935400

C -2.94500600 4.60145900 -1.67420100

H -2.08865600 2.64111400 -1.35957500

C -3.62654200 5.80287900 0.31475200

H -3.32366000 4.74431900 2.18852200

C -3.48985300 5.74458000 -1.07884400

H -2.82594900 4.55100500 -2.75154300

H -4.04348100 6.68758800 0.78523900

H -3.80012200 6.58359100 -1.69263600

C 5.18234000 0.08012600 3.05337600

C 5.98381000 -0.27483900 2.00475400

C 3.92736600 -1.03062400 1.57950600

N 3.90435500 -0.40345900 2.77287700

H 5.40045300 0.62536700 3.95445100

H 7.02968900 -0.09881800 1.82701000

H 3.08182600 -1.48245200 1.08418500

N 5.18448600 -0.97053400 1.09680200

C 2.69437800 -0.14364900 3.57359300

H 2.91303800 -0.33085300 4.62636500

H 2.37902700 0.89087300 3.42079600

H 1.89600000 -0.80158200 3.23017500

C 5.61256200 -1.45500100 -0.23939400

H 6.53635300 -2.02706500 -0.10193800

H 4.82948700 -2.12693100 -0.59621000

C 5.80191900 -0.30042800 -1.23447700

H 4.86726600 0.26992300 -1.28762900

H 6.58620900 0.38077000 -0.87282200

C 6.16997200 -0.82338000 -2.63558100

H 7.08365200 -1.43454800 -2.57659300

H 5.36634300 -1.48348800 -2.98787900

C 6.37451000 0.31553400 -3.64671400

H 7.18533900 0.98565000 -3.33380400

H 6.62816900 -0.07863700 -4.63688000

H 5.46206000 0.91429500 -3.74752100

Fe 0.71750400 -0.17013000 -0.43774900

Cl 2.28809500 -1.51621900 -1.54820500

Cl 0.40104700 -1.79122700 1.24784500

Cl 2.20510800 1.45955400 0.22056100

Cl -0.65105600 0.12547300 -2.24629200

**b-4**

SCF energy in solvent model (SMD): -3192.32414419 a.u.

C -6.58397600 -2.30352600 -0.26110800

C -5.54756600 -1.43212100 0.04196800

C -4.19460200 -1.83176800 -0.08477300

C -3.92930300 -3.14624300 -0.52943200

C -4.96301000 -4.02734600 -0.83803000

C -6.29187000 -3.60419600 -0.70318800

H -7.62169800 -2.00715600 -0.16812500

H -5.78624400 -0.42972000 0.38028200

H -2.89782300 -3.46726200 -0.63679400

H -4.73838400 -5.03291800 -1.18266500

O -7.37364900 -4.41990000 -0.98831600

H -7.09707000 -5.30305200 -1.30072200

C -3.06677400 -0.96592800 0.20973500

H -2.08326500 -1.41940600 0.09490600

C -3.09972000 0.33555100 0.60265200

H -4.02567200 0.88108100 0.73130200

C -1.85713100 1.04054500 0.84807000

O -0.72119400 0.51183900 0.82879500

O -1.90240400 2.38146700 1.17886000

C -3.02324400 3.20831200 0.89603900

C -3.34520700 3.50271600 -0.43112700

C -3.70964800 3.77525600 1.96945500

C -4.40549600 4.38073900 -0.68138800

H -2.76316400 3.06655600 -1.23576800

C -4.76479300 4.65568600 1.70295500

H -3.41025300 3.53525000 2.98293100

C -5.11606400 4.95582200 0.38065400

H -4.66747700 4.62175400 -1.70622100

H -5.30740600 5.10687500 2.52713900

H -5.93348700 5.64009000 0.17875100

C 5.20099900 -1.67285200 2.86277300

C 6.08633200 -1.30734900 1.88805900

C 4.11011400 -1.66866600 0.91508000

N 3.97453600 -1.89841000 2.23683000

H 5.32900900 -1.78819000 3.92443400

H 7.12841300 -1.04773500 1.94427100

H 3.33035500 -1.73039600 0.17154000

N 5.38908900 -1.31457700 0.67920000

C 2.70540300 -2.19972200 2.92168400

H 2.85891800 -3.02444900 3.62029300

H 2.36125200 -1.30684900 3.44813700

H 1.95754500 -2.47026700 2.17570900

C 5.92669700 -0.87381100 -0.63253400

H 6.88732700 -1.37873200 -0.77998700

H 5.22367200 -1.21857400 -1.39322800

C 6.06919600 0.65381800 -0.70752100

H 5.09418600 1.10530900 -0.48965900

H 6.77435800 1.00117900 0.06204900

C 6.55043100 1.10409300 -2.09936800

H 7.50598600 0.61322200 -2.33994300

H 5.82169200 0.76955900 -2.84945300

C 6.71587900 2.62910000 -2.19259900

H 7.45304200 2.99504600 -1.46650800

H 7.05170400 2.92800800 -3.19164600

H 5.76515000 3.13630800 -1.99235400

Fe 0.93386900 0.12138900 -0.52961400

Cl 2.68028000 -0.39806200 -2.02664200

Cl 0.55872800 -2.15535800 -0.03991200

Cl 2.32504300 1.09705400 1.04440200

Cl -0.32043500 1.25185800 -2.08219800

**b-5**

SCF energy in solvent model (SMD): -3308.04565324 a.u.

C -4.90812300 2.88094600 -0.15728500

C -4.13804000 1.74110400 -0.29433800

C -2.81831000 1.79974300 -0.78705800

C -2.30542500 3.05473300 -1.14612600

C -3.07019300 4.20661300 -1.01104000

C -4.37535900 4.12177800 -0.51903200

H -5.92688200 2.83521900 0.20703300

H -4.58501300 0.78514500 -0.05076200

H -1.28720200 3.12499700 -1.51244300

H -2.65157900 5.17177000 -1.28029600

O -5.18980500 5.20808100 -0.38182400

H -4.71553500 6.00905200 -0.63043500

C -2.01438700 0.60003000 -0.97273500

H -1.15758800 0.66668700 -1.63533300

C -2.31164600 -0.58075700 -0.40204400

H -3.13452300 -0.63666400 0.29326200

C -1.67523000 -1.84207200 -0.74903300

O -0.61522900 -2.03589900 -1.28459500

O -2.45064100 -2.96716200 -0.40427000

C -3.82903300 -2.78743700 -0.27972200

C -4.42354500 -2.88437200 0.97428200

C -4.58394500 -2.47035000 -1.40691900

C -5.78673900 -2.62045700 1.10533700

H -3.81779800 -3.13147300 1.83821400

C -5.94326500 -2.20529500 -1.26664600

H -4.09398700 -2.40097500 -2.37057900

C -6.54647700 -2.26950300 -0.01000600

H -6.25079500 -2.68640700 2.08263000

H -6.53071100 -1.94361900 -2.13897700

H -7.60452200 -2.06068600 0.09842600

C 3.98086400 -3.81313200 -0.23982300

C 4.82707500 -2.86396600 -0.72168000

C 2.75663200 -2.15072200 -1.02530100

N 2.69530000 -3.35475000 -0.44806300

H 4.17561900 -4.76197800 0.22803600

H 5.90107800 -2.82831500 -0.75738800

H 1.90565700 -1.53148000 -1.26830300

N 4.04443000 -1.83702100 -1.21384200

C 1.46717600 -3.99620900 0.04087000

H 1.64718500 -5.06589100 0.11662500

H 1.19886000 -3.57316300 1.00828300

H 0.65839000 -3.79276900 -0.65512000

C 4.56124700 -0.56088600 -1.74693200

H 5.20143700 -0.79693200 -2.60091000

H 3.70158400 0.00271800 -2.10478900

C 5.31894200 0.23497700 -0.68363200

H 4.68234400 0.35256500 0.19737400

H 6.20611200 -0.32679600 -0.36684900

C 5.73498200 1.61229300 -1.21054100

H 6.24312100 1.50083700 -2.17742100

H 4.83147200 2.20323300 -1.39039000

C 6.64778400 2.35453600 -0.23170300

H 7.57951000 1.80328700 -0.06548800

H 6.90759200 3.34578600 -0.61175400

H 6.15097000 2.48581100 0.73182900

Fe 1.69988400 1.37374800 0.83856500

Cl 3.32722800 2.81530200 1.26426900

Cl 1.50358300 1.19456700 -1.40737300

Cl 2.27487400 -0.63061100 1.66401000

Cl -0.22579000 2.02601700 1.69909500

C -1.09203400 -1.56614200 2.81629200

H -0.26195100 -0.86736800 2.91859500

H -1.33906100 -1.98472800 3.81143200

H -1.95589100 -1.01395900 2.44471900

O -0.67083200 -2.58409500 1.90454400

H -1.43688800 -3.08875300 1.60944300

**b-ts3**

SCF energy in solvent model (SMD): -3307.97594997 a.u.

C -5.28276600 2.80012900 -0.30683300

C -4.40906600 1.72079400 -0.30699100

C -3.04515700 1.89126100 -0.64305600

C -2.59459400 3.18124400 -0.99418000

C -3.46300400 4.27028000 -0.98664800

C -4.80742800 4.07814300 -0.64006600

H -6.33130000 2.68389000 -0.06160600

H -4.78910000 0.73167000 -0.07243900

H -1.54878700 3.32706600 -1.24409600

H -3.09833800 5.26081700 -1.24320000

O -5.73295400 5.10944300 -0.61520100

H -5.33198100 5.96984300 -0.84463000

C -2.08974700 0.79620800 -0.63367000

H -1.15198700 0.97077100 -1.15683000

C -2.25138700 -0.39012400 0.00297100

H -3.12020100 -0.61902800 0.60355800

C -1.22700200 -1.39264900 -0.09048800

O -0.27581400 -1.59463000 -0.80372400

O -2.26731100 -3.13560600 0.15775900

C -3.62475300 -3.09475700 -0.02930000

C -4.50627300 -3.03571100 1.06829300

C -4.14878600 -3.05496200 -1.33550900

C -5.88686600 -2.94749700 0.85901700

H -4.09799100 -3.07192200 2.07450600

C -5.52745900 -2.96107600 -1.53524300

H -3.45635800 -3.09146500 -2.16906600

C -6.40487900 -2.90512400 -0.44163400

H -6.55725300 -2.90828100 1.71255300

H -5.92115900 -2.92749600 -2.54672700

H -7.47543600 -2.83182900 -0.60219600

C 3.70989000 -4.04109200 -0.18184300

C 4.61229500 -3.17697000 -0.73637400

C 2.58744100 -2.24193400 -0.88121800

N 2.45314000 -3.44475400 -0.28467400

H 3.84384900 -5.00816000 0.26887000

H 5.67814800 -3.24894800 -0.85707900

H 1.78795900 -1.54410000 -1.06494300

N 3.89249500 -2.06440900 -1.17127200

C 1.19919700 -3.99423600 0.26947900

H 1.39124000 -5.01904600 0.58821600

H 0.87635100 -3.37844100 1.10973100

H 0.41146900 -3.97432800 -0.48244800

C 4.48687500 -0.83985000 -1.75715800

H 5.08168000 -1.14300900 -2.62549600

H 3.66017900 -0.21711600 -2.10271300

C 5.34258700 -0.07345500 -0.73898100

H 4.73624900 0.15062600 0.14626700

H 6.17875100 -0.70684400 -0.40791100

C 5.88566400 1.23555500 -1.33972600

H 6.38688900 1.02412400 -2.29690700

H 5.04007000 1.90073800 -1.55756700

C 6.86137000 1.94367100 -0.38798000

H 7.73165700 1.31050200 -0.17132800

H 7.22474600 2.88067200 -0.82377800

H 6.36527600 2.18461900 0.55778600

Fe 1.85977500 1.60084800 0.72704900

Cl 3.60057200 2.97537000 0.98743200

Cl 1.54478000 1.25167600 -1.50102000

Cl 2.31573300 -0.38861900 1.71105400

Cl -0.02896500 2.43691100 1.59596500

C -1.11236200 -1.11209900 2.75101400

H -0.57875600 -0.18250400 2.55651500

H -0.69002800 -1.60233600 3.63101400

H -2.17815600 -0.91109900 2.90003100

O -0.89664000 -1.99579200 1.60426300

H -1.62832100 -2.74986000 1.33390400

**MPC+cat+phenol**

SCF energy in solvent model (SMD): -3308.06488087 a.u.

C -2.92444200 3.87091700 0.27652700

C -2.40061500 2.59134000 0.32163300

C -2.62350200 1.67710100 -0.72656600

C -3.37325600 2.11029000 -1.83110100

C -3.90942600 3.39058000 -1.88387100

C -3.68769900 4.27412500 -0.82450500

H -2.74210000 4.57930100 1.07513400

H -1.78560000 2.30772500 1.16618200

H -3.54910800 1.42579000 -2.65394400

H -4.49342000 3.70597800 -2.74335600

O -4.18053500 5.54675000 -0.80964700

H -4.67111100 5.72114100 -1.61979900

C -2.10169200 0.32283800 -0.71300000

H -2.11762600 -0.21118900 -1.65845300

C -1.62783700 -0.34685400 0.35495100

H -1.62411900 0.09400000 1.33955400

C -1.12247200 -1.70345700 0.17340700

O -0.79652000 -2.18393600 -0.90060900

O -3.12907100 -4.26193100 -0.06054600

C -3.91255800 -3.13897400 -0.17923000

C -4.38241700 -2.44999500 0.94119400

C -4.24731000 -2.69771800 -1.45870800

C -5.15572100 -1.30230000 0.77863600

H -4.14107300 -2.82016000 1.93139500

C -5.03056500 -1.55655000 -1.61182100

H -3.86272800 -3.24161000 -2.31251400

C -5.48000800 -0.84725800 -0.49740000

H -5.50303300 -0.76262200 1.65255900

H -5.27948600 -1.21137600 -2.60961100

H -6.06914200 0.05322500 -0.62258900

C 3.84279200 -3.52425800 -0.96567400

C 4.37953800 -2.44406900 -1.59250800

C 2.21277200 -2.08409700 -1.34884000

N 2.48884200 -3.28717700 -0.83369200

H 4.29944600 -4.42790800 -0.60186100

H 5.39194400 -2.22902800 -1.88374000

H 1.23788100 -1.62068300 -1.35117800

N 3.34404600 -1.55966000 -1.82993700

C 1.54910400 -4.13967500 -0.09611000

H 1.77129200 -5.18118600 -0.32614200

H 1.66396900 -3.94563300 0.97035800

H 0.53524500 -3.88471000 -0.39391900

C 3.48025100 -0.23214000 -2.46159200

H 3.82762400 -0.38817600 -3.48645500

H 2.48153800 0.20047900 -2.49462100

C 4.42648100 0.67858800 -1.68222300

H 4.09885500 0.72878300 -0.64148700

H 5.43712800 0.25288400 -1.68136700

C 4.45947400 2.08874400 -2.27987800

H 4.72606100 2.03282600 -3.34337700

H 3.45210700 2.51294700 -2.22486400

C 5.44133500 3.00477700 -1.54471800

H 6.46364600 2.61661400 -1.60205300

H 5.44005200 4.00836900 -1.97776600

H 5.17125500 3.09017100 -0.48988100

Fe 1.79680500 1.03904100 1.49454500

Cl 3.68497200 2.09578900 1.94049100

Cl 1.14637800 1.51609500 -0.59015600

Cl 2.16489400 -1.17835500 1.62399900

Cl 0.18160500 1.54736000 2.94262300

C -1.21171300 -2.02156300 2.59880800

H -0.45523200 -1.27071600 2.82453700

H -1.07599500 -2.88314200 3.25014000

H -2.21426900 -1.61201000 2.73238800

O -1.03715600 -2.53033800 1.25760900

H -2.44139400 -4.08913700 0.59676700

**c-1**

SCF energy in gas phase: -2503.81028091 a.u.

Fe -1.80623200 0.24509800 -0.31825600

Cl -3.00809600 2.11132800 -0.58912900

Cl -0.37642800 0.23349300 1.47714400

Cl -2.15037600 -1.98689000 -0.91501700

Cl -0.26914100 0.63487500 -2.01996600

C -3.76561300 0.29461200 2.33766700

H -4.66987300 -0.13247100 2.78396500

H -3.93780300 1.34443200 2.10184500

H -2.92026100 0.20968300 3.02764600

O -3.48333000 -0.35334700 1.08939000

H -3.35274400 -1.30565600 1.20746200

C 3.18631300 -2.77418400 0.74943300

C 3.82647700 -1.60672600 0.46082400

C 1.80961800 -1.51623600 -0.44004800

N 1.92855300 -2.69833600 0.17654800

H 3.51070900 -3.64040700 1.30317800

H 4.81460800 -1.26204300 0.71902300

H 0.94129600 -1.14569100 -0.97544900

N 2.95276200 -0.83782300 -0.28712100

C 0.88187200 -3.72324700 0.24719100

H -0.03941700 -3.31279500 -0.17065800

H 1.19186800 -4.60564800 -0.31724300

H 0.71482200 -3.98992800 1.29240400

C 3.18887600 0.54211100 -0.76415800

H 4.15515400 0.54469500 -1.27869600

H 2.40372900 0.75438400 -1.49270400

C 3.15081400 1.56436700 0.37516800

H 2.18401000 1.48224000 0.88373500

H 3.93018800 1.32852500 1.11298200

C 3.34669500 2.99332700 -0.14973300

H 4.30148600 3.06192300 -0.68967900

H 2.55861500 3.21526200 -0.87974300

C 3.31335200 4.03681300 0.97119700

H 4.10699600 3.85875800 1.70597800

H 3.44907200 5.04651900 0.57205600

H 2.35528300 4.01282000 1.50066100

**c-ts1**

SCF energy in gas phase: -2503.80229245 a.u.

Fe 1.76031200 0.53238000 -0.05961000

Cl 2.89596600 2.16789100 -1.06159900

Cl 0.68988600 -0.50490900 -1.72933500

Cl 1.29315100 -2.01054200 1.60403100

Cl 0.38331800 1.54643700 1.36823700

C 4.60966400 -0.74312700 -0.08288700

H 5.37452600 -1.09526100 0.61420300

H 4.94805700 0.16394900 -0.58182800

H 4.38944800 -1.51678600 -0.82476300

O 3.42627200 -0.40027200 0.66105100

H 2.99942600 -1.18693900 1.11524800

C -3.20123900 -2.68026500 -0.94236000

C -3.79421800 -1.52838700 -0.52189200

C -1.76693300 -1.61296300 0.36150500

N -1.93935800 -2.71564700 -0.37781400

H -3.56268300 -3.46763000 -1.58397100

H -4.77144200 -1.12247400 -0.72672200

H -0.85811200 -1.38645400 0.91624600

N -2.88440100 -0.87813900 0.29445000

C -0.91759100 -3.74613600 -0.60089000

H -0.05943300 -3.51824300 0.03503100

H -1.33307000 -4.72381800 -0.34823900

H -0.61055000 -3.72368900 -1.64826900

C -3.07706400 0.44401500 0.92262900

H -4.01761100 0.40596600 1.48175500

H -2.25759200 0.57670800 1.63124500

C -3.08060400 1.57861600 -0.10571700

H -2.13004200 1.55930100 -0.64992400

H -3.88011100 1.41088500 -0.84019300

C -3.26891000 2.94647600 0.56369800

H -4.20908600 2.95152200 1.13275000

H -2.46086700 3.10078900 1.28865500

C -3.27246100 4.09640600 -0.44816600

H -4.08187300 3.98385500 -1.17868600

H -3.40737200 5.05960800 0.05303400

H -2.32689500 4.13677000 -0.99876600

**c-2**

SCF energy in gas phase: -2503.80506698 a.u.

Fe -1.82919600 -0.88325100 -0.28402000

Cl -3.41902800 -2.22434200 -0.98996800

Cl -0.97952700 0.35942100 -1.92828700

Cl -1.02805600 2.32237100 2.18270800

Cl -0.20903700 -1.89636000 0.84022700

C -4.02125800 0.87570800 0.88337000

H -4.28708400 1.31887900 1.84546500

H -4.69449500 0.05155700 0.64720400

H -4.06843500 1.63633700 0.09859900

O -2.68644900 0.34279200 0.98767600

H -2.05761100 1.04603200 1.45515400

C 2.63277000 2.63131900 -1.49423000

C 3.37283200 1.58405900 -1.03398900

C 1.58305800 1.77422700 0.25393700

N 1.52490400 2.73601400 -0.67476700

H 2.79505200 3.29855600 -2.32531600

H 4.30433600 1.17346500 -1.38659900

H 0.83360800 1.64557900 1.03882800

N 2.70034100 1.06198500 0.05813700

C 0.41580800 3.68669300 -0.82574600

H -0.22493300 3.60174000 0.05529200

H 0.82100300 4.69709400 -0.90739500

H -0.15446300 3.43022500 -1.72066700

C 3.12354400 -0.09101600 0.87810500

H 4.02746300 0.20055500 1.42377700

H 2.32539200 -0.26100600 1.60353800

C 3.36216600 -1.35133300 0.04357200

H 2.44929500 -1.58120700 -0.51587300

H 4.16047400 -1.17080500 -0.68871900

C 3.74339000 -2.54559800 0.92925200

H 4.63639000 -2.29650300 1.51894500

H 2.93485500 -2.72808100 1.64753700

C 4.00096300 -3.81811600 0.11670700

H 4.82162300 -3.67760000 -0.59607000

H 4.26740600 -4.65390200 0.77053800

H 3.11044300 -4.10889200 -0.45016400

**c-3**

SCF energy in gas phase: -3307.97225308 a.u.

Fe -0.60789900 -2.43925400 -1.17617300

Cl -2.61872900 -2.22394400 -2.02813900

Cl 0.03656700 -4.55571300 -1.03121500

Cl 3.34940400 -1.23570700 -1.68170200

Cl -0.28188700 -1.38236600 0.75238600

C 0.58310500 -1.77795600 -3.87662800

H -0.44159700 -1.62625600 -4.21650500

H 0.91425800 -2.79367000 -4.10963000

H 1.24978000 -1.05133200 -4.34522200

O 0.61076700 -1.55694400 -2.44966700

H 1.59794000 -1.42890100 -2.13634200

C 3.24890700 -2.40597300 3.69624100

C 4.03941800 -1.31356400 3.50237900

C 3.27995000 -1.96333100 1.52744700

N 2.78774400 -2.79825900 2.45193000

H 2.97816800 -2.93174600 4.59772600

H 4.59196700 -0.71105400 4.20474000

H 3.11476200 -1.98505300 0.44695300

N 4.04771800 -1.05499400 2.14299400

C 1.87579700 -3.91700000 2.19028400

H 2.27298500 -4.81976600 2.65836100

H 0.88706600 -3.68393300 2.58856100

H 1.78950400 -4.06675400 1.11507200

C 4.77604200 0.04006100 1.46454400

H 5.83227100 -0.06203700 1.73477900

H 4.67136400 -0.14039600 0.39132700

C 4.24148400 1.42287300 1.84680500

H 3.19944500 1.50720600 1.51741600

H 4.24388700 1.53486100 2.93982700

C 5.08146600 2.54311300 1.21666000

H 6.13256300 2.41808100 1.51204200

H 5.04927300 2.45522800 0.12459800

C 4.59793400 3.93791300 1.62554700

H 4.63774300 4.07332500 2.71256200

H 5.22112100 4.71527900 1.17232100

H 3.56553900 4.10769000 1.30352100

C 2.10840200 2.38635200 -1.44625000

C 0.78821000 2.27968500 -1.03443300

C -0.02134500 3.42109500 -0.84318400

C 0.55534300 4.67894300 -1.09145400

C 1.88105100 4.80368600 -1.49843500

C 2.66146900 3.65606700 -1.67456700

H 2.71327800 1.49789800 -1.60693000

H 0.37723800 1.28972200 -0.86538800

H -0.04672200 5.57408600 -0.95937100

H 2.30702300 5.78818900 -1.68143300

O 3.97070500 3.70937100 -2.06031900

H 4.22141000 4.63075900 -2.20861600

C -1.41296300 3.35385000 -0.41153200

H -1.94692000 4.30244000 -0.36676600

C -2.11460200 2.25708300 -0.06197600

H -1.69407200 1.25766400 -0.05362100

C -3.52734000 2.38558900 0.32979000

O -4.16808400 3.41583300 0.39288500

O -4.02775600 1.13960200 0.61548100

C -5.35296500 0.91379300 0.97021600

C -6.12382900 1.79249400 1.73586300

C -5.86071300 -0.32269600 0.56408500

C -7.42274900 1.41420200 2.08340500

H -5.72306300 2.74986500 2.03669900

C -7.15800100 -0.68410900 0.92211200

H -5.22841600 -0.97974200 -0.02452700

C -7.94553900 0.18403700 1.68217400

H -8.02850000 2.09459200 2.67546700

H -7.55192400 -1.64540200 0.60493000

H -8.95753400 -0.09622000 1.95927600

**c-ts2**

SCF energy in gas phase: -3307.93282732 a.u.

Fe -2.72418600 0.14020400 0.36949900

Cl -3.97938900 -1.02828100 1.84508100

Cl -4.30356400 1.42028700 -0.58123000

Cl 1.20243200 1.16777600 2.20237200

Cl -0.91240600 1.22795600 -1.15683400

C -2.17063800 1.29262900 3.22180600

H -2.17641900 0.31500400 3.70652100

H -3.18331600 1.69487100 3.20679300

H -1.49227300 1.96995800 3.74652500

O -1.71993000 1.18115900 1.85276800

H -0.70893500 1.08975200 1.87074000

C -0.07080600 5.46276400 -1.07494200

C 1.27503500 5.25453300 -1.12577800

C 0.41460900 3.81171500 0.31286700

N -0.58793500 4.55636300 -0.16618600

H -0.69735900 6.16708400 -1.59799200

H 2.04408900 5.74794300 -1.69762500

H 0.35834700 2.99031800 1.02460700

N 1.55798500 4.22398500 -0.24611200

C -2.00853400 4.34801800 0.13630600

H -2.46946600 5.30672900 0.38143800

H -2.50465100 3.88436700 -0.71792900

H -2.08555300 3.66563300 0.98088900

C 2.87017200 3.58706200 -0.01585100

H 3.60148600 4.38867800 0.12860400

H 2.77636500 3.01874500 0.91339000

C 3.26903400 2.66017000 -1.16770900

H 2.48361700 1.90627400 -1.29346700

H 3.32095300 3.23042000 -2.10517100

C 4.61563500 1.97550000 -0.89793300

H 5.38971900 2.73812000 -0.73441000

H 4.54360900 1.39978800 0.03294700

C 5.04263500 1.04802200 -2.03971500

H 5.13961800 1.59535600 -2.98429200

H 6.00764300 0.57883100 -1.82497700

H 4.31037000 0.24791900 -2.18971900

C 4.43923900 -2.00840400 1.67112400

C 3.29228800 -1.68821300 0.96430400

C 3.01483700 -2.30457600 -0.27907900

C 3.93578000 -3.24729200 -0.77571600

C 5.08666500 -3.57495700 -0.06979000

C 5.34243500 -2.95509000 1.16023700

H 4.65548000 -1.53955200 2.62509000

H 2.61236400 -0.94759500 1.37861200

H 3.74088700 -3.73229500 -1.72867700

H 5.78444500 -4.30817800 -0.46806000

O 6.44754700 -3.22553800 1.90288600

H 6.98068000 -3.89740800 1.45729200

C 1.82923800 -2.01058900 -1.06424800

H 1.76156700 -2.52240400 -2.02298300

C 0.81539500 -1.17504500 -0.72002200

H 0.76825200 -0.61116000 0.20264400

C -0.25776200 -1.02905300 -1.66005900

O -0.58291100 -1.31323900 -2.74254700

O -1.86074400 -1.39350900 -0.42444900

C -2.32396600 -2.66849800 -0.58706200

C -3.24295600 -2.95729800 -1.60487500

C -1.83143700 -3.69499700 0.23050400

C -3.67392400 -4.27034200 -1.79159800

H -3.61060400 -2.14866600 -2.22815800

C -2.26821600 -5.00344800 0.03190700

H -1.12609300 -3.44828900 1.01723800

C -3.18893600 -5.29691700 -0.97793100

H -4.39202000 -4.49068300 -2.57651700

H -1.89216000 -5.79640300 0.67256700

H -3.52904900 -6.31763000 -1.12615800

**c-4**

SCF energy in gas phase: -3307.95313871 a.u.

Fe 0.00041900 3.16248400 -0.91741300

Cl 1.03492800 4.98773400 -1.62252200

Cl -2.22583200 3.39212300 -0.73083300

Cl -0.62217200 -0.90628300 -1.73514300

Cl -1.58392500 -2.46179100 2.52857800

C -0.06377400 2.04468000 -3.73246400

H 0.43440900 2.96841700 -4.02859000

H -1.14642100 2.14833500 -3.85092100

H 0.29923400 1.21173300 -4.33873100

O 0.27386800 1.79107000 -2.35479700

H 0.00200300 0.83409100 -2.10605800

C -4.42391600 0.77168400 1.95799600

C -5.02408600 0.07842100 0.95010800

C -2.84913000 0.11787200 0.55080300

N -3.06766500 0.78411800 1.68962100

H -4.83852500 1.25942300 2.82522100

H -6.06186400 -0.15231800 0.77255500

H -1.89585400 -0.06316500 0.05802700

N -4.02366100 -0.31877900 0.08124200

C -2.02665600 1.39391700 2.52168700

H -2.40101000 2.33964500 2.91526900

H -1.76851300 0.71608200 3.33834100

H -1.15073800 1.59107100 1.90360300

C -4.18832200 -1.13532700 -1.13759900

H -5.04411700 -0.72819100 -1.68498600

H -3.28387300 -0.98621400 -1.73344300

C -4.38017100 -2.62027500 -0.81635600

H -3.52175200 -2.96447000 -0.22770500

H -5.27250700 -2.75579700 -0.19003600

C -4.50608400 -3.45986100 -2.09457100

H -5.34954500 -3.09101300 -2.69445600

H -3.60545700 -3.31622800 -2.70411400

C -4.69841600 -4.95122200 -1.80263300

H -5.60815800 -5.12925600 -1.21797000

H -4.78041800 -5.52687300 -2.72970600

H -3.85352100 -5.35372500 -1.23371000

C 3.53780000 -2.44190100 -2.22192900

C 2.65361100 -2.52705700 -1.16075900

C 3.04640700 -3.12295400 0.06143700

C 4.35953900 -3.62447200 0.16563600

C 5.25172300 -3.54232700 -0.89500400

C 4.84195100 -2.94823500 -2.09640500

H 3.24144800 -1.98519100 -3.16002300

H 1.65165100 -2.12365600 -1.28236400

H 4.68042200 -4.08546300 1.09613000

H 6.26132900 -3.93482200 -0.79600000

O 5.66062400 -2.83422900 -3.17399300

H 6.52648000 -3.20879600 -2.96369200

C 2.16898000 -3.24235100 1.20666100

H 2.60423200 -3.71935600 2.08413900

C 0.87486600 -2.83782400 1.31820800

H 0.32584800 -2.35512200 0.51728100

C 0.19980700 -3.07678700 2.57947000

O 0.58259200 -3.57721700 3.59325300

O 0.66121200 2.34841800 0.57551900

C 1.69916200 2.36689800 1.43434900

C 2.03873200 1.17986200 2.10887800

C 2.42494000 3.54542900 1.68616300

C 3.09038200 1.17831900 3.02175000

H 1.48122800 0.27444800 1.89084700

C 3.47222700 3.52702500 2.60415300

H 2.15873500 4.45382400 1.15518700

C 3.81043000 2.34906200 3.27713500

H 3.34954500 0.25664200 3.53529000

H 4.02857100 4.44060700 2.79440500

H 4.62817500 2.34330400 3.99141300

**c-5**

SCF energy in gas phase: -3307.96169153 a.u.

Fe -0.80950700 -2.23892500 -0.71465700

Cl -0.18154900 -2.19230100 1.49746100

Cl -0.22577000 -0.24734800 -1.60545800

Cl 0.43517200 -3.84838400 -1.73063000

Cl 2.33502200 1.21592400 3.75996300

C -2.35206300 0.17268000 3.68979700

H -3.07764100 -0.63342300 3.51775100

H -2.89002700 1.04799000 4.06369100

H -1.64217200 -0.15187000 4.46212400

O -1.69527700 0.56120000 2.48936900

H -1.19062400 -0.20387300 2.16184400

C -2.87524400 3.65964600 -0.15721100

C -1.71635900 3.20337800 0.44564900

C -0.46173600 3.77571500 0.12541300

C -0.42670900 4.81196600 -0.82898500

C -1.58629400 5.27931300 -1.43241100

C -2.81873300 4.70259800 -1.09650000

H -3.83816900 3.21505100 0.06980600

H -1.77648800 2.37973500 1.15116800

H 0.52803200 5.25862400 -1.09437800

H -1.54065000 6.08307400 -2.16383800

O -3.99144200 5.10669200 -1.64835800

H -3.82228400 5.80748700 -2.29214400

C 0.78279500 3.33734600 0.71949400

H 1.68629700 3.77338500 0.29418200

C 0.95283400 2.47090900 1.75459700

H 0.12849900 2.00013800 2.27857100

C 2.30055500 2.14895800 2.17161100

O 3.35396400 2.39267000 1.63393800

O -2.58648000 -2.55653600 -0.85402900

C -3.82665600 -2.12403100 -0.59767300

C -4.06933400 -1.03712200 0.26568600

C -4.91109600 -2.78696500 -1.20402300

C -5.37968000 -0.63110100 0.50783500

H -3.23619500 -0.52621600 0.73886500

C -6.21330300 -2.36646600 -0.95100000

H -4.70262700 -3.62225100 -1.86478900

C -6.45683500 -1.28779800 -0.09502800

H -5.56097200 0.20670700 1.17602600

H -7.04377500 -2.88335200 -1.42422100

H -7.47451700 -0.96303600 0.10022100

C 4.08853800 -1.86182100 1.66952900

C 4.20700600 -0.64120300 1.07522900

C 3.00493100 -1.93582000 -0.25426500

N 3.34056000 -2.65736000 0.82149800

H 4.44485800 -2.21939200 2.62175200

H 4.65470000 0.27769600 1.41865700

H 2.38450700 -2.29104000 -1.06670400

N 3.52380600 -0.70852400 -0.12537100

C 2.89306500 -4.02645100 1.09727900

H 2.25078600 -4.01924600 1.97904600

H 2.31150800 -4.38042600 0.24637900

H 3.76325500 -4.66643100 1.25764200

C 3.39954400 0.38086600 -1.11504600

H 2.40406700 0.29279700 -1.55781900

H 3.43595000 1.31323700 -0.54684800

C 4.50096600 0.32938300 -2.17831700

H 5.48405300 0.37662900 -1.69166100

H 4.45358200 -0.63315800 -2.70413600

C 4.36412800 1.47645800 -3.18856200

H 3.37300300 1.42792900 -3.65729800

H 4.40617800 2.43514100 -2.65490600

C 5.44749000 1.44313200 -4.27096400

H 5.40654300 0.51035000 -4.84408700

H 5.32584600 2.27133000 -4.97564200

H 6.44925600 1.52187300 -3.83393400

**c-ts3**

SCF energy in gas phase: -3307.93436189 a.u.

Fe -2.07129400 1.17840600 -1.11957400

Cl -2.92641500 0.65124200 0.92611000

Cl -0.02378600 0.19365900 -1.31201100

Cl -3.41266200 0.27722200 -2.71466000

Cl 0.35913500 -2.09256900 2.79746200

C -0.62544400 1.25663000 3.89915100

H -1.66525900 1.49035700 3.66062800

H -0.10900500 2.15636900 4.23480400

H -0.57011600 0.48399200 4.67169100

O 0.02631200 0.81658100 2.69543000

H -0.41792700 -0.00834200 2.39411400

C 4.69345400 0.25904800 -1.95759100

C 3.89653900 0.19923600 -0.82760100

C 4.46868300 0.10636400 0.46203700

C 5.87279700 0.08223300 0.56292000

C 6.68086600 0.13918600 -0.56584000

C 6.09231600 0.22757500 -1.83384500

H 4.25721900 0.33571800 -2.94766100

H 2.81827700 0.23461100 -0.94750500

H 6.33495800 0.01448400 1.54421900

H 7.76374800 0.11686200 -0.46647600

O 6.81924100 0.28784400 -2.97953500

H 7.76191700 0.26796300 -2.76709200

C 3.68134300 0.04418700 1.68001800

H 4.25111700 0.02140900 2.60742700

C 2.32883100 0.01062400 1.77312000

H 1.65180100 0.00773000 0.92989800

C 1.74818200 -0.02739800 3.08252300

O 2.01212100 0.09084200 4.21496000

O -1.92548500 2.97987000 -1.29887700

C -1.35186300 4.06367500 -0.76290100

C -0.53322400 3.98157000 0.38119400

C -1.57847400 5.31611400 -1.36569300

C 0.04273300 5.13768200 0.90188300

H -0.36217500 3.01468600 0.84466600

C -0.99565500 6.46137400 -0.83155300

H -2.21153700 5.35909800 -2.24612300

C -0.18248700 6.38108900 0.30337400

H 0.67441000 5.06744900 1.78356200

H -1.17622400 7.42331900 -1.30367600

H 0.27081200 7.27731000 0.71638300

C -3.68229700 -3.12688800 1.28945200

C -2.37012600 -3.49327800 1.25611300

C -2.86201000 -2.53949700 -0.67396200

N -3.97226400 -2.53642400 0.07314000

H -4.41809600 -3.20843300 2.07267500

H -1.73000500 -3.91843000 2.01191900

H -2.77299200 -2.08442400 -1.65019100

N -1.87900300 -3.12424700 0.01843100

C -5.23617600 -1.89035100 -0.28760100

H -5.44748900 -1.10204500 0.43656200

H -5.12630900 -1.43358800 -1.27060500

H -6.03857500 -2.63141500 -0.29707300

C -0.49717000 -3.33681300 -0.45500400

H -0.31607300 -2.59803500 -1.23857500

H 0.15117900 -3.08880000 0.39053100

C -0.27058300 -4.76696800 -0.95281400

H -0.49804400 -5.47621700 -0.14612000

H -0.96801100 -4.98707800 -1.77227200

C 1.17355100 -4.97570400 -1.42835700

H 1.39831900 -4.25831900 -2.22836000

H 1.86063600 -4.74256800 -0.60509300

C 1.43199700 -6.40032800 -1.92821200

H 0.78034200 -6.64986300 -2.77315000

H 2.46766100 -6.51986400 -2.26082100

H 1.24909200 -7.13748500 -1.13835600

**c-6**

SCF energy in gas phase: -3307.97142563 a.u.

Fe 1.17427700 -1.75763200 -1.30764500

Cl 2.11810100 -2.14645200 0.72668100

Cl 0.21237300 0.31034100 -1.24552300

Cl 2.85321200 -1.64769600 -2.84161100

Cl 0.97798300 1.65375300 3.38760900

C -0.43757000 -1.85905600 3.69105600

H 0.64521500 -1.91842100 3.59311300

H -0.89664800 -2.80425000 3.39710800

H -0.73339000 -1.61507700 4.71413800

O -0.84712400 -0.81441200 2.78389800

H 0.23469100 0.64184500 2.99739900

C -3.98353400 3.05093800 -1.58018300

C -3.47562500 2.16263500 -0.64617500

C -4.27910500 1.67043300 0.40603400

C -5.61749200 2.09839000 0.46604700

C -6.13637300 2.99392200 -0.46318100

C -5.31801900 3.47630900 -1.49082000

H -3.37106300 3.42059900 -2.39571800

H -2.44892500 1.82453600 -0.74665700

H -6.25873300 1.72486600 1.26002100

H -7.17359600 3.31505300 -0.39487800

O -5.75944400 4.35199100 -2.43568200

H -6.69144400 4.55132400 -2.27633600

C -3.78860300 0.74009600 1.41326600

H -4.53918600 0.30987300 2.07532000

C -2.51177600 0.35580400 1.62206300

H -1.67914100 0.72313800 1.03238800

C -2.20951200 -0.63558600 2.65846500

O -2.99903100 -1.26608400 3.33443700

O -0.04452600 -3.04475600 -1.70803600

C -1.15239300 -3.62023400 -1.22196600

C -1.72714900 -3.20268900 -0.00579200

C -1.75358300 -4.66437000 -1.94993400

C -2.88324800 -3.81802300 0.46673500

H -1.25387900 -2.39876700 0.54789300

C -2.90736000 -5.27321200 -1.46393000

H -1.29877600 -4.97391200 -2.88542400

C -3.47886500 -4.85585700 -0.25741700

H -3.32036400 -3.47896200 1.40205500

H -3.36573600 -6.07859000 -2.03157100

H -4.38062300 -5.33339300 0.11413400

C 5.24057900 0.44566700 1.46280100

C 4.44658800 1.54862100 1.56131900

C 4.00787400 0.56472200 -0.36558100

N 4.95673100 -0.15096700 0.24884800

H 5.96080600 0.02987200 2.14855700

H 4.34035800 2.27475400 2.35033700

H 3.55969900 0.30680900 -1.31646000

N 3.68641200 1.60743100 0.40849200

C 5.49903800 -1.42655100 -0.23111300

H 5.18027400 -2.22248700 0.44358200

H 5.08957200 -1.62738500 -1.22080000

H 6.58835300 -1.36392000 -0.27245800

C 2.69300800 2.64783200 0.07596800

H 1.97673700 2.18896800 -0.60959100

H 2.15507000 2.87018800 1.00094300

C 3.33936200 3.90004600 -0.52321200

H 4.07767200 4.30875400 0.17980300

H 3.88958800 3.62365400 -1.43202100

C 2.29234400 4.97172400 -0.85661200

H 1.55530300 4.55123000 -1.55235200

H 1.73899900 5.23401500 0.05456900

C 2.91199500 6.23410000 -1.46371700

H 3.44140700 6.00754000 -2.39575600

H 2.14354000 6.97901600 -1.69079500

H 3.63018500 6.69535400 -0.77646600

**c-7**

SCF energy in gas phase: -3307.97983645 a.u.

Fe -2.21824600 -0.69903400 -0.26175000

Cl -3.08111400 0.96450000 -1.53341600

Cl -0.79995400 0.18370900 1.24409500

Cl -4.61900900 -0.18318000 3.19395500

Cl -1.01587300 -2.04952300 -1.63116000

C 3.67524500 0.57066400 -1.40383500

C 4.92187400 0.24238800 -0.89266400

C 5.13902900 -0.97952100 -0.22372600

C 4.04416800 -1.85407300 -0.09280700

C 2.78778500 -1.53845700 -0.59902200

C 2.60098000 -0.31918500 -1.25892300

H 3.51614400 1.50970600 -1.92514800

H 5.74154800 0.94319800 -1.01701800

H 4.18381300 -2.80157600 0.42054600

H 1.95416000 -2.22425100 -0.48161000

O 1.39370200 0.05529900 -1.77834300

H 0.73711900 -0.66616900 -1.68193100

C 6.42962500 -1.37366900 0.33053700

H 6.46097500 -2.34491600 0.82264400

C 7.58759900 -0.68512300 0.30891300

H 7.69065200 0.29296400 -0.14921900

C 8.79711200 -1.25571200 0.92740700

O 8.88144600 -2.33462400 1.48598100

O -3.56922700 -1.61024200 0.60432400

C -4.45914800 -2.55898300 0.21445900

C -5.01295800 -2.55166300 -1.07639400

C -4.84092100 -3.54642600 1.13702400

C -5.93333800 -3.53400800 -1.43582100

H -4.72378700 -1.77273300 -1.77516800

C -5.76380500 -4.52009700 0.76312800

H -4.40628200 -3.53281700 2.13121000

C -6.31303300 -4.52102100 -0.52226000

H -6.35868700 -3.52513100 -2.43541500

H -6.05499500 -5.28250900 1.48006200

H -7.03176700 -5.28281700 -0.80862300

C -1.05602300 4.31523100 -2.58948700

C -1.15794300 4.76341700 -1.30697500

C -0.22925400 2.76006800 -1.25612800

N -0.46509800 3.06734600 -2.53762600

H -1.35021000 4.76923200 -3.52190000

H -1.55017300 5.68623600 -0.91219900

H 0.21267300 1.83531500 -0.91083100

N -0.63249300 3.77949100 -0.48918300

C -0.27756800 2.15558400 -3.67222200

H 0.06361900 2.73054000 -4.53442200

H 0.46756500 1.40892000 -3.39884900

H -1.22696200 1.66153500 -3.89046500

C -0.59193900 3.81304400 0.98827700

H -0.02517500 2.93556400 1.30370900

H -0.03537300 4.70980000 1.27965000

C -1.99142700 3.79349700 1.60867200

H -2.56135900 4.66777700 1.26635400

H -2.52041600 2.90276400 1.25437000

C -1.92405000 3.78865000 3.14207600

H -1.36471900 2.90414200 3.47059800

H -1.35855100 4.66550200 3.48794000

C -3.31367300 3.77970000 3.78655200

H -3.87339900 2.88504100 3.49685300

H -3.23702600 3.78616600 4.87802400

H -3.89682700 4.65857500 3.48790100

C 11.07901500 -0.87185400 1.36908800

H 10.96747800 -1.04265700 2.44329300

H 11.81065200 -0.08534600 1.18172900

H 11.39473800 -1.80762300 0.89985500

O 9.85004100 -0.40554100 0.79773100

H -4.12854600 -0.77484700 2.12941200

**c-ts4**

SCF energy in gas phase: -3307.97258908 a.u.

Fe 2.36279600 0.63996600 -0.79149100

Cl 2.99855700 -1.16711100 -1.91785900

Cl 0.94903600 0.21568100 0.87999700

Cl 4.75228900 -0.09590700 1.25507700

Cl 1.06062900 1.75862900 -2.27078800

C -3.43631100 -1.11993300 -1.27282300

C -4.67007900 -0.75550500 -0.75486100

C -4.92512300 0.56573100 -0.33434800

C -3.88139200 1.50154100 -0.45859300

C -2.63852200 1.15103400 -0.97506100

C -2.41323800 -0.16726900 -1.38523100

H -3.24970700 -2.13748400 -1.60238300

H -5.44994400 -1.50678700 -0.67845400

H -4.05057200 2.52641800 -0.13952300

H -1.84389200 1.88664000 -1.05507100

O -1.21636800 -0.58126200 -1.89935400

H -0.60667600 0.17654100 -1.99749900

C -6.20422300 1.00138600 0.21553900

H -6.26765800 2.05018200 0.50273500

C -7.31682200 0.26728200 0.41183600

H -7.38532400 -0.78768400 0.16787500

C -8.52226400 0.89290500 0.98352600

O -8.64044700 2.05772700 1.31880100

O 3.81880000 1.91879100 -0.36034000

C 3.72585200 3.24419400 0.05614000

C 3.44738600 4.22605600 -0.89490800

C 3.93998800 3.56553400 1.39868000

C 3.37670700 5.55780000 -0.48678200

H 3.28942800 3.94087000 -1.92898900

C 3.87374500 4.90384000 1.78639100

H 4.15740500 2.77699100 2.11170300

C 3.59031200 5.90125900 0.85047300

H 3.15887200 6.32879800 -1.21991200

H 4.04231400 5.16420300 2.82715100

H 3.53681100 6.94027300 1.16102600

C 1.28848600 -4.98046700 -1.48236700

C 1.59697400 -4.98519300 -0.15541800

C 0.58232300 -3.07773600 -0.60963500

N 0.64879600 -3.78403000 -1.74581300

H 1.46911400 -5.71293100 -2.25245200

H 2.09182000 -5.72683300 0.44958200

H 0.15157100 -2.09083600 -0.51447400

N 1.14562500 -3.78958800 0.37308000

C 0.23745000 -3.30247800 -3.06935900

H -0.28736200 -4.10447500 -3.59150400

H -0.42028200 -2.44338000 -2.93931600

H 1.12307500 -2.99764100 -3.63064600

C 1.29667400 -3.35564300 1.78132700

H 0.80690300 -2.38362500 1.85892200

H 0.74677300 -4.07256700 2.40042400

C 2.76197500 -3.25187500 2.21097500

H 3.25412200 -4.22672500 2.08989800

H 3.28240200 -2.53558400 1.56604500

C 2.87707800 -2.79941900 3.67388700

H 2.41587900 -1.80941600 3.77449400

H 2.30588200 -3.48242200 4.31935300

C 4.33260600 -2.72965500 4.14594000

H 4.89919700 -2.01833000 3.53821700

H 4.38833700 -2.40580600 5.18999000

H 4.82090300 -3.70880100 4.07454700

C -10.74833400 0.49735500 1.64528400

H -10.58776200 0.88819600 2.65373800

H -11.44008200 -0.34520800 1.66830000

H -11.14588700 1.30175300 1.02047200

O -9.52694200 -0.01558000 1.09795700

H 4.36417200 1.25807200 0.33419200

**c-8**

SCF energy in gas phase: -3307.99787640 a.u.

Fe 2.24435700 0.19748500 -0.89590000

Cl 3.30239000 1.61970800 0.46327400

Cl 0.45701500 1.26245100 -1.72966200

Cl 3.58564600 -0.52913900 -2.50361900

Cl 1.45995400 -1.52285000 0.33553900

C -3.57484500 0.05790200 1.52029000

C -4.85354400 -0.30857000 1.12802900

C -5.06135800 -1.27915400 0.12672900

C -3.92404700 -1.86398100 -0.46037600

C -2.63531600 -1.50586400 -0.07829500

C -2.45920100 -0.53959500 0.91706300

H -3.42017100 0.80035500 2.29714000

H -5.70520000 0.16340500 1.60789000

H -4.05610800 -2.61407700 -1.23533900

H -1.77076100 -1.96570200 -0.54819000

O -1.22096500 -0.13870900 1.33705800

H -0.51874600 -0.65969200 0.89559400

C -6.38459400 -1.69949400 -0.32238800

H -6.40486300 -2.45968400 -1.10220700

C -7.58404700 -1.26282800 0.10818000

H -7.70209000 -0.50892600 0.87944800

C -8.82177000 -1.81285500 -0.47314100

O -8.89414000 -2.66133400 -1.34361700

O 5.69495500 -1.76024100 -0.14305000

C 5.70298600 -3.11481700 0.01057200

C 6.46137700 -3.63838200 1.06583200

C 4.99712900 -3.97985700 -0.83592400

C 6.50923900 -5.01580400 1.26835500

H 7.00286400 -2.95168000 1.70877000

C 5.05399900 -5.35746700 -0.62091300

H 4.40771300 -3.57241000 -1.65318800

C 5.80764900 -5.88566500 0.42857800

H 7.10133600 -5.41260700 2.08893300

H 4.50186300 -6.01971700 -1.28242700

H 5.84848500 -6.95854700 0.59036100

C 1.49996800 3.54060900 3.24440600

C 1.23398100 4.47834300 2.29214600

C 0.24923700 2.59616600 1.68886900

N 0.86857400 2.37567400 2.85459300

H 2.08106900 3.59975200 4.15042800

H 1.53295700 5.51097100 2.21915900

H -0.31870600 1.85478700 1.14220600

N 0.44876000 3.86946000 1.33034400

C 0.98635400 1.06812800 3.51197500

H 0.94687900 1.21156800 4.59265000

H 0.15745300 0.43982300 3.18732900

H 1.93284100 0.60652000 3.22162600

C -0.02747900 4.48657900 0.07507800

H -0.64411400 3.73602100 -0.42305600

H -0.66535500 5.33506700 0.34382800

C 1.12559800 4.91611100 -0.83598900

H 1.73670800 5.67480000 -0.32927700

H 1.76963600 4.04964100 -1.01743700

C 0.60855800 5.47596700 -2.16821800

H 0.01275500 4.70433200 -2.67138000

H -0.06855000 6.32010900 -1.97709500

C 1.74491300 5.92577300 -3.09168600

H 2.41397100 5.09139600 -3.32613900

H 1.35218200 6.31445100 -4.03593900

H 2.34552700 6.71694400 -2.62889500

C -11.17428900 -1.71137200 -0.40227800

H -11.26372000 -1.51697200 -1.47450800

H -11.93640800 -1.16112200 0.15025800

H -11.28113600 -2.78643200 -0.23413800

O -9.91527400 -1.24128100 0.09656700

H 5.15236600 -1.51249900 -0.90862300

**MPC+cat+phenol**

SCF energy in gas phase: -3308.00468983 a.u.

C -2.92444200 3.87091700 0.27652700

C -2.40061500 2.59134000 0.32163300

C -2.62350200 1.67710100 -0.72656600

C -3.37325600 2.11029000 -1.83110100

C -3.90942600 3.39058000 -1.88387100

C -3.68769900 4.27412500 -0.82450500

H -2.74210000 4.57930100 1.07513400

H -1.78560000 2.30772500 1.16618200

H -3.54910800 1.42579000 -2.65394400

H -4.49342000 3.70597800 -2.74335600

O -4.18053500 5.54675000 -0.80964700

H -4.67111100 5.72114100 -1.61979900

C -2.10169200 0.32283800 -0.71300000

H -2.11762600 -0.21118900 -1.65845300

C -1.62783700 -0.34685400 0.35495100

H -1.62411900 0.09400000 1.33955400

C -1.12247200 -1.70345700 0.17340700

O -0.79652000 -2.18393600 -0.90060900

O -3.12907100 -4.26193100 -0.06054600

C -3.91255800 -3.13897400 -0.17923000

C -4.38241700 -2.44999500 0.94119400

C -4.24731000 -2.69771800 -1.45870800

C -5.15572100 -1.30230000 0.77863600

H -4.14107300 -2.82016000 1.93139500

C -5.03056500 -1.55655000 -1.61182100

H -3.86272800 -3.24161000 -2.31251400

C -5.48000800 -0.84725800 -0.49740000

H -5.50303300 -0.76262200 1.65255900

H -5.27948600 -1.21137600 -2.60961100

H -6.06914200 0.05322500 -0.62258900

C 3.84279200 -3.52425800 -0.96567400

C 4.37953800 -2.44406900 -1.59250800

C 2.21277200 -2.08409700 -1.34884000

N 2.48884200 -3.28717700 -0.83369200

H 4.29944600 -4.42790800 -0.60186100

H 5.39194400 -2.22902800 -1.88374000

H 1.23788100 -1.62068300 -1.35117800

N 3.34404600 -1.55966000 -1.82993700

C 1.54910400 -4.13967500 -0.09611000

H 1.77129200 -5.18118600 -0.32614200

H 1.66396900 -3.94563300 0.97035800

H 0.53524500 -3.88471000 -0.39391900

C 3.48025100 -0.23214000 -2.46159200

H 3.82762400 -0.38817600 -3.48645500

H 2.48153800 0.20047900 -2.49462100

C 4.42648100 0.67858800 -1.68222300

H 4.09885500 0.72878300 -0.64148700

H 5.43712800 0.25288400 -1.68136700

C 4.45947400 2.08874400 -2.27987800

H 4.72606100 2.03282600 -3.34337700

H 3.45210700 2.51294700 -2.22486400

C 5.44133500 3.00477700 -1.54471800

H 6.46364600 2.61661400 -1.60205300

H 5.44005200 4.00836900 -1.97776600

H 5.17125500 3.09017100 -0.48988100

Fe 1.79680500 1.03904100 1.49454500

Cl 3.68497200 2.09578900 1.94049100

Cl 1.14637800 1.51609500 -0.59015600

Cl 2.16489400 -1.17835500 1.62399900

Cl 0.18160500 1.54736000 2.94262300

C -1.21171300 -2.02156300 2.59880800

H -0.45523200 -1.27071600 2.82453700

H -1.07599500 -2.88314200 3.25014000

H -2.21426900 -1.61201000 2.73238800

O -1.03715600 -2.53033800 1.25760900

H -2.44139400 -4.08913700 0.59676700

**c-1**

SCF energy in solvent model (SMD): -2503.85717712 a.u.

Fe -1.80623200 0.24509800 -0.31825600

Cl -3.00809600 2.11132800 -0.58912900

Cl -0.37642800 0.23349300 1.47714400

Cl -2.15037600 -1.98689000 -0.91501700

Cl -0.26914100 0.63487500 -2.01996600

C -3.76561300 0.29461200 2.33766700

H -4.66987300 -0.13247100 2.78396500

H -3.93780300 1.34443200 2.10184500

H -2.92026100 0.20968300 3.02764600

O -3.48333000 -0.35334700 1.08939000

H -3.35274400 -1.30565600 1.20746200

C 3.18631300 -2.77418400 0.74943300

C 3.82647700 -1.60672600 0.46082400

C 1.80961800 -1.51623600 -0.44004800

N 1.92855300 -2.69833600 0.17654800

H 3.51070900 -3.64040700 1.30317800

H 4.81460800 -1.26204300 0.71902300

H 0.94129600 -1.14569100 -0.97544900

N 2.95276200 -0.83782300 -0.28712100

C 0.88187200 -3.72324700 0.24719100

H -0.03941700 -3.31279500 -0.17065800

H 1.19186800 -4.60564800 -0.31724300

H 0.71482200 -3.98992800 1.29240400

C 3.18887600 0.54211100 -0.76415800

H 4.15515400 0.54469500 -1.27869600

H 2.40372900 0.75438400 -1.49270400

C 3.15081400 1.56436700 0.37516800

H 2.18401000 1.48224000 0.88373500

H 3.93018800 1.32852500 1.11298200

C 3.34669500 2.99332700 -0.14973300

H 4.30148600 3.06192300 -0.68967900

H 2.55861500 3.21526200 -0.87974300

C 3.31335200 4.03681300 0.97119700

H 4.10699600 3.85875800 1.70597800

H 3.44907200 5.04651900 0.57205600

H 2.35528300 4.01282000 1.50066100

**c-ts1**

SCF energy in solvent model (SMD): -2503.84630767 a.u.

Fe 1.76031200 0.53238000 -0.05961000

Cl 2.89596600 2.16789100 -1.06159900

Cl 0.68988600 -0.50490900 -1.72933500

Cl 1.29315100 -2.01054200 1.60403100

Cl 0.38331800 1.54643700 1.36823700

C 4.60966400 -0.74312700 -0.08288700

H 5.37452600 -1.09526100 0.61420300

H 4.94805700 0.16394900 -0.58182800

H 4.38944800 -1.51678600 -0.82476300

O 3.42627200 -0.40027200 0.66105100

H 2.99942600 -1.18693900 1.11524800

C -3.20123900 -2.68026500 -0.94236000

C -3.79421800 -1.52838700 -0.52189200

C -1.76693300 -1.61296300 0.36150500

N -1.93935800 -2.71564700 -0.37781400

H -3.56268300 -3.46763000 -1.58397100

H -4.77144200 -1.12247400 -0.72672200

H -0.85811200 -1.38645400 0.91624600

N -2.88440100 -0.87813900 0.29445000

C -0.91759100 -3.74613600 -0.60089000

H -0.05943300 -3.51824300 0.03503100

H -1.33307000 -4.72381800 -0.34823900

H -0.61055000 -3.72368900 -1.64826900

C -3.07706400 0.44401500 0.92262900

H -4.01761100 0.40596600 1.48175500

H -2.25759200 0.57670800 1.63124500

C -3.08060400 1.57861600 -0.10571700

H -2.13004200 1.55930100 -0.64992400

H -3.88011100 1.41088500 -0.84019300

C -3.26891000 2.94647600 0.56369800

H -4.20908600 2.95152200 1.13275000

H -2.46086700 3.10078900 1.28865500

C -3.27246100 4.09640600 -0.44816600

H -4.08187300 3.98385500 -1.17868600

H -3.40737200 5.05960800 0.05303400

H -2.32689500 4.13677000 -0.99876600

**c-2**

SCF energy in solvent model (SMD): -2503.84911964 a.u.

Fe -1.82919600 -0.88325100 -0.28402000

Cl -3.41902800 -2.22434200 -0.98996800

Cl -0.97952700 0.35942100 -1.92828700

Cl -1.02805600 2.32237100 2.18270800

Cl -0.20903700 -1.89636000 0.84022700

C -4.02125800 0.87570800 0.88337000

H -4.28708400 1.31887900 1.84546500

H -4.69449500 0.05155700 0.64720400

H -4.06843500 1.63633700 0.09859900

O -2.68644900 0.34279200 0.98767600

H -2.05761100 1.04603200 1.45515400

C 2.63277000 2.63131900 -1.49423000

C 3.37283200 1.58405900 -1.03398900

C 1.58305800 1.77422700 0.25393700

N 1.52490400 2.73601400 -0.67476700

H 2.79505200 3.29855600 -2.32531600

H 4.30433600 1.17346500 -1.38659900

H 0.83360800 1.64557900 1.03882800

N 2.70034100 1.06198500 0.05813700

C 0.41580800 3.68669300 -0.82574600

H -0.22493300 3.60174000 0.05529200

H 0.82100300 4.69709400 -0.90739500

H -0.15446300 3.43022500 -1.72066700

C 3.12354400 -0.09101600 0.87810500

H 4.02746300 0.20055500 1.42377700

H 2.32539200 -0.26100600 1.60353800

C 3.36216600 -1.35133300 0.04357200

H 2.44929500 -1.58120700 -0.51587300

H 4.16047400 -1.17080500 -0.68871900

C 3.74339000 -2.54559800 0.92925200

H 4.63639000 -2.29650300 1.51894500

H 2.93485500 -2.72808100 1.64753700

C 4.00096300 -3.81811600 0.11670700

H 4.82162300 -3.67760000 -0.59607000

H 4.26740600 -4.65390200 0.77053800

H 3.11044300 -4.10889200 -0.45016400

**c-3**

SCF energy in solvent model (SMD): -3308.03566676 a.u.

Fe -0.60789900 -2.43925400 -1.17617300

Cl -2.61872900 -2.22394400 -2.02813900

Cl 0.03656700 -4.55571300 -1.03121500

Cl 3.34940400 -1.23570700 -1.68170200

Cl -0.28188700 -1.38236600 0.75238600

C 0.58310500 -1.77795600 -3.87662800

H -0.44159700 -1.62625600 -4.21650500

H 0.91425800 -2.79367000 -4.10963000

H 1.24978000 -1.05133200 -4.34522200

O 0.61076700 -1.55694400 -2.44966700

H 1.59794000 -1.42890100 -2.13634200

C 3.24890700 -2.40597300 3.69624100

C 4.03941800 -1.31356400 3.50237900

C 3.27995000 -1.96333100 1.52744700

N 2.78774400 -2.79825900 2.45193000

H 2.97816800 -2.93174600 4.59772600

H 4.59196700 -0.71105400 4.20474000

H 3.11476200 -1.98505300 0.44695300

N 4.04771800 -1.05499400 2.14299400

C 1.87579700 -3.91700000 2.19028400

H 2.27298500 -4.81976600 2.65836100

H 0.88706600 -3.68393300 2.58856100

H 1.78950400 -4.06675400 1.11507200

C 4.77604200 0.04006100 1.46454400

H 5.83227100 -0.06203700 1.73477900

H 4.67136400 -0.14039600 0.39132700

C 4.24148400 1.42287300 1.84680500

H 3.19944500 1.50720600 1.51741600

H 4.24388700 1.53486100 2.93982700

C 5.08146600 2.54311300 1.21666000

H 6.13256300 2.41808100 1.51204200

H 5.04927300 2.45522800 0.12459800

C 4.59793400 3.93791300 1.62554700

H 4.63774300 4.07332500 2.71256200

H 5.22112100 4.71527900 1.17232100

H 3.56553900 4.10769000 1.30352100

C 2.10840200 2.38635200 -1.44625000

C 0.78821000 2.27968500 -1.03443300

C -0.02134500 3.42109500 -0.84318400

C 0.55534300 4.67894300 -1.09145400

C 1.88105100 4.80368600 -1.49843500

C 2.66146900 3.65606700 -1.67456700

H 2.71327800 1.49789800 -1.60693000

H 0.37723800 1.28972200 -0.86538800

H -0.04672200 5.57408600 -0.95937100

H 2.30702300 5.78818900 -1.68143300

O 3.97070500 3.70937100 -2.06031900

H 4.22141000 4.63075900 -2.20861600

C -1.41296300 3.35385000 -0.41153200

H -1.94692000 4.30244000 -0.36676600

C -2.11460200 2.25708300 -0.06197600

H -1.69407200 1.25766400 -0.05362100

C -3.52734000 2.38558900 0.32979000

O -4.16808400 3.41583300 0.39288500

O -4.02775600 1.13960200 0.61548100

C -5.35296500 0.91379300 0.97021600

C -6.12382900 1.79249400 1.73586300

C -5.86071300 -0.32269600 0.56408500

C -7.42274900 1.41420200 2.08340500

H -5.72306300 2.74986500 2.03669900

C -7.15800100 -0.68410900 0.92211200

H -5.22841600 -0.97974200 -0.02452700

C -7.94553900 0.18403700 1.68217400

H -8.02850000 2.09459200 2.67546700

H -7.55192400 -1.64540200 0.60493000

H -8.95753400 -0.09622000 1.95927600

**c-ts2**

SCF energy in solvent model (SMD): -3307.99884104 a.u.

Fe -2.72418600 0.14020400 0.36949900

Cl -3.97938900 -1.02828100 1.84508100

Cl -4.30356400 1.42028700 -0.58123000

Cl 1.20243200 1.16777600 2.20237200

Cl -0.91240600 1.22795600 -1.15683400

C -2.17063800 1.29262900 3.22180600

H -2.17641900 0.31500400 3.70652100

H -3.18331600 1.69487100 3.20679300

H -1.49227300 1.96995800 3.74652500

O -1.71993000 1.18115900 1.85276800

H -0.70893500 1.08975200 1.87074000

C -0.07080600 5.46276400 -1.07494200

C 1.27503500 5.25453300 -1.12577800

C 0.41460900 3.81171500 0.31286700

N -0.58793500 4.55636300 -0.16618600

H -0.69735900 6.16708400 -1.59799200

H 2.04408900 5.74794300 -1.69762500

H 0.35834700 2.99031800 1.02460700

N 1.55798500 4.22398500 -0.24611200

C -2.00853400 4.34801800 0.13630600

H -2.46946600 5.30672900 0.38143800

H -2.50465100 3.88436700 -0.71792900

H -2.08555300 3.66563300 0.98088900

C 2.87017200 3.58706200 -0.01585100

H 3.60148600 4.38867800 0.12860400

H 2.77636500 3.01874500 0.91339000

C 3.26903400 2.66017000 -1.16770900

H 2.48361700 1.90627400 -1.29346700

H 3.32095300 3.23042000 -2.10517100

C 4.61563500 1.97550000 -0.89793300

H 5.38971900 2.73812000 -0.73441000

H 4.54360900 1.39978800 0.03294700

C 5.04263500 1.04802200 -2.03971500

H 5.13961800 1.59535600 -2.98429200

H 6.00764300 0.57883100 -1.82497700

H 4.31037000 0.24791900 -2.18971900

C 4.43923900 -2.00840400 1.67112400

C 3.29228800 -1.68821300 0.96430400

C 3.01483700 -2.30457600 -0.27907900

C 3.93578000 -3.24729200 -0.77571600

C 5.08666500 -3.57495700 -0.06979000

C 5.34243500 -2.95509000 1.16023700

H 4.65548000 -1.53955200 2.62509000

H 2.61236400 -0.94759500 1.37861200

H 3.74088700 -3.73229500 -1.72867700

H 5.78444500 -4.30817800 -0.46806000

O 6.44754700 -3.22553800 1.90288600

H 6.98068000 -3.89740800 1.45729200

C 1.82923800 -2.01058900 -1.06424800

H 1.76156700 -2.52240400 -2.02298300

C 0.81539500 -1.17504500 -0.72002200

H 0.76825200 -0.61116000 0.20264400

C -0.25776200 -1.02905300 -1.66005900

O -0.58291100 -1.31323900 -2.74254700

O -1.86074400 -1.39350900 -0.42444900

C -2.32396600 -2.66849800 -0.58706200

C -3.24295600 -2.95729800 -1.60487500

C -1.83143700 -3.69499700 0.23050400

C -3.67392400 -4.27034200 -1.79159800

H -3.61060400 -2.14866600 -2.22815800

C -2.26821600 -5.00344800 0.03190700

H -1.12609300 -3.44828900 1.01723800

C -3.18893600 -5.29691700 -0.97793100

H -4.39202000 -4.49068300 -2.57651700

H -1.89216000 -5.79640300 0.67256700

H -3.52904900 -6.31763000 -1.12615800

**c-4**

SCF energy in solvent model (SMD): -3308.01566190 a.u.

Fe 0.00041900 3.16248400 -0.91741300

Cl 1.03492800 4.98773400 -1.62252200

Cl -2.22583200 3.39212300 -0.73083300

Cl -0.62217200 -0.90628300 -1.73514300

Cl -1.58392500 -2.46179100 2.52857800

C -0.06377400 2.04468000 -3.73246400

H 0.43440900 2.96841700 -4.02859000

H -1.14642100 2.14833500 -3.85092100

H 0.29923400 1.21173300 -4.33873100

O 0.27386800 1.79107000 -2.35479700

H 0.00200300 0.83409100 -2.10605800

C -4.42391600 0.77168400 1.95799600

C -5.02408600 0.07842100 0.95010800

C -2.84913000 0.11787200 0.55080300

N -3.06766500 0.78411800 1.68962100

H -4.83852500 1.25942300 2.82522100

H -6.06186400 -0.15231800 0.77255500

H -1.89585400 -0.06316500 0.05802700

N -4.02366100 -0.31877900 0.08124200

C -2.02665600 1.39391700 2.52168700

H -2.40101000 2.33964500 2.91526900

H -1.76851300 0.71608200 3.33834100

H -1.15073800 1.59107100 1.90360300

C -4.18832200 -1.13532700 -1.13759900

H -5.04411700 -0.72819100 -1.68498600

H -3.28387300 -0.98621400 -1.73344300

C -4.38017100 -2.62027500 -0.81635600

H -3.52175200 -2.96447000 -0.22770500

H -5.27250700 -2.75579700 -0.19003600

C -4.50608400 -3.45986100 -2.09457100

H -5.34954500 -3.09101300 -2.69445600

H -3.60545700 -3.31622800 -2.70411400

C -4.69841600 -4.95122200 -1.80263300

H -5.60815800 -5.12925600 -1.21797000

H -4.78041800 -5.52687300 -2.72970600

H -3.85352100 -5.35372500 -1.23371000

C 3.53780000 -2.44190100 -2.22192900

C 2.65361100 -2.52705700 -1.16075900

C 3.04640700 -3.12295400 0.06143700

C 4.35953900 -3.62447200 0.16563600

C 5.25172300 -3.54232700 -0.89500400

C 4.84195100 -2.94823500 -2.09640500

H 3.24144800 -1.98519100 -3.16002300

H 1.65165100 -2.12365600 -1.28236400

H 4.68042200 -4.08546300 1.09613000

H 6.26132900 -3.93482200 -0.79600000

O 5.66062400 -2.83422900 -3.17399300

H 6.52648000 -3.20879600 -2.96369200

C 2.16898000 -3.24235100 1.20666100

H 2.60423200 -3.71935600 2.08413900

C 0.87486600 -2.83782400 1.31820800

H 0.32584800 -2.35512200 0.51728100

C 0.19980700 -3.07678700 2.57947000

O 0.58259200 -3.57721700 3.59325300

O 0.66121200 2.34841800 0.57551900

C 1.69916200 2.36689800 1.43434900

C 2.03873200 1.17986200 2.10887800

C 2.42494000 3.54542900 1.68616300

C 3.09038200 1.17831900 3.02175000

H 1.48122800 0.27444800 1.89084700

C 3.47222700 3.52702500 2.60415300

H 2.15873500 4.45382400 1.15518700

C 3.81043000 2.34906200 3.27713500

H 3.34954500 0.25664200 3.53529000

H 4.02857100 4.44060700 2.79440500

H 4.62817500 2.34330400 3.99141300

**c-5**

SCF energy in solvent model (SMD): -3308.02005318 a.u.

Fe -0.80950700 -2.23892500 -0.71465700

Cl -0.18154900 -2.19230100 1.49746100

Cl -0.22577000 -0.24734800 -1.60545800

Cl 0.43517200 -3.84838400 -1.73063000

Cl 2.33502200 1.21592400 3.75996300

C -2.35206300 0.17268000 3.68979700

H -3.07764100 -0.63342300 3.51775100

H -2.89002700 1.04799000 4.06369100

H -1.64217200 -0.15187000 4.46212400

O -1.69527700 0.56120000 2.48936900

H -1.19062400 -0.20387300 2.16184400

C -2.87524400 3.65964600 -0.15721100

C -1.71635900 3.20337800 0.44564900

C -0.46173600 3.77571500 0.12541300

C -0.42670900 4.81196600 -0.82898500

C -1.58629400 5.27931300 -1.43241100

C -2.81873300 4.70259800 -1.09650000

H -3.83816900 3.21505100 0.06980600

H -1.77648800 2.37973500 1.15116800

H 0.52803200 5.25862400 -1.09437800

H -1.54065000 6.08307400 -2.16383800

O -3.99144200 5.10669200 -1.64835800

H -3.82228400 5.80748700 -2.29214400

C 0.78279500 3.33734600 0.71949400

H 1.68629700 3.77338500 0.29418200

C 0.95283400 2.47090900 1.75459700

H 0.12849900 2.00013800 2.27857100

C 2.30055500 2.14895800 2.17161100

O 3.35396400 2.39267000 1.63393800

O -2.58648000 -2.55653600 -0.85402900

C -3.82665600 -2.12403100 -0.59767300

C -4.06933400 -1.03712200 0.26568600

C -4.91109600 -2.78696500 -1.20402300

C -5.37968000 -0.63110100 0.50783500

H -3.23619500 -0.52621600 0.73886500

C -6.21330300 -2.36646600 -0.95100000

H -4.70262700 -3.62225100 -1.86478900

C -6.45683500 -1.28779800 -0.09502800

H -5.56097200 0.20670700 1.17602600

H -7.04377500 -2.88335200 -1.42422100

H -7.47451700 -0.96303600 0.10022100

C 4.08853800 -1.86182100 1.66952900

C 4.20700600 -0.64120300 1.07522900

C 3.00493100 -1.93582000 -0.25426500

N 3.34056000 -2.65736000 0.82149800

H 4.44485800 -2.21939200 2.62175200

H 4.65470000 0.27769600 1.41865700

H 2.38450700 -2.29104000 -1.06670400

N 3.52380600 -0.70852400 -0.12537100

C 2.89306500 -4.02645100 1.09727900

H 2.25078600 -4.01924600 1.97904600

H 2.31150800 -4.38042600 0.24637900

H 3.76325500 -4.66643100 1.25764200

C 3.39954400 0.38086600 -1.11504600

H 2.40406700 0.29279700 -1.55781900

H 3.43595000 1.31323700 -0.54684800

C 4.50096600 0.32938300 -2.17831700

H 5.48405300 0.37662900 -1.69166100

H 4.45358200 -0.63315800 -2.70413600

C 4.36412800 1.47645800 -3.18856200

H 3.37300300 1.42792900 -3.65729800

H 4.40617800 2.43514100 -2.65490600

C 5.44749000 1.44313200 -4.27096400

H 5.40654300 0.51035000 -4.84408700

H 5.32584600 2.27133000 -4.97564200

H 6.44925600 1.52187300 -3.83393400

**c-ts3**

SCF energy in solvent model (SMD): -3307.99614625 a.u.

Fe -2.07129400 1.17840600 -1.11957400

Cl -2.92641500 0.65124200 0.92611000

Cl -0.02378600 0.19365900 -1.31201100

Cl -3.41266200 0.27722200 -2.71466000

Cl 0.35913500 -2.09256900 2.79746200

C -0.62544400 1.25663000 3.89915100

H -1.66525900 1.49035700 3.66062800

H -0.10900500 2.15636900 4.23480400

H -0.57011600 0.48399200 4.67169100

O 0.02631200 0.81658100 2.69543000

H -0.41792700 -0.00834200 2.39411400

C 4.69345400 0.25904800 -1.95759100

C 3.89653900 0.19923600 -0.82760100

C 4.46868300 0.10636400 0.46203700

C 5.87279700 0.08223300 0.56292000

C 6.68086600 0.13918600 -0.56584000

C 6.09231600 0.22757500 -1.83384500

H 4.25721900 0.33571800 -2.94766100

H 2.81827700 0.23461100 -0.94750500

H 6.33495800 0.01448400 1.54421900

H 7.76374800 0.11686200 -0.46647600

O 6.81924100 0.28784400 -2.97953500

H 7.76191700 0.26796300 -2.76709200

C 3.68134300 0.04418700 1.68001800

H 4.25111700 0.02140900 2.60742700

C 2.32883100 0.01062400 1.77312000

H 1.65180100 0.00773000 0.92989800

C 1.74818200 -0.02739800 3.08252300

O 2.01212100 0.09084200 4.21496000

O -1.92548500 2.97987000 -1.29887700

C -1.35186300 4.06367500 -0.76290100

C -0.53322400 3.98157000 0.38119400

C -1.57847400 5.31611400 -1.36569300

C 0.04273300 5.13768200 0.90188300

H -0.36217500 3.01468600 0.84466600

C -0.99565500 6.46137400 -0.83155300

H -2.21153700 5.35909800 -2.24612300

C -0.18248700 6.38108900 0.30337400

H 0.67441000 5.06744900 1.78356200

H -1.17622400 7.42331900 -1.30367600

H 0.27081200 7.27731000 0.71638300

C -3.68229700 -3.12688800 1.28945200

C -2.37012600 -3.49327800 1.25611300

C -2.86201000 -2.53949700 -0.67396200

N -3.97226400 -2.53642400 0.07314000

H -4.41809600 -3.20843300 2.07267500

H -1.73000500 -3.91843000 2.01191900

H -2.77299200 -2.08442400 -1.65019100

N -1.87900300 -3.12424700 0.01843100

C -5.23617600 -1.89035100 -0.28760100

H -5.44748900 -1.10204500 0.43656200

H -5.12630900 -1.43358800 -1.27060500

H -6.03857500 -2.63141500 -0.29707300

C -0.49717000 -3.33681300 -0.45500400

H -0.31607300 -2.59803500 -1.23857500

H 0.15117900 -3.08880000 0.39053100

C -0.27058300 -4.76696800 -0.95281400

H -0.49804400 -5.47621700 -0.14612000

H -0.96801100 -4.98707800 -1.77227200

C 1.17355100 -4.97570400 -1.42835700

H 1.39831900 -4.25831900 -2.22836000

H 1.86063600 -4.74256800 -0.60509300

C 1.43199700 -6.40032800 -1.92821200

H 0.78034200 -6.64986300 -2.77315000

H 2.46766100 -6.51986400 -2.26082100

H 1.24909200 -7.13748500 -1.13835600

**c-6**

SCF energy in solvent model (SMD): -3308.03656047 a.u.

Fe 1.17427700 -1.75763200 -1.30764500

Cl 2.11810100 -2.14645200 0.72668100

Cl 0.21237300 0.31034100 -1.24552300

Cl 2.85321200 -1.64769600 -2.84161100

Cl 0.97798300 1.65375300 3.38760900

C -0.43757000 -1.85905600 3.69105600

H 0.64521500 -1.91842100 3.59311300

H -0.89664800 -2.80425000 3.39710800

H -0.73339000 -1.61507700 4.71413800

O -0.84712400 -0.81441200 2.78389800

H 0.23469100 0.64184500 2.99739900

C -3.98353400 3.05093800 -1.58018300

C -3.47562500 2.16263500 -0.64617500

C -4.27910500 1.67043300 0.40603400

C -5.61749200 2.09839000 0.46604700

C -6.13637300 2.99392200 -0.46318100

C -5.31801900 3.47630900 -1.49082000

H -3.37106300 3.42059900 -2.39571800

H -2.44892500 1.82453600 -0.74665700

H -6.25873300 1.72486600 1.26002100

H -7.17359600 3.31505300 -0.39487800

O -5.75944400 4.35199100 -2.43568200

H -6.69144400 4.55132400 -2.27633600

C -3.78860300 0.74009600 1.41326600

H -4.53918600 0.30987300 2.07532000

C -2.51177600 0.35580400 1.62206300

H -1.67914100 0.72313800 1.03238800

C -2.20951200 -0.63558600 2.65846500

O -2.99903100 -1.26608400 3.33443700

O -0.04452600 -3.04475600 -1.70803600

C -1.15239300 -3.62023400 -1.22196600

C -1.72714900 -3.20268900 -0.00579200

C -1.75358300 -4.66437000 -1.94993400

C -2.88324800 -3.81802300 0.46673500

H -1.25387900 -2.39876700 0.54789300

C -2.90736000 -5.27321200 -1.46393000

H -1.29877600 -4.97391200 -2.88542400

C -3.47886500 -4.85585700 -0.25741700

H -3.32036400 -3.47896200 1.40205500

H -3.36573600 -6.07859000 -2.03157100

H -4.38062300 -5.33339300 0.11413400

C 5.24057900 0.44566700 1.46280100

C 4.44658800 1.54862100 1.56131900

C 4.00787400 0.56472200 -0.36558100

N 4.95673100 -0.15096700 0.24884800

H 5.96080600 0.02987200 2.14855700

H 4.34035800 2.27475400 2.35033700

H 3.55969900 0.30680900 -1.31646000

N 3.68641200 1.60743100 0.40849200

C 5.49903800 -1.42655100 -0.23111300

H 5.18027400 -2.22248700 0.44358200

H 5.08957200 -1.62738500 -1.22080000

H 6.58835300 -1.36392000 -0.27245800

C 2.69300800 2.64783200 0.07596800

H 1.97673700 2.18896800 -0.60959100

H 2.15507000 2.87018800 1.00094300

C 3.33936200 3.90004600 -0.52321200

H 4.07767200 4.30875400 0.17980300

H 3.88958800 3.62365400 -1.43202100

C 2.29234400 4.97172400 -0.85661200

H 1.55530300 4.55123000 -1.55235200

H 1.73899900 5.23401500 0.05456900

C 2.91199500 6.23410000 -1.46371700

H 3.44140700 6.00754000 -2.39575600

H 2.14354000 6.97901600 -1.69079500

H 3.63018500 6.69535400 -0.77646600

**c-7**

SCF energy in solvent model (SMD): -3308.03842833 a.u.

Fe -2.21824600 -0.69903400 -0.26175000

Cl -3.08111400 0.96450000 -1.53341600

Cl -0.79995400 0.18370900 1.24409500

Cl -4.61900900 -0.18318000 3.19395500

Cl -1.01587300 -2.04952300 -1.63116000

C 3.67524500 0.57066400 -1.40383500

C 4.92187400 0.24238800 -0.89266400

C 5.13902900 -0.97952100 -0.22372600

C 4.04416800 -1.85407300 -0.09280700

C 2.78778500 -1.53845700 -0.59902200

C 2.60098000 -0.31918500 -1.25892300

H 3.51614400 1.50970600 -1.92514800

H 5.74154800 0.94319800 -1.01701800

H 4.18381300 -2.80157600 0.42054600

H 1.95416000 -2.22425100 -0.48161000

O 1.39370200 0.05529900 -1.77834300

H 0.73711900 -0.66616900 -1.68193100

C 6.42962500 -1.37366900 0.33053700

H 6.46097500 -2.34491600 0.82264400

C 7.58759900 -0.68512300 0.30891300

H 7.69065200 0.29296400 -0.14921900

C 8.79711200 -1.25571200 0.92740700

O 8.88144600 -2.33462400 1.48598100

O -3.56922700 -1.61024200 0.60432400

C -4.45914800 -2.55898300 0.21445900

C -5.01295800 -2.55166300 -1.07639400

C -4.84092100 -3.54642600 1.13702400

C -5.93333800 -3.53400800 -1.43582100

H -4.72378700 -1.77273300 -1.77516800

C -5.76380500 -4.52009700 0.76312800

H -4.40628200 -3.53281700 2.13121000

C -6.31303300 -4.52102100 -0.52226000

H -6.35868700 -3.52513100 -2.43541500

H -6.05499500 -5.28250900 1.48006200

H -7.03176700 -5.28281700 -0.80862300

C -1.05602300 4.31523100 -2.58948700

C -1.15794300 4.76341700 -1.30697500

C -0.22925400 2.76006800 -1.25612800

N -0.46509800 3.06734600 -2.53762600

H -1.35021000 4.76923200 -3.52190000

H -1.55017300 5.68623600 -0.91219900

H 0.21267300 1.83531500 -0.91083100

N -0.63249300 3.77949100 -0.48918300

C -0.27756800 2.15558400 -3.67222200

H 0.06361900 2.73054000 -4.53442200

H 0.46756500 1.40892000 -3.39884900

H -1.22696200 1.66153500 -3.89046500

C -0.59193900 3.81304400 0.98827700

H -0.02517500 2.93556400 1.30370900

H -0.03537300 4.70980000 1.27965000

C -1.99142700 3.79349700 1.60867200

H -2.56135900 4.66777700 1.26635400

H -2.52041600 2.90276400 1.25437000

C -1.92405000 3.78865000 3.14207600

H -1.36471900 2.90414200 3.47059800

H -1.35855100 4.66550200 3.48794000

C -3.31367300 3.77970000 3.78655200

H -3.87339900 2.88504100 3.49685300

H -3.23702600 3.78616600 4.87802400

H -3.89682700 4.65857500 3.48790100

C 11.07901500 -0.87185400 1.36908800

H 10.96747800 -1.04265700 2.44329300

H 11.81065200 -0.08534600 1.18172900

H 11.39473800 -1.80762300 0.89985500

O 9.85004100 -0.40554100 0.79773100

H -4.12854600 -0.77484700 2.12941200

**c-ts4**

SCF energy in solvent model (SMD): -3308.03315416 a.u.

Fe 2.36279600 0.63996600 -0.79149100

Cl 2.99855700 -1.16711100 -1.91785900

Cl 0.94903600 0.21568100 0.87999700

Cl 4.75228900 -0.09590700 1.25507700

Cl 1.06062900 1.75862900 -2.27078800

C -3.43631100 -1.11993300 -1.27282300

C -4.67007900 -0.75550500 -0.75486100

C -4.92512300 0.56573100 -0.33434800

C -3.88139200 1.50154100 -0.45859300

C -2.63852200 1.15103400 -0.97506100

C -2.41323800 -0.16726900 -1.38523100

H -3.24970700 -2.13748400 -1.60238300

H -5.44994400 -1.50678700 -0.67845400

H -4.05057200 2.52641800 -0.13952300

H -1.84389200 1.88664000 -1.05507100

O -1.21636800 -0.58126200 -1.89935400

H -0.60667600 0.17654100 -1.99749900

C -6.20422300 1.00138600 0.21553900

H -6.26765800 2.05018200 0.50273500

C -7.31682200 0.26728200 0.41183600

H -7.38532400 -0.78768400 0.16787500

C -8.52226400 0.89290500 0.98352600

O -8.64044700 2.05772700 1.31880100

O 3.81880000 1.91879100 -0.36034000

C 3.72585200 3.24419400 0.05614000

C 3.44738600 4.22605600 -0.89490800

C 3.93998800 3.56553400 1.39868000

C 3.37670700 5.55780000 -0.48678200

H 3.28942800 3.94087000 -1.92898900

C 3.87374500 4.90384000 1.78639100

H 4.15740500 2.77699100 2.11170300

C 3.59031200 5.90125900 0.85047300

H 3.15887200 6.32879800 -1.21991200

H 4.04231400 5.16420300 2.82715100

H 3.53681100 6.94027300 1.16102600

C 1.28848600 -4.98046700 -1.48236700

C 1.59697400 -4.98519300 -0.15541800

C 0.58232300 -3.07773600 -0.60963500

N 0.64879600 -3.78403000 -1.74581300

H 1.46911400 -5.71293100 -2.25245200

H 2.09182000 -5.72683300 0.44958200

H 0.15157100 -2.09083600 -0.51447400

N 1.14562500 -3.78958800 0.37308000

C 0.23745000 -3.30247800 -3.06935900

H -0.28736200 -4.10447500 -3.59150400

H -0.42028200 -2.44338000 -2.93931600

H 1.12307500 -2.99764100 -3.63064600

C 1.29667400 -3.35564300 1.78132700

H 0.80690300 -2.38362500 1.85892200

H 0.74677300 -4.07256700 2.40042400

C 2.76197500 -3.25187500 2.21097500

H 3.25412200 -4.22672500 2.08989800

H 3.28240200 -2.53558400 1.56604500

C 2.87707800 -2.79941900 3.67388700

H 2.41587900 -1.80941600 3.77449400

H 2.30588200 -3.48242200 4.31935300

C 4.33260600 -2.72965500 4.14594000

H 4.89919700 -2.01833000 3.53821700

H 4.38833700 -2.40580600 5.18999000

H 4.82090300 -3.70880100 4.07454700

C -10.74833400 0.49735500 1.64528400

H -10.58776200 0.88819600 2.65373800

H -11.44008200 -0.34520800 1.66830000

H -11.14588700 1.30175300 1.02047200

O -9.52694200 -0.01558000 1.09795700

H 4.36417200 1.25807200 0.33419200

**c-8**

SCF energy in solvent model (SMD): -3308.05825691 a.u.

Fe 2.24435700 0.19748500 -0.89590000

Cl 3.30239000 1.61970800 0.46327400

Cl 0.45701500 1.26245100 -1.72966200

Cl 3.58564600 -0.52913900 -2.50361900

Cl 1.45995400 -1.52285000 0.33553900

C -3.57484500 0.05790200 1.52029000

C -4.85354400 -0.30857000 1.12802900

C -5.06135800 -1.27915400 0.12672900

C -3.92404700 -1.86398100 -0.46037600

C -2.63531600 -1.50586400 -0.07829500

C -2.45920100 -0.53959500 0.91706300

H -3.42017100 0.80035500 2.29714000

H -5.70520000 0.16340500 1.60789000

H -4.05610800 -2.61407700 -1.23533900

H -1.77076100 -1.96570200 -0.54819000

O -1.22096500 -0.13870900 1.33705800

H -0.51874600 -0.65969200 0.89559400

C -6.38459400 -1.69949400 -0.32238800

H -6.40486300 -2.45968400 -1.10220700

C -7.58404700 -1.26282800 0.10818000

H -7.70209000 -0.50892600 0.87944800

C -8.82177000 -1.81285500 -0.47314100

O -8.89414000 -2.66133400 -1.34361700

O 5.69495500 -1.76024100 -0.14305000

C 5.70298600 -3.11481700 0.01057200

C 6.46137700 -3.63838200 1.06583200

C 4.99712900 -3.97985700 -0.83592400

C 6.50923900 -5.01580400 1.26835500

H 7.00286400 -2.95168000 1.70877000

C 5.05399900 -5.35746700 -0.62091300

H 4.40771300 -3.57241000 -1.65318800

C 5.80764900 -5.88566500 0.42857800

H 7.10133600 -5.41260700 2.08893300

H 4.50186300 -6.01971700 -1.28242700

H 5.84848500 -6.95854700 0.59036100

C 1.49996800 3.54060900 3.24440600

C 1.23398100 4.47834300 2.29214600

C 0.24923700 2.59616600 1.68886900

N 0.86857400 2.37567400 2.85459300

H 2.08106900 3.59975200 4.15042800

H 1.53295700 5.51097100 2.21915900

H -0.31870600 1.85478700 1.14220600

N 0.44876000 3.86946000 1.33034400

C 0.98635400 1.06812800 3.51197500

H 0.94687900 1.21156800 4.59265000

H 0.15745300 0.43982300 3.18732900

H 1.93284100 0.60652000 3.22162600

C -0.02747900 4.48657900 0.07507800

H -0.64411400 3.73602100 -0.42305600

H -0.66535500 5.33506700 0.34382800

C 1.12559800 4.91611100 -0.83598900

H 1.73670800 5.67480000 -0.32927700

H 1.76963600 4.04964100 -1.01743700

C 0.60855800 5.47596700 -2.16821800

H 0.01275500 4.70433200 -2.67138000

H -0.06855000 6.32010900 -1.97709500

C 1.74491300 5.92577300 -3.09168600

H 2.41397100 5.09139600 -3.32613900

H 1.35218200 6.31445100 -4.03593900

H 2.34552700 6.71694400 -2.62889500

C -11.17428900 -1.71137200 -0.40227800

H -11.26372000 -1.51697200 -1.47450800

H -11.93640800 -1.16112200 0.15025800

H -11.28113600 -2.78643200 -0.23413800

O -9.91527400 -1.24128100 0.09656700

H 5.15236600 -1.51249900 -0.90862300

**MPC+cat+phenol**

SCF energy in solvent model (SMD): -3308.06488087 a.u.

C -2.92444200 3.87091700 0.27652700

C -2.40061500 2.59134000 0.32163300

C -2.62350200 1.67710100 -0.72656600

C -3.37325600 2.11029000 -1.83110100

C -3.90942600 3.39058000 -1.88387100

C -3.68769900 4.27412500 -0.82450500

H -2.74210000 4.57930100 1.07513400

H -1.78560000 2.30772500 1.16618200

H -3.54910800 1.42579000 -2.65394400

H -4.49342000 3.70597800 -2.74335600

O -4.18053500 5.54675000 -0.80964700

H -4.67111100 5.72114100 -1.61979900

C -2.10169200 0.32283800 -0.71300000

H -2.11762600 -0.21118900 -1.65845300

C -1.62783700 -0.34685400 0.35495100

H -1.62411900 0.09400000 1.33955400

C -1.12247200 -1.70345700 0.17340700

O -0.79652000 -2.18393600 -0.90060900

O -3.12907100 -4.26193100 -0.06054600

C -3.91255800 -3.13897400 -0.17923000

C -4.38241700 -2.44999500 0.94119400

C -4.24731000 -2.69771800 -1.45870800

C -5.15572100 -1.30230000 0.77863600

H -4.14107300 -2.82016000 1.93139500

C -5.03056500 -1.55655000 -1.61182100

H -3.86272800 -3.24161000 -2.31251400

C -5.48000800 -0.84725800 -0.49740000

H -5.50303300 -0.76262200 1.65255900

H -5.27948600 -1.21137600 -2.60961100

H -6.06914200 0.05322500 -0.62258900

C 3.84279200 -3.52425800 -0.96567400

C 4.37953800 -2.44406900 -1.59250800

C 2.21277200 -2.08409700 -1.34884000

N 2.48884200 -3.28717700 -0.83369200

H 4.29944600 -4.42790800 -0.60186100

H 5.39194400 -2.22902800 -1.88374000

H 1.23788100 -1.62068300 -1.35117800

N 3.34404600 -1.55966000 -1.82993700

C 1.54910400 -4.13967500 -0.09611000

H 1.77129200 -5.18118600 -0.32614200

H 1.66396900 -3.94563300 0.97035800

H 0.53524500 -3.88471000 -0.39391900

C 3.48025100 -0.23214000 -2.46159200

H 3.82762400 -0.38817600 -3.48645500

H 2.48153800 0.20047900 -2.49462100

C 4.42648100 0.67858800 -1.68222300

H 4.09885500 0.72878300 -0.64148700

H 5.43712800 0.25288400 -1.68136700

C 4.45947400 2.08874400 -2.27987800

H 4.72606100 2.03282600 -3.34337700

H 3.45210700 2.51294700 -2.22486400

C 5.44133500 3.00477700 -1.54471800

H 6.46364600 2.61661400 -1.60205300

H 5.44005200 4.00836900 -1.97776600

H 5.17125500 3.09017100 -0.48988100

Fe 1.79680500 1.03904100 1.49454500

Cl 3.68497200 2.09578900 1.94049100

Cl 1.14637800 1.51609500 -0.59015600

Cl 2.16489400 -1.17835500 1.62399900

Cl 0.18160500 1.54736000 2.94262300

C -1.21171300 -2.02156300 2.59880800

H -0.45523200 -1.27071600 2.82453700

H -1.07599500 -2.88314200 3.25014000

H -2.21426900 -1.61201000 2.73238800

O -1.03715600 -2.53033800 1.25760900

H -2.44139400 -4.08913700 0.59676700
